# Non-contact Anterior Cruciate Ligament Injury Epidemiology in Team-Ball Sports: A Systematic Review with Meta-analysis by Sex, Age, Sport, Participation Level, and Exposure Type

**DOI:** 10.1007/s40279-022-01697-w

**Published:** 2022-05-27

**Authors:** Lionel Chia, Danilo De Oliveira Silva, Matthew Whalan, Marnee J. McKay, Justin Sullivan, Colin W. Fuller, Evangelos Pappas

**Affiliations:** 1 Faculty of Medicine and Health, Sydney School of Health Sciences, Discipline of Physiotherapy, The University of Sydney, Sydney, NSW Australia; 2 Cleveland Guardians Baseball Company, Cleveland, OH USA; 3 La Trobe Sport and Exercise Medicine Research Centre, School of Allied Health, Human Services and Sport, La Trobe University, VIC, Australia; 4 Research and Development Department, Football Australia, Sydney, NSW Australia; 5 Centre of Medical and Exercise Physiology, School of Medical, Indigenous & Health Sciences, University of Wollongong, Wollongong, NSW Australia; 6 Colin Fuller Consultancy Ltd, Sutton Bonington, UK; 7 School of Medicine and Illawarra Health and Medical Research Institute, The University of Wollongong, Wollongong, NSW Australia

## Abstract

**Background:**

Not all anterior cruciate ligament (ACL) injuries are preventable. While some ACL injuries are unavoidable such as those resulting from a tackle, others that occur in non-contact situations like twisting and turning in the absence of external contact might be more preventable. Because ACL injuries commonly occur in team ball-sports that involve jumping, landing and cutting manoeuvres, accurate information about the epidemiology of non-contact ACL injuries in these sports is needed to quantify their extent and burden to guide resource allocation for risk-reduction efforts.

**Objective:**

To synthesize the evidence on the incidence and proportion of non-contact to total ACL injuries by sex, age, sport, participation level and exposure type in team ball-sports.

**Methods:**

Six databases (MEDLINE, EMBASE, Web of Science, CINAHL, Scopus and SPORTDiscus) were searched from inception to July 2021. Cohort studies of team ball-sports reporting number of knee injuries as a function of exposure and injury mechanism were included.

**Results:**

Forty-five studies covering 13 team ball-sports were included. The overall proportion of non-contact to total ACL injuries was 55% (95% CI 48–62, *I*^2^ = 82%; females: 63%, 95% CI 53–71, *I*^2^ = 84%; males: 50%, 95% CI 42–58, *I*^2^ = 86%). The overall incidence of non-contact ACL injuries was 0.07 per 1000 player-hours (95% CI 0.05–0.10, *I*^2^ = 77%), and 0.05 per 1000 player-exposures (95% CI 0.03–0.07, *I*^2^ = 97%). Injury incidence was higher in female athletes (0.14 per 1000 player-hours, 95% CI 0.10–0.19, *I*^2^ = 40%) than male athletes (0.05 per 1000 player-hours, 95% CI 0.03–0.07, *I*^2^ = 48%), and this difference was significant. Injury incidence during competition was higher (0.48 per 1000 player-hours, 95% CI 0.32–0.72, *I*^2^ = 77%; 0.32 per 1000 player-exposures, 95% CI 0.15–0.70, *I*^2^ = 96%) than during training (0.04 per 1000 player-hours, 95% CI 0.02–0.07, *I*^2^ = 63%; 0.02 per 1000 player-exposures, 95% CI 0.01–0.05, *I*^2^ = 86%) and these differences were significant. Heterogeneity across studies was generally high.

**Conclusion:**

This study quantifies several key epidemiological findings for ACL injuries in team ball-sports. Non-contact ACL injuries represented over half of all ACL injuries sustained. The proportion of non-contact to total ACL injuries and injury incidence were higher in female than in male athletes. Injuries mostly occurred in competition settings.

**Supplementary Information:**

The online version contains supplementary material available at 10.1007/s40279-022-01697-w.

## Key Points

**Table:** 

The overall proportion of non-contact to total ACL injuries in team ball-sports was 55%.
Injury incidence of non-contact ACL injuries in team ball-sports was higher in female athletes than in male athletes.
Injury incidence of non-contact ACL injuries in team ball-sports during competition was higher than during training.

## Introduction

Anterior cruciate ligament (ACL) injuries commonly occur in team ball-sports [1–3] but we do not know how many of these injuries are preventable. ACL injuries that result from contact situations like a tackle are sometimes unavoidable [4] compared to those that occur in non-contact situations like twisting and turning in the absence of external contact [5]. Exercise-based injury risk reduction programs (IRRPs) are a prominent feature in ACL injury risk-reduction efforts [6] and these programs seem to have a stronger effect on reducing the risk of non-contact ACL injuries (odds ratio (OR) 0.39) compared with contact ones (OR 0.61) [5]. Syntheses of information about the epidemiology of non-contact ACL injuries are currently unavailable and this information is important for guiding ACL risk-reduction efforts [7, 8]. Prior epidemiological reviews on ACL injury incidence did not consider injury mechanism [1, 9], combined different exposure types (player-hours converted to player-exposures) [10, 11], did not utilize meta-regression analyses to investigate sources of heterogeneity and the association of categorical variables like sex and sport, and did not investigate the proportion of non-contact to total ACL injuries.

We need to better understand the extent of non-contact ACL injuries because they impose a wide-ranging personal, societal and economic burden [12–14]. ACL injuries are associated with, for example, a sevenfold increase in odds of end-stage osteoarthritis resulting in total knee arthroplasties [12], more than US$90,000 per injury to gain a quality-adjusted life-year [15], and psychological barriers that may affect recovery, return to sport and an increased risk of sustaining a subsequent injury [16]. Therefore, we undertook a comprehensive systematic review, meta-analysis and meta-regression to estimate the proportion of non-contact ACL to total ACL injuries, and describe the incidence of non-contact ACL injuries by unit of exposure, sex, age group, sport, participation level, and exposure type in team ball-sports.

## Methods

This review is on ACL injuries only and it forms part of a larger systematic review on the epidemiology of non-contact knee injuries sustained in team ball-sports. Future publications will focus on other non-contact knee injuries like gradual-onset knee injuries. The review was prepared and conducted according to the Preferred Reporting Items for Systematic Reviews and Meta-Analyses (PRISMA) 2020 statement [17], and was prospectively registered with the PROSPERO International Prospective Register of Systematic Reviews (CRD42020179475). We were informed in an automated PROSPERO message that due to their focus on COVID-19-related systematic review registrations at the time of registration, this submission was automatically published and not checked for eligibility. Patients and public partners were not involved in the design, conduct or interpretation of this systematic review.

### Search Strategy and Selection Criteria

Six electronic databases (MEDLINE, EMBASE, Web of Science, CINAHL, Scopus and SPORTDiscus) were systematically searched from inception to July 2021. Search terms consisted of controlled vocabulary and free text, and were mapped to medical subject where possible to capture records of knee injury (e.g., “anterior cruciate ligament rupture”, “patellofemoral pain”, “meniscus tears”) epidemiology (e.g., “prevalence”, “incidence”, “exposure”) in team ball-sports (e.g., “soccer”, “football”, “rugby”, “basketball”). The MEDLINE search strategy is provided in Appendix A1. All records were downloaded to EndNote X8 (Thomson Reuters, USA) where duplicates were removed, then uploaded to Covidence (Covidence systematic review software, Veritas Health Innovation, Melbourne, VIC, Australia; http://www.covidence.org). Bibliographic hand-searches were also performed to supplement the electronic database search.

Studies were included if: (i) the number of ACL injuries as a function of injury mechanism were reported; (ii) they were prospective cohort studies or retrospective cohort studies examining routinely collected data (e.g., league-wide injury surveillance databases and insurance databases); (iii) they featured athletes from field and court-based team ball-sports (Association Football or soccer, futsal, football or American Football, rugby union, rugby league, Gaelic football, Australian football, basketball, netball, handball, volleyball, field hockey, floorball, lacrosse, hurling, baseball, softball, and cricket) because ACL injury mechanisms on these surfaces were more comparable [4, 18, 19]; (iv) they reported exposure data in terms of athlete-hours, athlete-exposures, or per-event data. Examples of per-event exposure data are the number of tackles in rugby union or number of jumps in volleyball. If a study reported on knee injuries and injury mechanisms separately, but not knee injury as a function of mechanism, study authors were contacted via email to request more information. We excluded studies if more detailed data were not available or authors did not provide the information following two email contact attempts. Studies were also excluded if: (i) the data had been published in earlier papers, such as cases of secondary analysis of routinely collected data; in such situations, the paper that reported the most exposure data was included; (ii) they investigated non-organised or non-competitive sport, such as school-based recreation or physical education classes; (iii) the data recorded were not sport-specific (e.g., hospital emergency department admission records); (iv) they investigated post-surgical populations or re-injury outcomes; (v) they featured athletes competing on ice, sand, in water, or on horseback. Studies were not excluded based on their definition of non-contact injury mechanisms, or lack thereof. Two authors (LC and DS) independently applied selection criteria to screen studies by titles and abstracts, followed by full texts to identify eligible studies. Disagreements were settled through discussion and consensus, and a third author (EP) acted as a tie-breaker if needed.

### Quality Assessment

Two authors (LC and MW) independently assessed study quality using a modified six-item Newcastle–Ottawa scale for cohort studies where one star was awarded for each item for a maximum of six stars [20]. A similar scale was previously used in a systematic review of acute hamstring injuries [21]. The six items were: (a) population description, (b) population recruitment, (c) surveillance methods, (d) duration of observation, (e) case definition, and (f) others (all other methods) (Appendix A2). Disagreement between assessors was settled through discussion and consensus, with a third author (EP) acting as a tie-breaker if needed. In accordance with previously published systematic reviews and meta-analyses of injury incidence [22, 23], the Grading of Recommendations Assessment, Development and Evaluation (GRADE) system to assess certainty of evidence was not used because this review is not a clinical practice guideline and does not make clinical recommendations [24].

### Data Extraction and Management

Publication information (authors, year), population characteristics (cohort size, age, participation level, sport, sex), exposure type (training, competition, composite), number of injuries, exposure, unit of exposure, surveillance information (definition of injury, how injuries were recorded, duration of surveillance), and severity of injuries were extracted and recorded on a customised spreadsheet by one author (LC), and double-checked by another author (DS). Disagreements were resolved by a third author (MW). Sample populations were classified into three age groups: children (≤ 12 years), adolescents (13–18 years), and adults (≥ 19 years). Participation level was classified into three categories: amateur (including recreational, high school and intramural athletes), intermediate (including collegiate and semi-professional athletes), and elite (including professional and national-level athletes) [9]. If not explicitly reported, incidences (per 1000 exposure units) were calculated from the available raw data using the following formula:

$$\mathrm{Incidence}=\frac{\mathrm{Sum \, of \, new \, knee \, injuries \, over \, a \, specified \, time}}{\mathrm{Sum \, of \, exposure \, units \, for \, all \, included \, samples}}\times 1000.$$

### Statistical Analysis

All meta-analyses were performed in R (V. 3.6.1 and later, the R Foundation for Statistical Computing) using the *meta* (*metarate, metaprop, metareg,* and *forest.meta* functions) and *tidyverse* packages. Meta-analyses of incidence were carried out using a random effects Poisson regression model (unconditional model – random study effects) to produce forest plots with 95% confidence intervals (CIs) [25, 26]. The Poisson regression model was selected because of the binary and frequentist nature of the incidence data, corresponding to similar, previously employed methods [22, 27]. Statistical heterogeneity was assessed using the *I*^2^ statistic where *I*^2^ < 50% was considered as not important, 50–75% as moderate, and > 75% as high heterogeneity [28]. Between-study variance was estimated using the maximum-likelihood method. Meta-analyses were only performed when there were three or more included studies. Meta-analyses of proportions were performed using the Freeman-Tukey Double arcsine transformation [29, 30]. Confidence intervals for individual studies were calculated using the Clopper-Pearson interval, and estimations of between-study variance were performed using the DerSimonian-Laird method. The α level for all meta-analyses was set at 0.05. Funnel plots were used to assess publication bias in studies included in meta-analyses of overall proportion of non-contact to total ACL injuries and overall incidence of non-contact ACL injuries. Like previous systematic reviews [31, 32], we conducted subgroup and meta-regression analyses to investigate sources of heterogeneity and the association of the following categorical variables with proportion and incidence of non-contact ACL injuries: exposure type unit, sex, sport, age group, participation level, and exposure type (competition vs. training). For all analyses except meta-analyses of incidence by exposure type, we only synthesised studies when both training and competition data together were available. Additional sub-group analyses were attempted where possible.

## Results

**Fig. 1:**
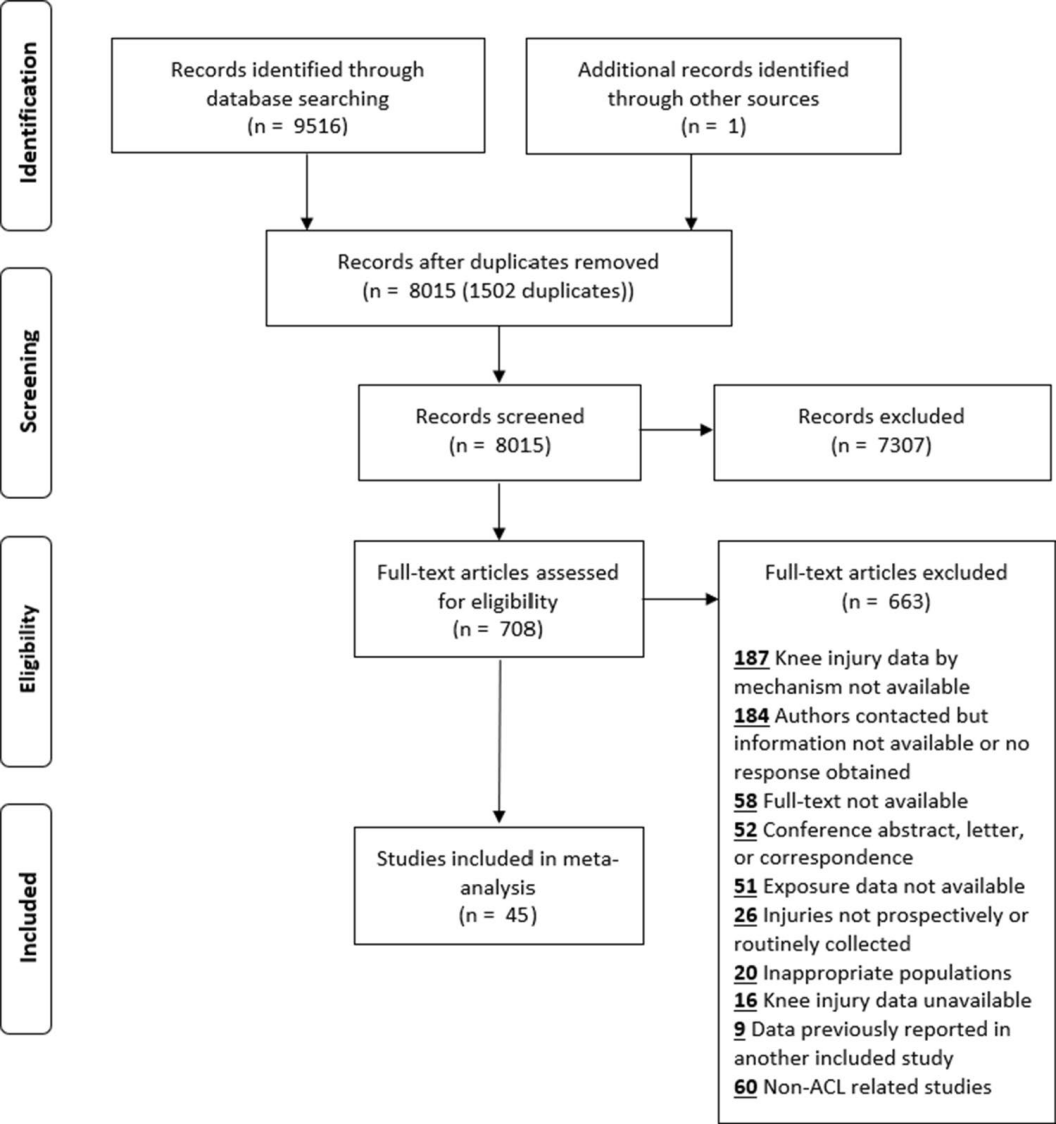
Flow chart of the study selection process

The online database and bibliographic hand search yielded 8,015 non-duplicate studies that were screened by title and abstract: 708 potentially eligible studies were identified. Following full-text review of the 708 studies, 45 met the inclusion criteria and were included in this review (Fig. 1) [33–77]. Two studies shared the same dataset [43, 44], and therefore only 44 studies are reflected in Tables 1 and 2.

**Table 1:** Characteristics of the included studies

Study	Year	Sport(s)	Level	Study duration	Sex	No. of athletes	Age	No. of non-contact ACL injuries	Incidence^a^	Unit	Proportion (%)	Non-contact injury definition
Agel et al. [47]	2005	Basketball, soccer	I	13	M/F	-	AD	Soccer (M): 66 Soccer (F): 161 Basketball (M): 78 Basketball (F): 305	Soccer (M): 0.04 Soccer (F): 0.13 Basketball (M): 0.04 Basketball (F): 0.17	pex	Soccer (M): 34 Soccer (F): 41 Basketball (M): 46 Basketball (F): 59	No apparent contact, contact with the ball, or contact with the floor
Agel et al. [45]	2007	Basketball	I	16	F	-	AD	169	0.42	pex	64	No apparent contact, contact with the ball, or contact with the floor
Anderson et al. [48]	2019	Basketball, lacrosse, soccer	I	12	M/F	-	AD	Basketball (M): 45 Basketball (F): 127 Lacrosse (M): 41 Lacrosse (F): 50 Soccer (M): 12 Soccer (F): 45	Basketball (M): 0.05 Basketball (F): 0.15 Lacrosse (M): 0.09 Lacrosse (F): 0.14 Soccer (M): 0.02 Soccer (F): 0.05	pex	Basketball (M): 57 Basketball (F): 69 Lacrosse (M): 69 Lacrosse (F): 71 Soccer (M): 35 Soccer (F): 44	-
Brooks et al. [37]	2005	Union	E	2	M	63	AD	1	0.12	phr	100	Twisting/turning, running, lifting (lineout/kickoff)
Dallalana et al. [49]	2007	Union	E	2	M	546	AD	2	0.01	phr	22	Twisting/turning, running, lifting, lineout, other non-contact (not defined)
Dick et al. [46]	2007	Am football	I	16	M		AD	694	0.06	pex	32	No apparent contact
Dönmez et al. [50]	2018	Soccer	A	1	M	1821	AD	2	0.35	phr	13	-
Faude et al. [51]	2005	Soccer	E	1	F	165	AD	7	0.20	phr	64	Running, change in direction, shooting, jumping, hit by ball, others (not defined)
Fuller et al. [43, 44]	2007	Soccer	I	2	M/F		AD	M: 11, F: 42	M: 0.04, F: 0.13	phr	M: 48, F: 46	No player-to-player or player-surface contact
Fuller et al. [39]	2008	Union	E	1	M	626	AD	0	0.00	phr	0	No player-to-player or player-surface contact
Fuller et al. [36]	2010	Union	I	2	M	282	AD	1	0.42	phr	17	No player-to-player or player-surface contact
Fuller et al. [35]	2013	Union	E	1		615	AD	2	1.04	phr	40	No player-to-player or player-surface contact
Fuller et al. [34]	2017	Union	E	1	M	639	AD	4	0.21	phr	80	No player-to-player or player-surface contact
Fuller et al. [42]	2018	Union	I	8	M	3922	AD	4	0.30	phr	24	No player-to-player or player-surface contact
Fuller et al. [33]	2020	Union	E	1	M	646	AD	0	0.00	phr	0	No player-to-player or player-surface contact
Fuller and Taylor [41]	2020	Sevens	E	10	M	3242	AD	12	1.00	phr	39	No player-to-player or player-surface contact
Giza et al. [52]	2005	Soccer	E	2	F	202	AD	6	0.07	phr	75	-
Gupta et al. [53]	2020	Soccer	A	10	M/F	-	ADO	M: 27, F: 106	M: 0.02, F: 0.07	pex	M: 36, F: 53	No player-to-player or player-surface contact
Hartmut et al. [54]	2010	Soccer	E	1	F	254	AD	8	0.11	phr	73	Twisting, contact with turf, taking a shot, sprinting, or falling
Hollander et al. [55]	2018	Hockey	A	1	M/F	M: 158, F: 74	AD	M: 0, F: 1	M: 0.00, F: 0.09	phr	M: 0, F: 100	No contact with another player, ball or stick
Joseph et al. [56]	2013	Am football, soccer, volleyball, basketball, baseball, softball	A	5	M/F	-	ADO	Am football (M): 87 Soccer (M): 14 Soccer (F): 44 Volleyball (F): 6 Basketball (M): 13 Basketball (F): 42 Baseball (M): 4 Softball (F): 12	Am football (M): 0.03 Soccer (M): 0.02 Soccer (F): 0.06 Volleyball (F): 0.01 Basketball (M): 0.01 Basketball (F): 0.05 Baseball (M): < 0.01 Softball (F): 0.02	pex	-	No contact with another player, surface, or apparatus (ball, base, goalpost etc.)
Krutsch et al. [57]	2016	Soccer	I	1	M	408	AD	6	0.04	phr	38	-
Leppanen et al. [58]^b^	2017	Basketball	I	3	F	-	ADO	M: 1, F: 3	M: 0.04, F: 0.12	phr	-	No direct contact or strike to the involved knee
Leyes et al. [59]	2011	Soccer	I	3	F	55	AD, ADO	AD: 7, ADO: 4	AD: 0.26, ADO: 0.38	phr	AD: 100, ADO: 100	Absence of direct trauma against another player
Loughran et al. [60]	2019	Am football	I	10	M	-	AD	191	0.06	pex	37	No apparent contact with another player, playing surface, and other
Nilstad et al. [61]	2014	Soccer	E	1	F	173	AD	5	0.11	phr	-	-
O’Connor et al. [77]	2021	Gaelic	A	2	F	132	AD	0	0.00	phr	-	-
Orchard et al. [62]	2001	Au football	E	8	M	1643	AD	63	0.47	pex	76	Absence of direct contact to the injured knee or leg
Ostenberg and Roos [63]	2000	Soccer	I	1	F	123	AD	0	0.00	phr	0	-
Pasanen et al. [64]	2008	Floorball	I	1	F	374	AD	7	0.15	phr	70	-
Pasanen et al. [65]	2017	Floorball	E	4	M/F	-	AD	M: 0, F: 1	M: 0.00, F: 0.09	phr	M: 0, F: 25	Absence of body contact, stick contact, ball contact, or unintended collision
Pasanen et al. [66]	2018	Floorball	A	3	M/F	-	ADO	M: 0, F: 8	M: 0.00, F: 0.32	phr	M: 0, F: 100	Absence of direct contact to injured body region or contact with other body parts
Rekik et al. [67]	2018	Soccer	E	5	M	–	AD	19	0.04	phr	51	Absence of direct contact (contact with knee) or indirect contact (contact with another body part)
Scranton Jr et al. [68]	1997	Am football	E	5	M	–	AD	61	0.07	pex	–	–
Senisik et al. [69]	2011	Soccer	I	2.5	M	64	AD	11	0.29	pex	100	–
Taylor et al. [40]	2011	Union	E	1	F	285	AD	1	0.91	phr	–	Injuries not as a result from contact with another player or object
Tondelli et al.[76]	2021	Union	A	1	M	250	AD	6	0.13	phr	67	–
Walden et al. [71]	2011	Soccer	E	3	M/F	M: 2019, F: 310	AD	M: 39, F: 6	M: 0.04, F: 0.06	phr	62	Absence of any physical contact with another player or object at the time of injury
Walden et al. [70]	2013	Soccer	E	9	M	1357	AD	31	0.04	phr	63	Injury resulting without player contact
Webb et al. [72]	2014	Lacrosse	E	1	M	–	–	0	0.00	phr	–	–
West et al. [73]	2020	Union	E	16	M	–	AD	19	C: 0.15^b^	phr	–	–
West et al. [38]	2020	Union	E	11	M	–	AD	4	T: < 0.01^b^	phr	–	–
Whalan et al. [74]	2019	Soccer	I	1	M	–	AD	5	0.10	phr	63	An injury that occurred without any contact to the player or the injury site by another player or object (ball, ground or equipment)
Willigenburg et al. [75]	2016	Am football, union	I	3	M	–	AD	Am football: 6 Union: 0	Am football: 0.21 Union: 0.00	pex	100	Injuries that did not follow from a direct hit to the affected area

*E* elite-level, *I* intermediate-level, *A* amateur-level, *M* male, *F* female, *AD* adult, *ADO* adolescent, *pex* per 1000 player-exposures, *phr* per 1000 player-hours, *C* competition, *T* training, *Au football* Australian football, *Am football* American football, *Gaelic* Gaelic football, *Union* Rugby union

^a^Combined training and competition for all meta-analyses except for meta-analyses by exposure type

^b^Only adolescent (under 18 years) data were extracted; data obtained from authors

**Table 2:** Quality assessment using a modified Newcastle-Ottawa scale

Study	Items					
	1	2	3	4	5	6
Agel et al. [47]	−	+	+	+	+	+
Agel et al. [45]	−	+	+	+	+	+
Anderson et al. [48]	−	+	+	+	−	+
Brooks et al. [37]	−	+	+	+	+	+
Dallalana et al. [49]	−	+	+	+	+	+
Dick et al. [46]	−	+	+	+	+	+
Donmez et al. [50]	+	+	+	−	−	−
Faude et al. [51]	+	+	+	+	+	+
Fuller et al. [43, 44]	+	+	+	+	+	+
Fuller et al. [39]	+	+	+	+	+	+
Fuller et al. [36]	+	+	+	+	+	+
Fuller et al. [35]	+	+	+	+	+	+
Fuller et al. [34]	+	+	+	+	+	+
Fuller et al. [42]	+	+	+	+	+	+
Fuller et al. [33]	+	+	+	+	+	+
Fuller et al. [41]	+	+	+	+	+	+
Giza et al. [52]	−	+	+	+	−	+
Gupta et al. [53]	−	+	+	+	+	+
Hartmut et al. [54]	+	+	+	+	++	+
Hollander et al. [55]	+	+	+	+	+	+
Joseph et al. [56]	−	+	+	+	+	+
Krutsch et al. [57]	+	+	+	+	−	+
Leppanen et al. [58]	+	+	+	+	+	+
Leyes et al. [59]	+	+	+	+	+	+
Loughran et al. [60]	−	+	+	+	+	+
Nilstad et al. [61]	+	+	+	+	−	+
O’Connor et al. [77]	−	+	+	+	−	+
Orchard et al. [62]	+	+	+	+	+	+
Ostenberg et al. [63]	+	+	+	+	−	+
Pasanen et al. [64]	+	+	+	+	−	+
Pasanen et al. [65]	−	+	+	+	+	−
Pasanen et al. [66]	+	+	+	−	+	+
Rekik et al. [67]	−	+	+	+	+	+
Scranton Jr et al. [68]	−	+	+	+	−	+
Senisik et al. [69]	+	−	−	+	−	−
Taylor et al. [40]	+	+	+	−	+	+
Tondelli et al. [76]	+	+	+	+	−	+
Walden et al. [71]	+	+	+	+	+	+
Walden et al. [70]	−	+	+	+	+	+
Webb et al. [72]	+	+	+	+	−	+
West et al. [73]	−	+	+	+	−	+
West et al. [38]	−	+	+	+	−	+
Whalan et al. [74]	+	+	+	+	+	+
Willigenburg et al. [75]	−	−	+	+	+	+

+ one star awarded; − no star awarded; Item 1 (population description): 1 star was awarded when the population at risk was fully described in terms of number, competition level, sex, age; Item 2 (Population recruitment): 1 star was awarded when it was described how the population under study was arrived at, and when the entire population participated, or a random sampling (fraction) method was used to follow a sample of the population at risk for non-contact knee injuries; Item 3 (Surveillance methods): 1 star was awarded when it was stated how the incidence of non-contact knee injuries were surveilled; Item 4 (Duration of observation): 1 star was awarded when the duration of observation was stated. If duration of observation was less than 1 season, duration in terms of days/weeks/months should be provided; if not, no star was awarded, Item 5 (Case definition): 1 star was awarded when the study defined both injury and injury mechanisms; Item 6 (Others): 1 star was awarded when all other methods were found appropriate

### Description of Included Studies

A total of 2,748 non-contact ACL injuries were recorded across 45 million player-hours and player-exposures combined (5 million player-hours and 40 million player-exposures) from 13 sports (soccer, American Football, rugby union, Australian football, Gaelic football, basketball, netball, volleyball, field hockey, floorball, lacrosse, baseball, softball) (Table 1). Most studies defined injuries based on the “time-loss” definition (89%) [11], and injury data were primarily collected and recorded by medical staff (91%). In studies that defined non-contact injury mechanisms (68%), some seemed to consider non-contact and indirect contact mechanisms together [58, 62, 75], while the rest defined the non-contact mechanism as no apparent player-player, surface-player, and ball-player contact.

### Study Quality Assessment

Initial agreement between reviewers was 80% (212 of 264 items), but all disagreements were subsequently resolved by consensus. Seventeen studies were awarded the maximum six stars and one study scored two stars (Table 2) [69]. Stars were most often not awarded because the population was not fully described (item (a): 41% awarded no stars), and because non-contact injury mechanisms were not defined (item (e): 32%).

### Publication Bias

Visual inspection of the funnel plots indicated that almost all studies in the meta-analyses had low standard errors, possibly due to large cohort sizes (Appendix A3). Studies were missing from the lower right quadrant in the funnel plot to assess publication bias in studies included in meta-analyses of overall proportion of non-contact to total ACL injuries, and this quadrant represents smaller studies with a high proportion of non-contact to total ACL injuries. Studies were evenly distributed in the funnel plot to assess publication bias in studies included in meta-analyses of overall incidence of non-contact ACL injuries.

### Proportion of Non-contact Anterior Cruciate Ligament (ACL) Injuries to Total ACL Injuries

**Fig. 2:**
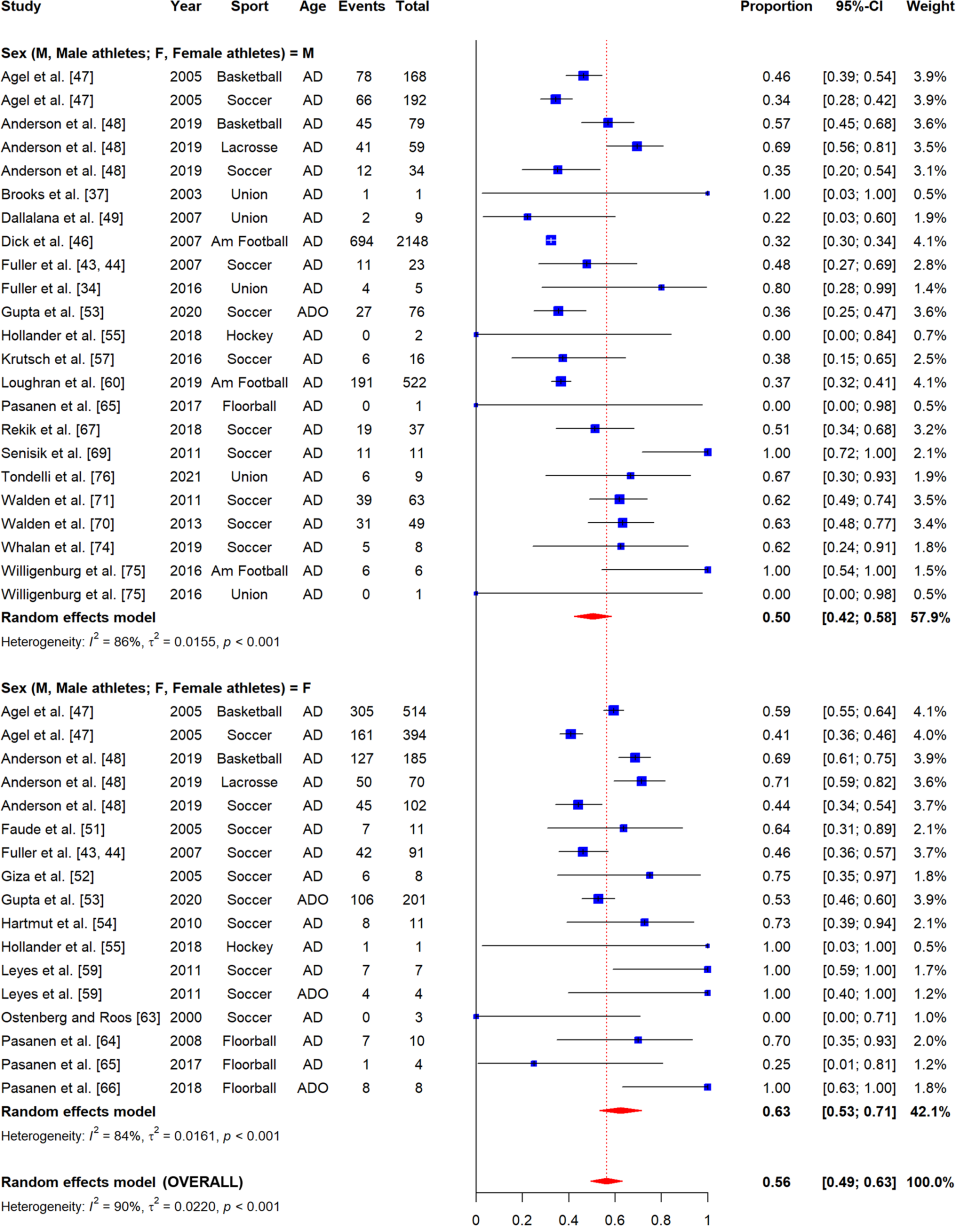
Forest plot of meta-analysis of proportion of ACL injuries sustained by non-contact mechanisms by sex. AD, adults; ADO, adolescents; Am Football, American Football; Au Football, Australian Football; Union, Rugby union; blue square, point estimate; red diamond, combined point estimate and 95% confidence intervals

The overall proportion of non-contact ACL injuries to total ACL injuries was 55% (95% CI 48–62, *I*^2^ = 82%) (Fig. 2).

#### By Sex

Non-contact ACL injury proportion was higher among female athlete (63%, 95% CI 53–71, *I*^2^ = 84%) compared to male athletes (50%, 95% CI 42–58, *I*^2^ = 86%) (Fig. 2).

#### By Sport

The overall proportion of non-contact ACL injuries to total ACL injuries was 66% in floorball (95% CI 15–100, *I*^2^ = 73%), 58% in basketball (95% CI 49–67, *I*^2^ = 84%), 54% in rugby union (95% CI 18–88, *I*^2^ = 42%), 53% in soccer (95% CI 46–61, *I*^2^ = 78%), and 38% in American football (95% CI 28–48, *I*^2^ = 89%) (Appendix A4-1). We were unable to perform meta-analyses for the other sports because there were less than three included studies (Appendix A4-1). Only the difference between field hockey and American football was significant, as confirmed by meta-regression (*β* = 0.29, 95% CI 0.03–0.55, *p* = 0.03) (Appendix B1). There were sufficient studies to sub-group by sport and sex for soccer only. In female soccer athletes, the proportion of non-contact to total ACL injuries was 55% (95% CI 45–65, *I*^2^ = 76%) (Appendix A4-2).

#### By Age Group

The overall proportion of non-contact ACL injuries to total ACL injuries was 55% in adults (95% CI 48–63, *I*^2^ = 90%) and 68% in adolescents (95% CI 43–90, *I*^2^ = 88%) (Appendix A5-1). After sub-grouping by age group and sex, this proportion was 60% in adult female athletes (95% CI 49–70, *I*^2^ = 85%) and 52% in adult male athletes (95% CI 43–60, *I*^2^ = 86%) (Appendix A5-2). There were insufficient studies to investigate injury proportions by sex in adolescents (Appendix A5-3) [53, 59, 66]. None of the included studies investigated children.

#### By Participation Level

The overall proportion of non-contact to total ACL injuries by participation level was 61% in elite-level (95% CI 50–70, *I*^2^ = 16%), 55% in intermediate-level (95% CI 44–65, *I*^2^ = 93%), and 56% in amateur-level athletes (95% CI 45–767, *I*^2^ = 89%) (Appendix A6-1). After sub-grouping by participation level and sex, this proportion was 65% in elite-level female athletes (95% CI 47–70, *I*^2^ = 0%), and 59% in elite-level male athletes (95% CI 45–72, *I*^2^ = 31%) (Appendix A6-2). In intermediate-level athletes, the proportion of non-contact to total ACL injuries in females was 58% (95% CI 43–73, *I*^2^ = 89%), and in males was 50% (95% CI 36–64, *I*^2^ = 88%) (Appendix A6-3). In amateur-level athletes, this proportion was 67% in females (95% CI 52–81, *I*^2^ = 85%), and 48% in males (95% CI 35–60, *I*^2^ = 82%) (Appendix A6-4).

#### By Exposure Type

The overall proportion of non-contact to total ACL injuries by exposure type was 42% (95% CI 30–54, *I*^2^ = 92%) in competition and 47% in training settings (95% CI 29–64, *I*^2^ = 72%) (Appendix A7-1). After sub-grouping by exposure type and sex, the proportion of non-contact to total ACL injuries during competition in female athletes was 58% (95% CI 42–74, *I*^2^ = 79%), and 35% in male athletes (95% CI 23–48, *I*^2^ = 87%) (Appendix A7-2). This difference between females and male athletes was significant as confirmed by meta-regression (*β* = − 0.22, 95% CI − 0.42 to − 0.02, *p* = 0.02) (Appendix B2). In training settings, this proportion was 68% in female athletes (95% CI 0.34–0.95, *I*^2^ = 60%) and 36% in male athletes (95% CI 21–53, *I*^2^ = 54%) (Appendix A7-3).

### Incidence of Non-contact ACL Injuries

**Fig. 3:**
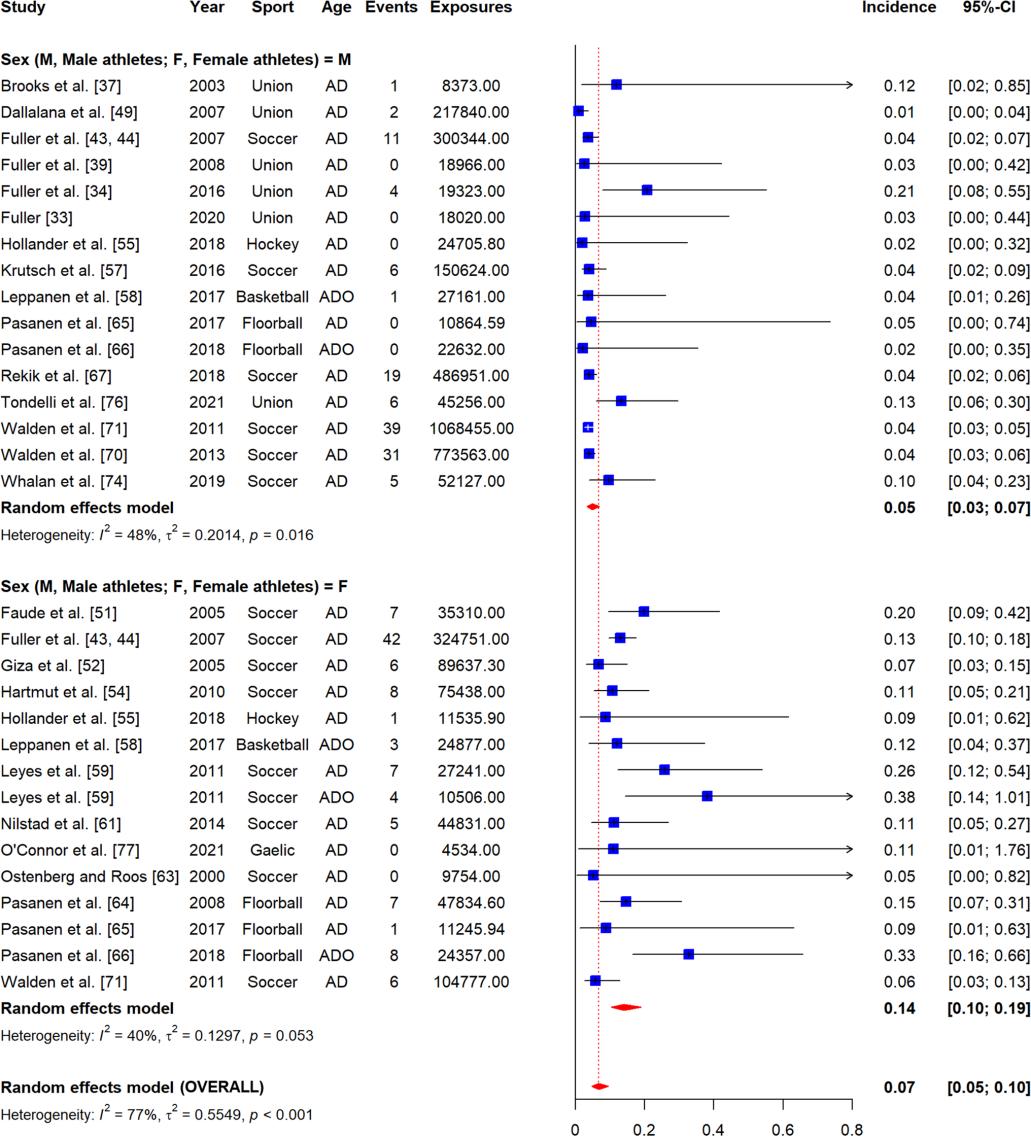
Forest plot of meta-analysis of incidence of non-contact ACL injuries per 1000 player-hours by sex. AD, adults; ADO, adolescents; Am Football, American Football; Au Football, Australian Football; Union, Rugby union; blue square, point estimate; red diamond, combined point estimate and 95% confidence intervals

**Fig. 4:**
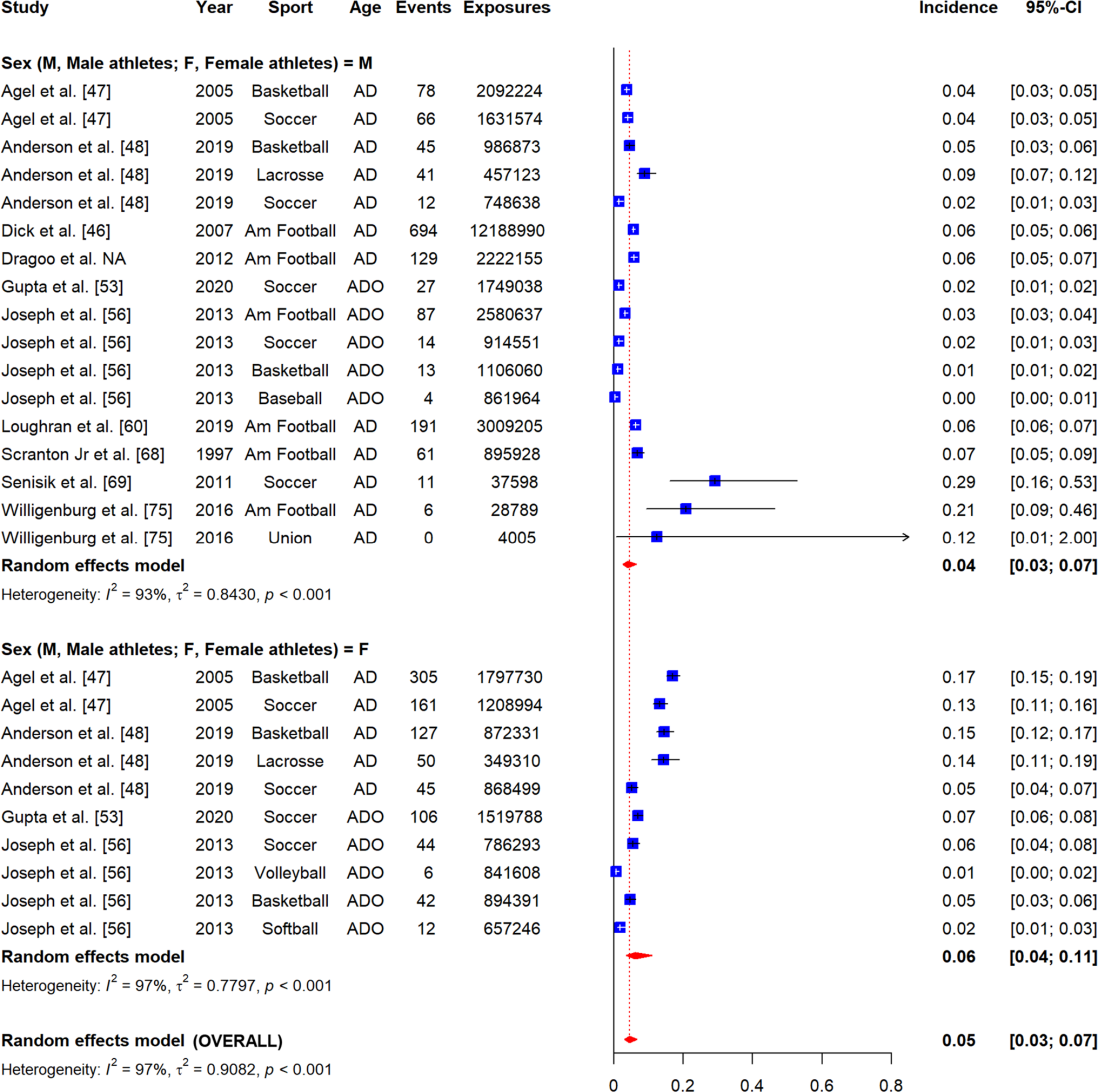
Forest plot of meta-analysis of incidence of non-contact ACL injuries per 1000 player-exposures by sex. AD, adults; ADO, adolescents; Am Football, American Football; Au Football, Australian Football; Union, Rugby union; blue square, point estimate; red diamond, combined point estimate and 95% confidence intervals

**Fig. 5:**
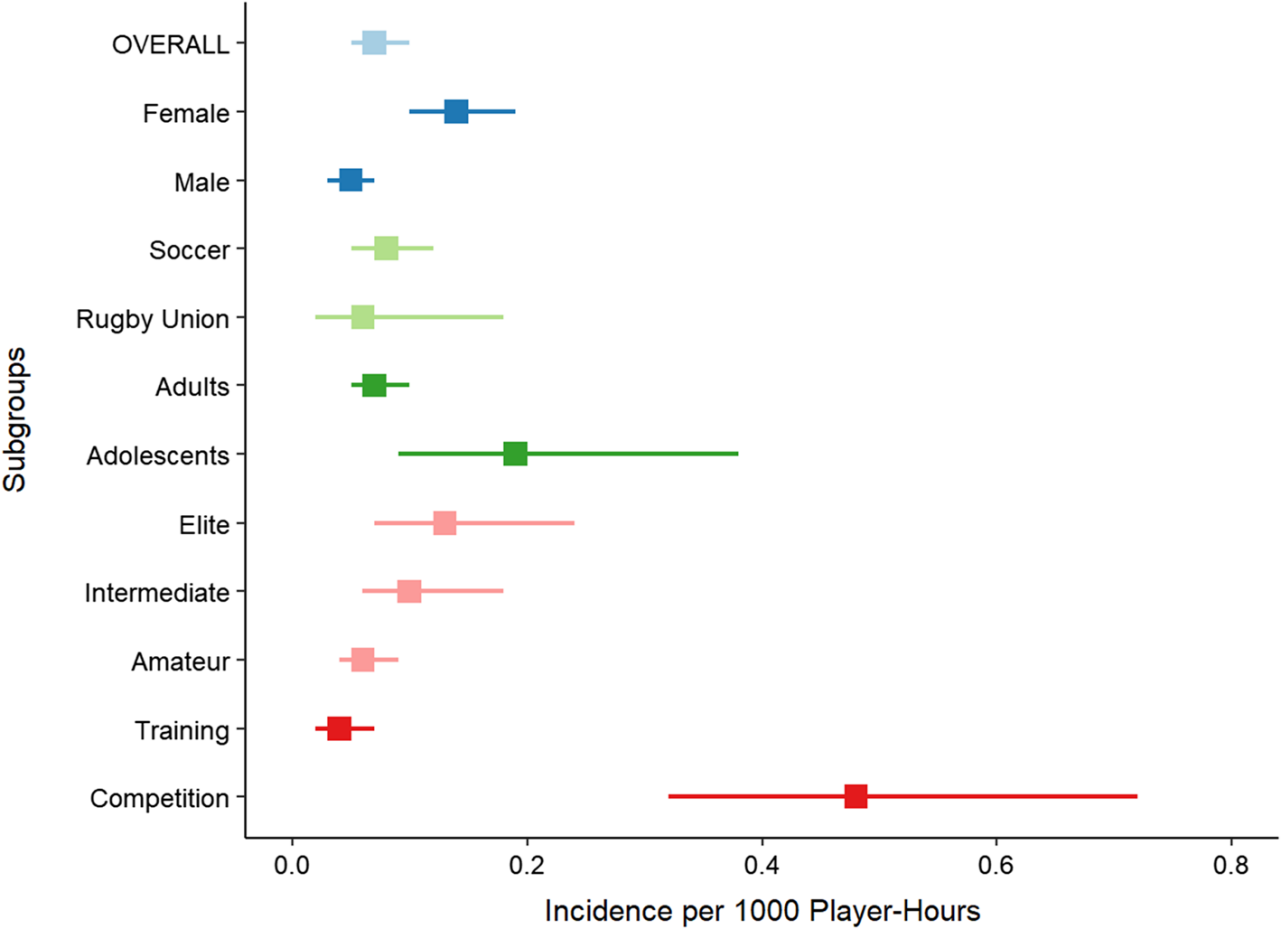
Summary of selected injury incidence meta-analyses by player-hours. Squares, summary measure; accompanying horizontal line, 95% CI

The overall incidence of non-contact ACL injuries was 0.07 per 1000 player-hours (95% CI 0.05–0.10, *I*^2^ = 77%) (Fig. 3), and 0.05 per 1000 player-exposures (95% CI 0.03–0.07, *I*^2^ = 97%) (Fig. 4). Figure 5 displays a summary of injury incidence meta-analyses by player-hours.

#### By Sex

In females, non-contact ACL injury incidence was 0.14 per 1000 player-hours (95% CI 0.10–0.19, *I*^2^ = 40%) and 0.06 per 1000 player-exposures (95% CI 0.04–0.11, *I*^2^ = 97%) (Figs. 3, 4). In males, injury incidence was 0.05 per 1000 player-hours (95% CI 0.03–0.07, *I*^2^ = 48%) and 0.04 per 1000 player-exposures (95% CI 0.03–0.07, *I*^2^ = 93%). Only the difference between female and male athletes per 1000 player-hours was significant as confirmed by meta-regression (*β* = − 1.15, 95% CI − 1.58 to − 0.73, *p* < 0.01) (Appendix B2).

#### By Sport

Overall, non-contact injury incidence was 0.06 per 1000 player-hours (95% CI 0.02–0.18, *I*^2^ = 68%) in rugby union, 0.08 per 1000 player-hours (95% CI 0.05–0.12, *I*^2^ = 84%) and 0.05 per 1000 player-exposures (95% CI 0.03–0.9, *I*^2^ = 97%) in soccer, 0.17 per 1000 player-hours in floorball (95% CI 0.09–0.32, *I*^2^ = 41%), 0.05 per 1000 player-exposures in basketball (95% CI 0.03–0.11, *I*^2^ = 98%), and 0.06 per 1000 player-exposures in American football (95% CI 0.05–0.08, *I*^2^ = 87%) (Appendices A8-1 and 8-2). There were insufficient studies for field hockey [55], Australian football [62], rugby sevens [41], lacrosse [48], volleyball [56], baseball [56], softball [56] and Gaelic football [77] (Table 1).

Sub-grouping by sport and sex was only possible for soccer and basketball. In soccer, injury incidence was higher in female athletes (0.13 per 1000 player-hours, 95% CI 0.09–0.19, *I*^2^ = 52%; 0.07 per 1000 player-exposures, 95% CI 0.05–0.11, *I*^2^ = 95%) compared to male athletes (0.04 per 1000 player-hours, 95% CI 0.03–0.05, *I*^2^ = 0%; 0.03 per 1000 player-exposures, 95% CI 0.01–0.09, *I*^2^ = 95%) (Appendices A8-3 and 8-4). Only the difference between female and male soccer athletes per 1000 player-hours was significant as confirmed by meta-regression (*β* = − 1.09, 95% CI − 1.38 to − 0.81, *p* < 0.01) (Appendix B2). Injury incidence was higher in female basketball players (0.11 per 1000 player-exposures, 95% CI 0.06–0.20, *I*^2^ = 97%) compared to males (0.03 per 1000 player-exposures, 95% CI 0.02–0.05, *I*^2^ = 89%) and this difference was significant (*β* = − 1.34, 95% CI − 2.25 to − 0.43, *p* < 0.01) (Appendices A8-5 and B2).

#### By Age Group

The overall incidence of non-contact ACL injuries in adults was 0.07 per 1000 player-hours (95% CI 0.05–0.10, *I*^2^ = 75%) and 0.08 per 1000 player-exposures (95% CI 0.06–0.11, *I*^2^ = 97%) (Appendices A9-1 and 9-2). In adolescents, the incidence was 0.19 per 1000 player-hours (95% CI 0.09–0.38, *I*^2^ = 57%) and 0.02 per 1000 player-exposures (95% CI 0.01–0.04, *I*^2^ = 94%) (Appendices 9-1 and 9-2). Only the difference between adults and adolescents per 1000 player-exposures was significant as confirmed by meta-regression (*β* = − 1.28, 95% CI − 1.87 to − 0.69, *p* < 0.01) (Appendix B2).

After sub-grouping by age group and sex, adult injury incidence was 0.11 per 1000 player-hours (95% CI 0.04–0.09, *I*^2^ = 74%) and 0.08 per 1000 player-exposures in female athletes (95% CI 0.05–0.11, *I*^2^ = 93%), and 0.05 per 1000 player-hours (95% CI 0.03–0.07, *I*^2^ = 55%) and 0.06 per 1000 player-exposures (95% CI 0.04–0.09, *I*^2^ = 89%) in male athletes (Appendices A9-3 and 9–4). The differences between adult male and female athletes per 1000 player-hours and player-exposures were significant as confirmed by meta-regression (*β* = − 1.08, 95% CI − 1.35 to − 0.81, *p* < 0.01 and *β* = − 0.69, 95% CI − 1.29 to − 0.10, *p* = 0.02, respectively) (Appendix B2). In adolescents, injury incidence was 0.02 per 1000 player-exposures in male athletes (95% CI 0.01–0.02, *I*^2^ = 89%), and 0.28 per 1000 player-hours (95% CI 0.17–0.46, *I*^2^ = 26%) and 0.03 per 1000 player-exposures for female athletes (95% CI 0.02–0.06, *I*^2^ = 91%) (Appendices A9-5 and A9-6). There were insufficient studies of male adolescent athletes to compare incidence per 1000 player-hours between adolescent male and female athletes [58, 66].

#### By Participation Level

Overall, the incidence of non-contact ACL injuries in elite-level athletes was 0.06 per 1000 player-hours (95% CI 0.04–0.09, *I*^2^ = 68%), in intermediate-level athletes 0.10 per 1000 player-hours (95% CI 0.06–0.18, *I*^2^ = 76%) and 0.10 per 1000 player-exposures (95% CI 0.06–0.17, *I*^2^ = 98%), and in amateur-level athletes 0.13 per 1000 player-hours (95% CI 0.07–0.24, *I*^2^ = 41%) and 0.03 per 1000 player-exposures (95% CI 0.02–0.05, *I*^2^ = 96%) (Appendices A10-1 and 10–2). There were insufficient studies of elite-level cohorts to compare incidence per 1000 player-exposures [68]. Only the difference between amateur- and intermediate-level athletes per 1000 player-exposures was significant as confirmed by meta-regression (*β* = 1.03, 95% CI 0.26–1.79, *p* < 0.01) (Appendix B2).

After sub-grouping by participation level and sex, the incidence of non-contact ACL injuries in elite-level female athletes was 0.10 per 1000 player-hours (95% CI 0.07–0.14, *I*^2^ = 19%) and in male athletes was 0.04 per 1000 player-hours (95% CI 0.03–0.05, *I*^2^ = 52%) (Appendix A10-3). There were insufficient studies to perform similar analyses per 1000 player-exposures [68]. The difference between male and female athletes per 1000 player-hours was significant as confirmed by meta-regression (*β* = − 0.91, 95% CI − 1.31 to − 0.52, *p* < 0.01) (Appendix B2). At the intermediate-level, injury incidence in females was 0.16 per 1000 player-hours (95% CI 0.12–0.21, *I*^2^ = 30%), and in male athletes was 0.04 per 1000 player-hours (95% CI 0.07–0.06, *I*^2^ = 0%) and 0.08 per 1000 player-exposures (95% CI 0.04–0.17, *I*^2^ = 92%) (Appendices A10-4 and A10-5). There were insufficient studies to meta-analyse data from female intermediate-level athletes [47]. Only the difference between male and female athletes per 1000 player-hours was significant as confirmed by meta-regression (*β* = − 1.32, 95% CI − 1.85 to − 0.80, *p* < 0.01) (Appendix B2). At the amateur-level, injury incidence in females was 0.27 per 1000 player-hours (95% CI 0.14–0.51, *I*^2^ = 0%) and 0.05 per 1000 player-exposures (95% CI 0.03–0.09, *I*^2^ = 95%), and in males was 0.10 per 1000 player-hours (95% CI 0.06–0.18, *I*^2^ = 0%) and 0.02 per 1000 player-exposures (95% CI 0.01–0.04, *I*^2^ = 95%) (Appendices A10-6 and A10-7).

#### By Exposure Type

The overall incidence of injury during competition was 0.48 per 1000 player-hours (95% CI 0.32–0.72, *I*^2^ = 77%) and 0.32 per 1000 player-exposures (95% CI 0.15–0.70, *I*^2^ = 96%), and during training was 0.04 per 1000 player-hours (95% CI 0.02–0.07, *I*^2^ = 63%) and 0.02 per 1000 player-exposures (95% CI 0.01–0.05, *I*^2^ = 86%) (Appendices A11-1 and A11-2).

After sub-grouping by exposure types and sex, injury incidence in female athletes during competition was 0.67 per 1000 player-hours (95% CI 0.33–1.35, *I*^2^ = 75%) and in training was 0.07 per 1000 player-hours (95% CI 0.05–0.10, *I*^2^ = 2%) (Appendix A11-3). There were insufficient studies to perform meta-analysis by player-exposures in females [45, 53]. For males, injury incidence was 0.37 per 1000 player-hours (95% CI 0.24–0.59, *I*^2^ = 72%) and 0.34 per 1000 player-exposures, 95% CI 0.12–0.99, *I*^2^ = 96%) during competition while for training was 0.02 per 1000 player-hours (95% CI 0.01–0.05, *I*^2^ = 55%) and 0.03 per 1000 player-exposures (95% CI 0.01–0.06, *I*^2^ = 83%). All competition to training comparisons reported above were significant as confirmed by meta-regression (Appendix B2).

#### Additional Sub-group Analyses

There were sufficient studies to perform a meta-analysis by participation level per 1000 player-hours for female soccer athletes. Injury incidence was higher in intermediate- (0.18 per 1000 player-hours, 95% CI 0.11–0.29, *I*^2^ = 57%) compared to elite-level athletes (0.10 per 1000 player-hours, 95% CI 0.07–0.15, *I*^2^ = 35%) (Appendix A12). This difference was significant as confirmed by meta-regression (*β* = 0.44, 95% CI < 0.01–0.88, *p* = 0.05) (Appendix B2).

## Discussion

We conducted a systematic review with meta-analysis to estimate the proportion of non-contact to total ACL injuries and describe the incidence of non-contact ACL injuries in team ball-sports. Compared to the two most recent systematic reviews on ACL injury epidemiology, our review captured more ACL injuries, estimated incidence according to player-hours and player-exposures, and performed meta-regression analyses to investigate sources of heterogeneity and to test the influence of sex, age group, sport, participation level and exposure type on effect sizes [1, 9]. Overall, we found that non-contact ACL injuries represented over half of all ACL injuries sustained in team ball-sports. Non-contact ACL injury proportion was higher in female than male athletes in team ball-sports. Injury incidence was higher in females than males with most injuries occurring during competition team ball-sports. Intermediate-level male and female athletes were more likely to sustain non-contact ACL injuries than amateur-level athletes in team ball-sports. Heterogeneity across studies was generally high.

While female athletes are at a greater risk of ACL injuries compared to male athletes [1, 9, 78], this is the first systematic review to confirm that a similar sex disparity also exists for non-contact ACL injury risk. There is no consensus from multi-pronged research investigating the sex disparity in ACL injury rates through anatomical [79], physiological [80] and biomechanical lenses [81, 82], and injury rates in females remain high [1–3, 9]. A recent review by Parsons et al. called for ACL injury risk-reduction research to consider the influence of societal [83] and cultural norms of female athletes [84]. Parsons and colleagues provided the example that it is not uncommon for girls to be told to ‘get stronger’ to reduce ACL injury risk, but are not provided with equal opportunity and support to do so [84]. There is a need for a holistic approach to address this injury rate disparity.

Consistent with previous research, athletes were more likely to sustain non-contact ACL injuries in competition than training settings [43, 44, 51, 71]. Competition settings are often associated with additional internal and external stressors, and failure to manage these stressors may increase injury risk [85]. Training sessions are usually conducted in a more controlled environment than competition settings; therefore, it should be easier to reduce non-contact ACL injuries in training [38]. To do so, it seems logical to employ strategies like technique instruction, optimizing workload, and exercise-based IRRPs. However, the purpose of training is to prepare athletes for the physical demands of sport, and a reduction in injury incidence that comes at the expense of team performance may not be acceptable to coaches. While the search continues for the elusive training “sweet-spot” to reduce injury risk while improving performance [86], stakeholders should consider cost-effectiveness analyses and systems thinking approaches to assess injury risk reduction opportunities and challenges, as these are usually unique to each sport and setting [8, 87].

Our findings were inconclusive regarding the influence of sport, age group and participation level on non-contact ACL injury epidemiology in team ball-sports. In relation to the influence of sport, Montalvo et al*.* previously reported the highest incidence of ACL injuries in high-impact rotational landing (gymnastics, obstacle course race) and contact sports (soccer, basketball) [1]. It is not clear, however, if these differences were significant because meta-regression analyses were not performed in that study. One possible explanation for the lack of difference in our findings could be due to the common non-contact ACL injury scenarios and mechanisms across team ball-sports [4, 18, 88, 89]. In relation to the influence of age group, studies have suggested that children and adolescents are more susceptible to injury compared to adults because of their lower skill levels, physical capacities, and decision-making capabilities [90–92]. We only found a significant difference in injury incidence when comparing adults to adolescents by player-exposure but not for incidence by player-hours or proportion. None of the included studies investigated children. Sub-grouping by sex did not reveal any significant findings. With respect to participation level, our findings mirror the current state of evidence that it is not clear if amateur- and intermediate-level athletes are more susceptible to injuries, as found in some cohorts [1, 9], or if elite-level athletes are more susceptible [27, 93]. We did find, however, that intermediate-level male and female athletes were more likely to sustain non-contact ACL injuries than amateur-level athletes. We should caution that our findings on the influence of sport, age group, and participation level were from meta-analyses with high heterogeneity, and further sub-group analyses requiring more studies may be needed to determine the influence of these categorical predictors on non-contact ACL injury epidemiology.

Lastly, in order to fully establish the extent of an injury problem to inform the development of injury risk-reduction strategies, injury epidemiology studies must report injury mechanisms [7]. We had to exclude nearly four times as many studies from our review than those included because they did not report whether the injuries occurred via a direct contact or non-contact mechanism (168 studies excluded vs. 44 included) (Fig. 1). Additionally, authors from 22 out of the 46 included studies had to be contacted because non-contact ACL injury data were not available in the published manuscript [33–44, 50, 51, 55, 65, 66, 73–77]. To illustrate the importance of reporting injury mechanisms, the Australian Football League introduced rule changes to limit the run-up of ruckmen at the centre bounce that reduced posterior cruciate ligament (PCL) injury risk by half [94]. They were successful in doing so because they had identified that PCL injuries commonly occurred through knee-to-knee contact mechanisms, and by limiting the run up, ruckmen had lower momentum and were not jumping and lifting their knees up as high during these contests. Without knowledge that most PCL injuries occurred through contact mechanisms, the proposed injury counter-measures would not have been as effective. Therefore, future studies on injury epidemiology should adopt consensus statement guidelines to not just report injury magnitude, but also injury mechanisms and their accompanying definitions [11].

### Limitations

Firstly, there was substantial heterogeneity among the included studies. This is inevitable in meta-analyses of epidemiological studies and does not invalidate our findings [95]. We attempted to investigate sources of heterogeneity via random-effects meta-analytical methods and sub-group analyses. Future research should explore potential sources of heterogeneity not investigated in our review. Next, previous knee and ACL injuries increase the risk of subsequent ACL injuries [96], but detailed information regarding medical history was not available in most included studies and therefore was not considered in this review. Subgrouping according to index versus re-injuries may improve the generalizability of our findings. Another limitation was that non-contact injury mechanisms were mostly defined in the included studies as the absence of direct player-to-player or player-to-surface contact (Table 1). However, these definitions were unclear on whether indirect contact mechanisms were considered. Indirect contact is defined as physical contact not applied directly to the knee, but contributes to the causal chain of events leading to an ACL injury [11]. For example, shoulder contact between soccer players jostling in mid-air for a header can result in an external perturbation of the centre of mass that affects knee landing kinematics and eventuates in an ACL rupture. Up to 44% of ACL injuries could be due to indirect contact mechanisms [4], and these injuries could arguably be prevented through careful drill design that replicates contact events in sport and instruction of proper technique [97]. The inclusion of ACL injuries sustained by indirect contact mechanisms would likely provide a more accurate estimate on the incidence and proportion of injuries that are amenable to exercise-based IRRPs. It is probable that ACL injury data might not be reported in studies where no ACL injuries occurred: these studies should report zero cases to prevent effect size overestimation. This review only included studies investigating team ball-sports and our results should not be generalized to all sports. Lastly, including non-published data might affect the validity and reproducibility of this review, so we used systematic and detailed criteria and processes to maintain transparency throughout this process.

## Conclusion

Non-contact ACL injuries represented over half of all ACL injuries sustained in team-ball sports. The proportion of non-contact to total ACL injuries and injury incidence was higher in females than males in team ball-sports. Injuries mostly occurred in competition settings in team ball-sports. More research is required to fully understand the influence of sport, age group and participation level on injury proportion and incidence in team ball-sports. Our findings have implications for future ACL epidemiological research in sport, and the development and implementation of effective ACL injury risk reduction efforts in team ball-sports.

## Supplementary Information

Below is the link to the electronic supplementary material.

Supplementary file1 (DOCX 4860 KB)

Supplementary file2 (DOCX 45 KB)

## Declarations

### Data availability

Raw data from data analysis are available upon reasonable request by contacting the corresponding author. The data are not publicly available because a portion of it is obtained directly from authors of the included studies, and subsequent approval from these authors is required.
