# Supplementary material for: Non-contact Anterior Cruciate Ligament Injury Epidemiology in Team-Ball Sports: A Systematic Review with Meta-analysis by Sex, Age, Sport, Participation Level, and Exposure Type
Source: Sports Med. 2022 May 27;52(10):2447–67. doi: 10.1007/s40279-022-01697-w (PMC9136558; doi:10.1007/s40279-022-01697-w)
Supplement: Supplementary file 1 — Supplementary file1 (DOCX 4860 KB) [file 40279_2022_1697_MOESM1_ESM.docx]

|  | **CONTENTS** |  |
| --- | --- | --- |
|  |  |  |
| A1 | Search Strategy (MEDLINE) ………...………………………………………………………... | 2 |
| A2 | Modified Newcastle-Ottawa scale ……………………………………………………………... | 3 |
| A3 | Funnel plots ……………………………………………………………………...……………... | 4 |
|  | **Forest plots of meta-analyses of proportion of non-contact to total ACL injuries** |  |
| A4 | By sport ……………….………………………...……………………………………...……...... | 5 |
| A5 | By age group .……...…...………………………………………………………...……………... | 8 |
| A6 | By participation level .……….....….………………………………………………….………… | 11 |
| A7 | By exposure type ………….………………………………………………………………......… | 14 |
|  | **Forest plots of meta-analyses of incidence of non-contact ACL injuries** |  |
| A8 | By sport ………….…………………………………….………………………………………... | 16 |
| A9 | By age group …………….……………………………………………………………………… | 21 |
| A10 | By participation level …………………………………………………………………………… | 26 |
| A11 | By exposure type …………….……………………………………….………………….……… | 31 |
| A12 | Additional sub-group analyses …………………………………………………………………. | 37 |
|  |  |  |
|  | **Legend**  ACL, anterior cruciate ligament; AD, adults; ADO, adolescents; Am football, American football; Au football, Australian football; M, male; F, female; C, competition setting; T, training setting |  |

**A1 SEARCH STRATEGY (MEDLINE)**

Database: Ovid MEDLINE(R) ALL <1946 to July 16, 2020>

Search Strategy:

--------------------------------------------------------------------------------

1 Sports/ or team-sport*.mp. or ball-sport*.mp. (32171)

2 soccer.mp. or Soccer/ (11366)

3 football.mp. or Football/ or rugby.mp. or gridiron.mp. (13362)

4 basketball.mp. or Basketball/ (4413)

5 netball.mp. (217)

6 hockey.mp. or Hockey/ (3033)

7 handball.mp. (1135)

8 volleyball.mp. or Volleyball/ (1893)

9 floorball.mp. (84)

10 lacrosse.mp. (443)

11 futsal.mp. (222)

12 hurling.mp. (101)

13 (baseball or softball).mp. or Baseball/ (3969)

14 1 or 2 or 3 or 4 or 5 or 6 or 7 or 8 or 9 or 10 or 11 or 12 or 13 (60328)

15 (lower limb* or lower extremit*).mp. or Lower Extremity/ (107303)

16 Knee/ or knee.mp. or Knee Injuries/ (166497)

17 Patellofemoral Joint/ or Patellofemoral Pain Syndrome/ or patellofemoral.mp. (6659)

18 "anterior cruciate ligament".mp. or Anterior Cruciate Ligament/ (21771)

19 Tibial Meniscus Injuries/ or Menisci, Tibial/ or menisc*.mp. (18484)

20 iliotibial band.mp. (859)

21 (tibi?femoral or tibi?-femoral).mp. (3404)

22 Medial Collateral Ligament, Knee/ or medial collateral ligament*.mp. or lateral collateral ligament.mp. (3344)

23 15 or 16 or 17 or 18 or 19 or 20 or 21 or 22 (272313)

24 injur*.mp. or "Wounds and Injuries"/ (1206121)

25 "Sprains and Strains"/ or Athletic Injuries/ or sprain*.mp. or strain*.mp. (1035487)

26 (tear* or pain* or tend?n* or syndrome).mp. (2132304)

27 24 or 25 or 26 (4109010)

28 incidence.mp. or Incidence/ (855352)

29 Prevalence/ or prevalence.mp. (710527)

30 Epidemiology/ or Epidemiologic Studies/ or epidemiolog*.mp. or Epidemiological Monitoring/ (1898259)

31 pattern*.mp. (1395469)

32 (burden or rate* or frequenc*).mp. (4050177)

33 28 or 29 or 30 or 31 or 32 (6935721)

34 14 and 23 and 27 and 33 (2323)

***************************

**A2 MODIFIED NEWCASTLE-OTTAWA SCALE**

| **Modified Newcastle-Ottawa scale** |
| --- |
| Item 1 (Population description): 1 star was awarded when the population at risk was fully described in terms of number, competition level, sex, age. |
| Item 2 (Population recruitment): 1 star was awarded when it was described how the population under study was arrived at, and when the entire population participated, or a random sampling (fraction) method was used to follow a sample of the population at risk for non-contact knee injuries |
| Item 3 (Surveillance methods): 1 star was awarded when it was stated how the incidence of non-contact knee injuries were surveilled (e.g. by medical staff, self-reported, non-medical staff, combination). |
| Item 4 (Duration of observation): 1 star was awarded when the duration of observation was stated. If duration of observation was less than 1 season, duration in terms of days/weeks/months should be provided; if not, no star was awarded. |
| Item 5 (Case definition): 1 star was awarded when the study defined both injury (e.g. in terms of time-loss, medical attention, all complaints, via MRI, other) and injury mechanisms (e.g. for non-contact mechanisms, studies should detail whether they defined non-contact to be absent of player-player contact, or otherwise). |
| Item 6 (Others): 1 star was awarded when all other methods were found appropriate. An example of an inappropriate method is estimation of exposure rather than objective recording and documentation. |


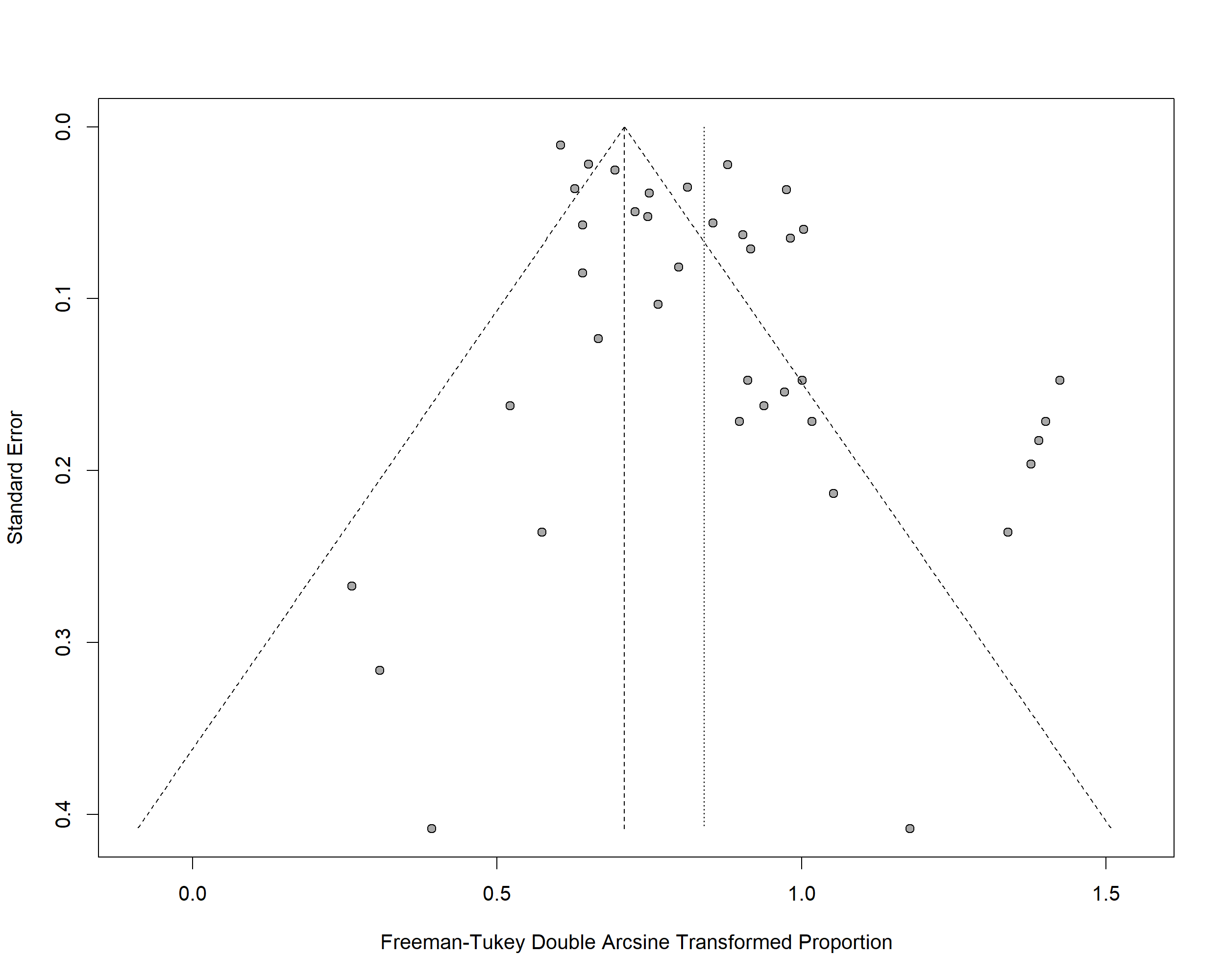
**A3 FUNNEL PLOTS**

**A3-1** Funnel plot to assess publication bias in studies included in meta-analyses of overall incidence of non-contact ACL injuries


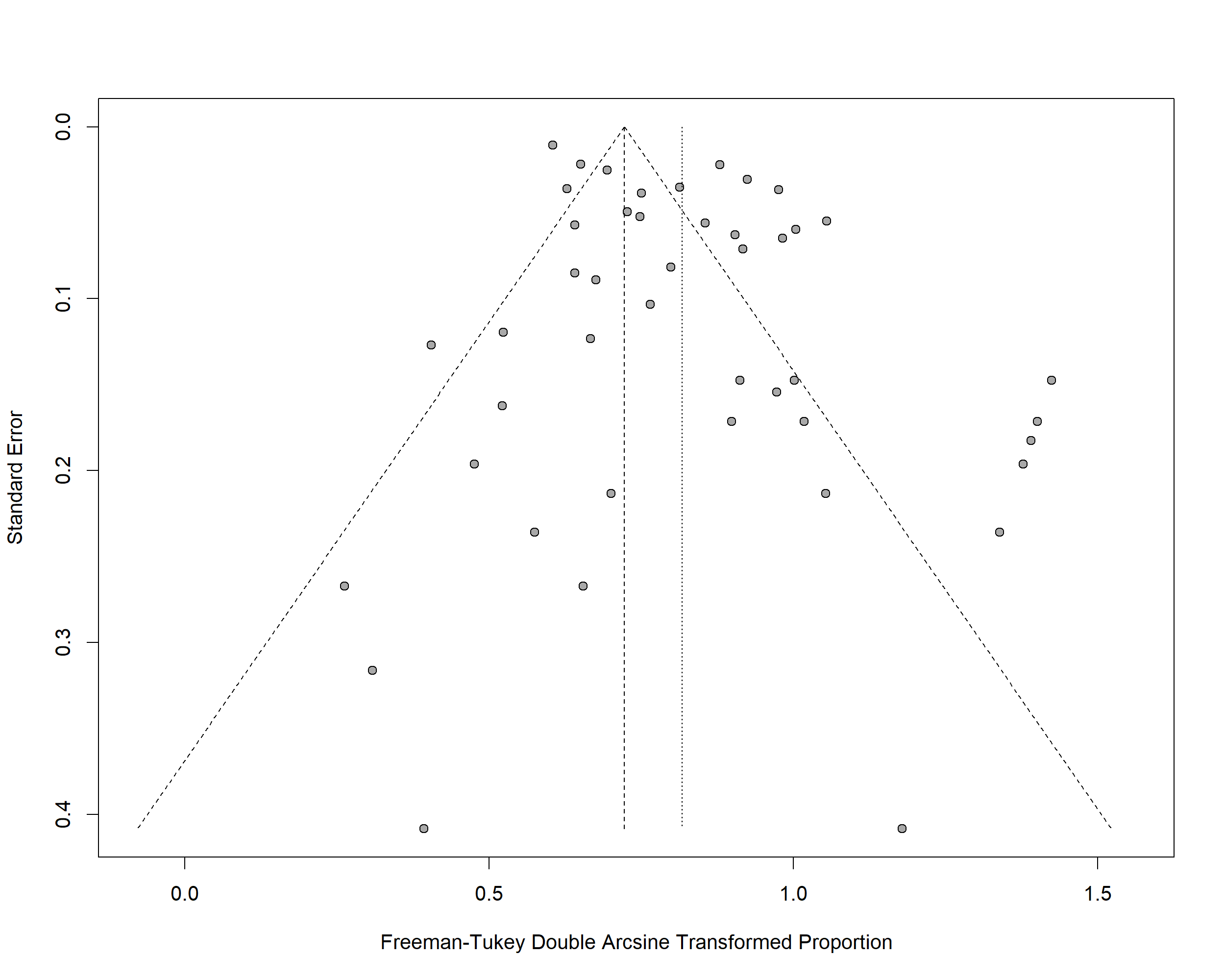


**A3-2** Funnel plot to assess publication bias in studies included in meta-analyses of overall proportion of non-contact to total ACL injuries

**A4 FOREST PLOTS OF META-ANALYSIS OF PROPORTION OF NON-CONTACT ACL INJURIES OUT OF TOTAL ACL INJURIES BY SPORT**


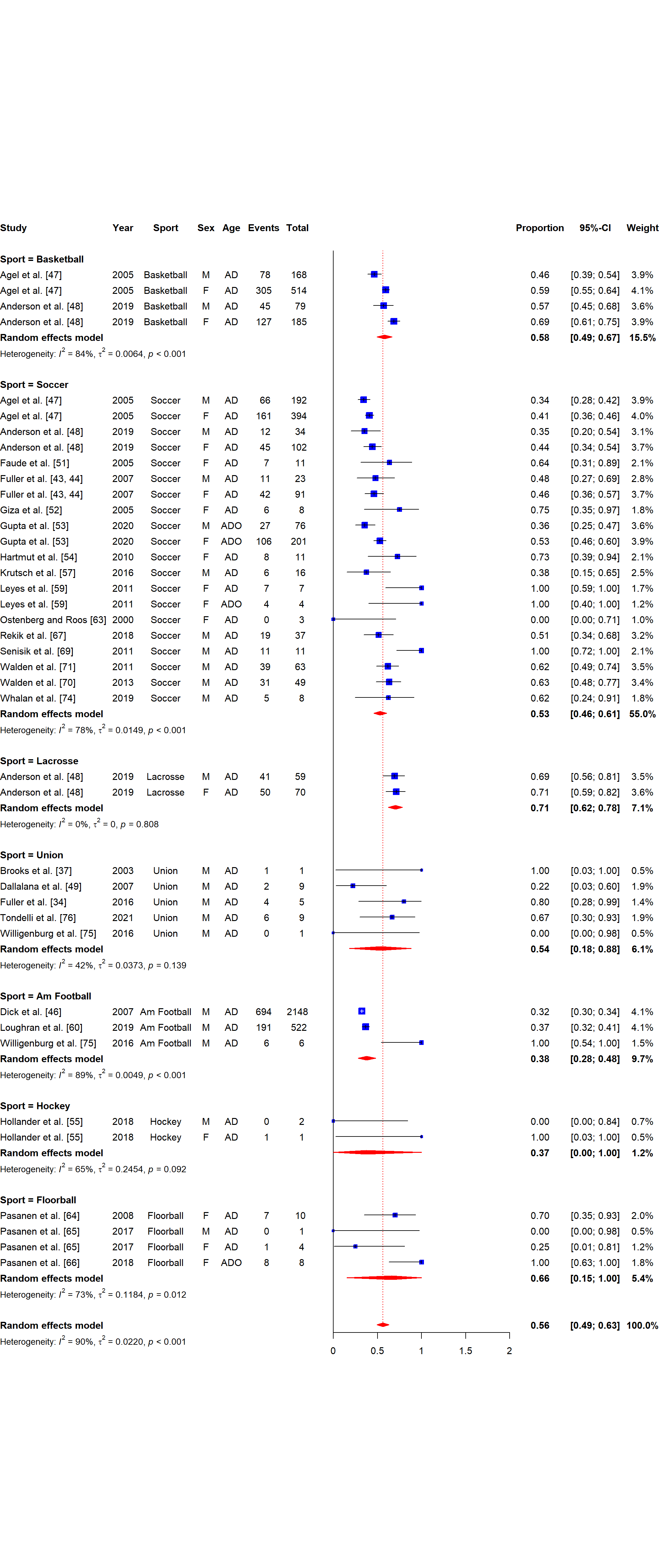


*Continued next page*


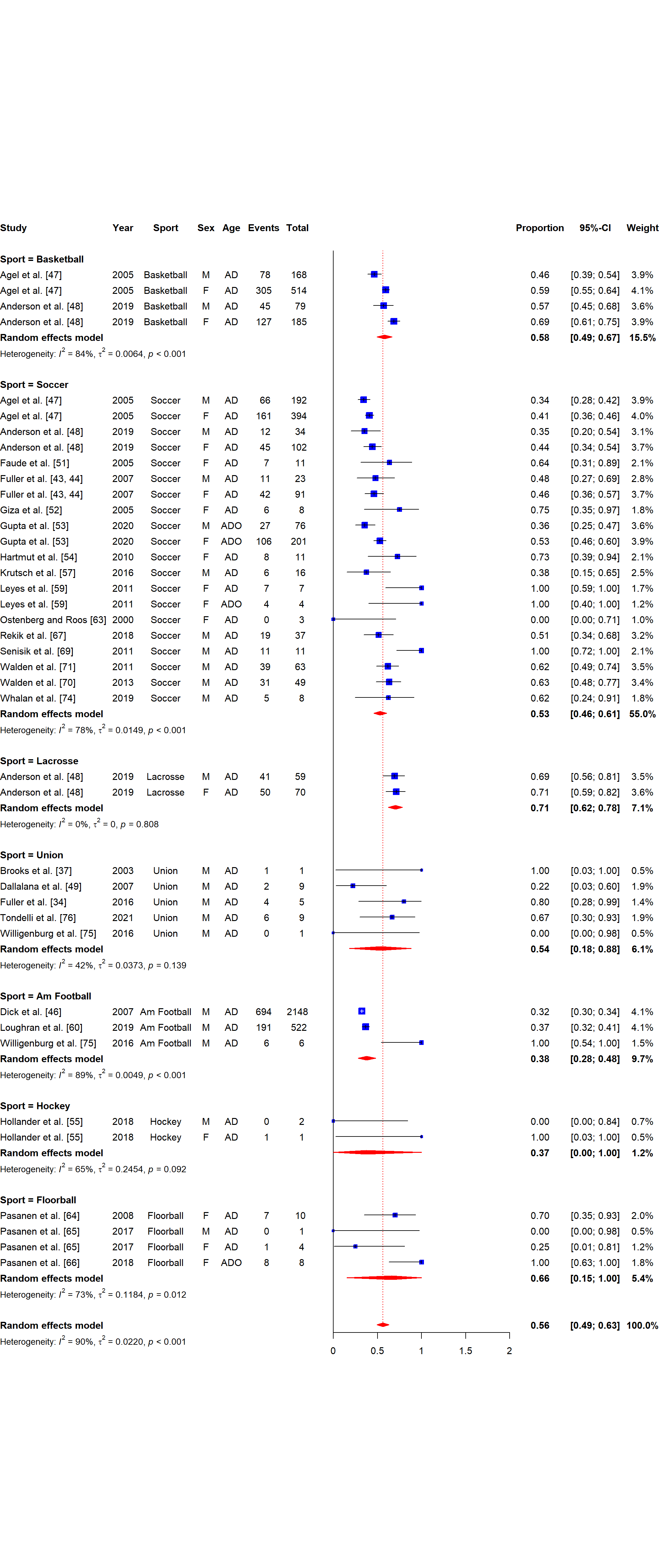


**A4-1** Forest plot of meta-analysis of proportion of non-contact ACL injuries to total ACL injuries by sport


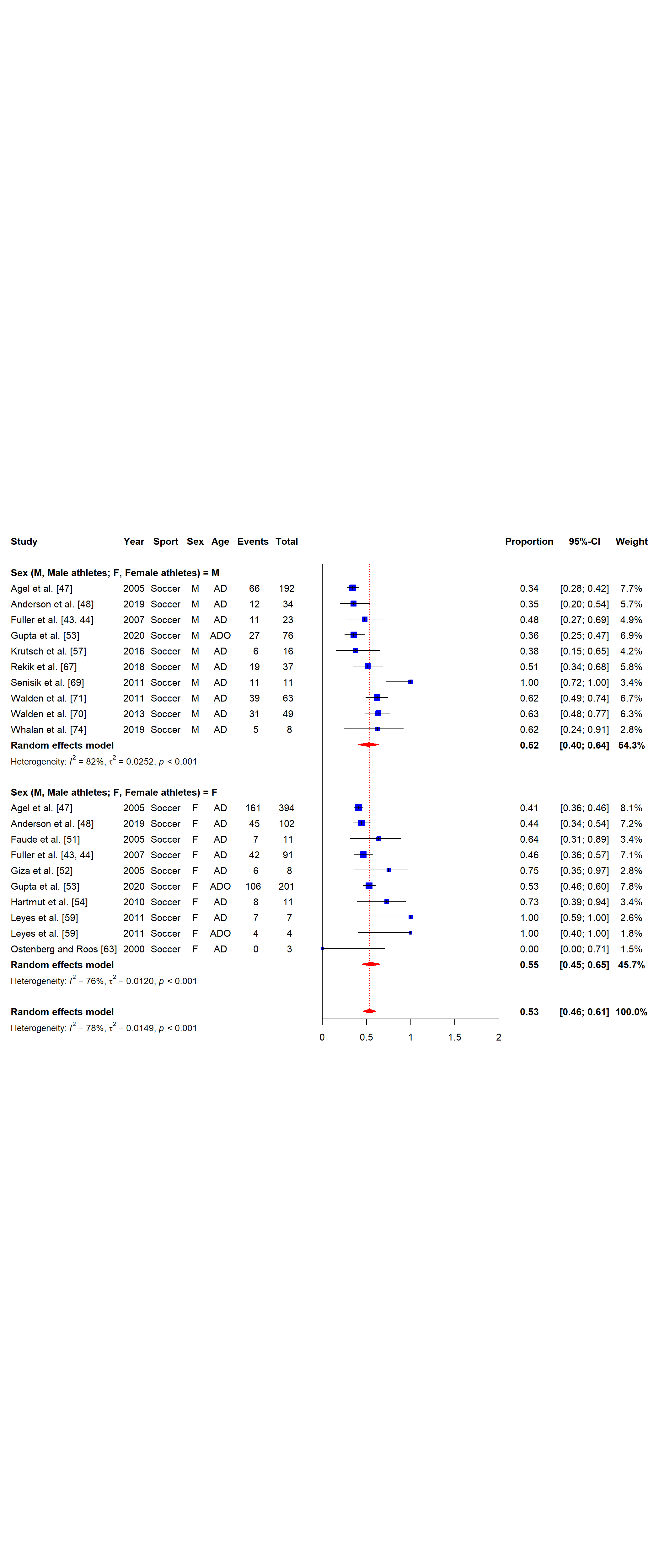


**A4-2** Forest plot of meta-analysis of proportion of non-contact ACL injuries to total ACL injuries in soccer by sex

**A5 FOREST PLOTS OF META-ANALYSIS OF PROPORTION OF NON-CONTACT ACL INJURIES OUT OF TOTAL ACL INJURIES BY AGE GROUP**


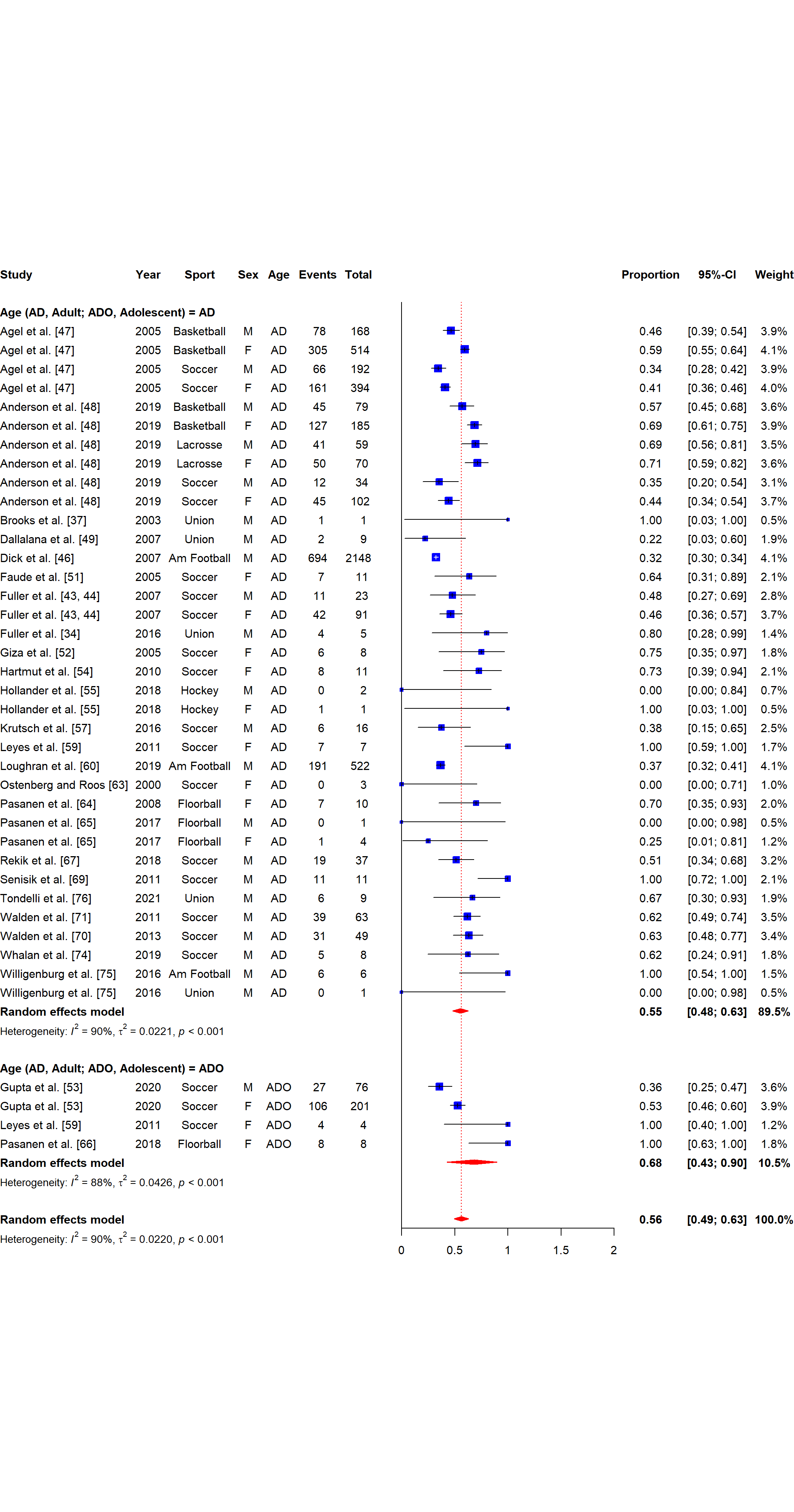


**A5-1** Forest plot of meta-analysis of proportion of non-contact ACL injuries to total ACL injuries by age


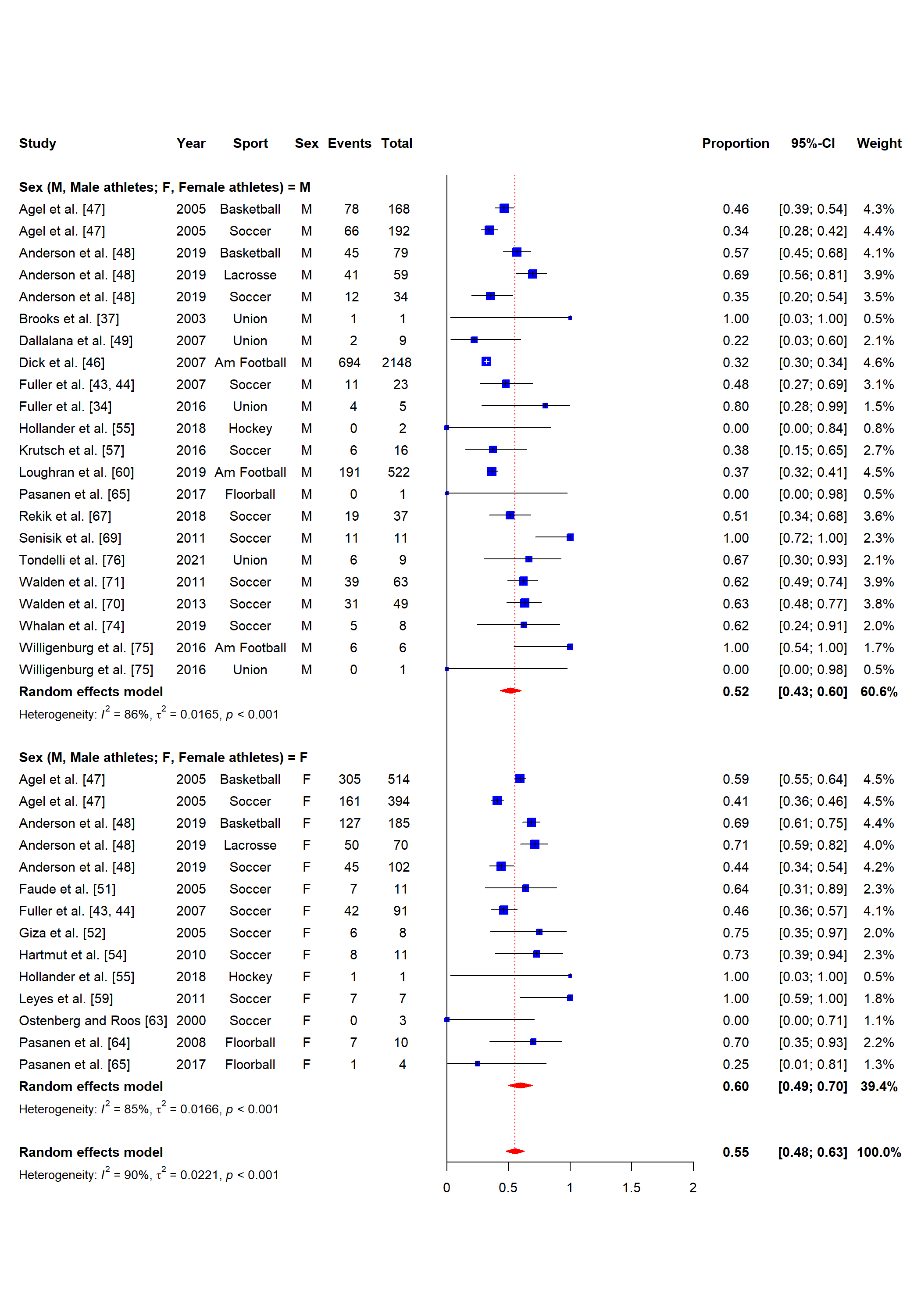


**A5-2** Forest plot of meta-analysis of proportion of non-contact ACL injuries to total ACL injuries in adults by sex


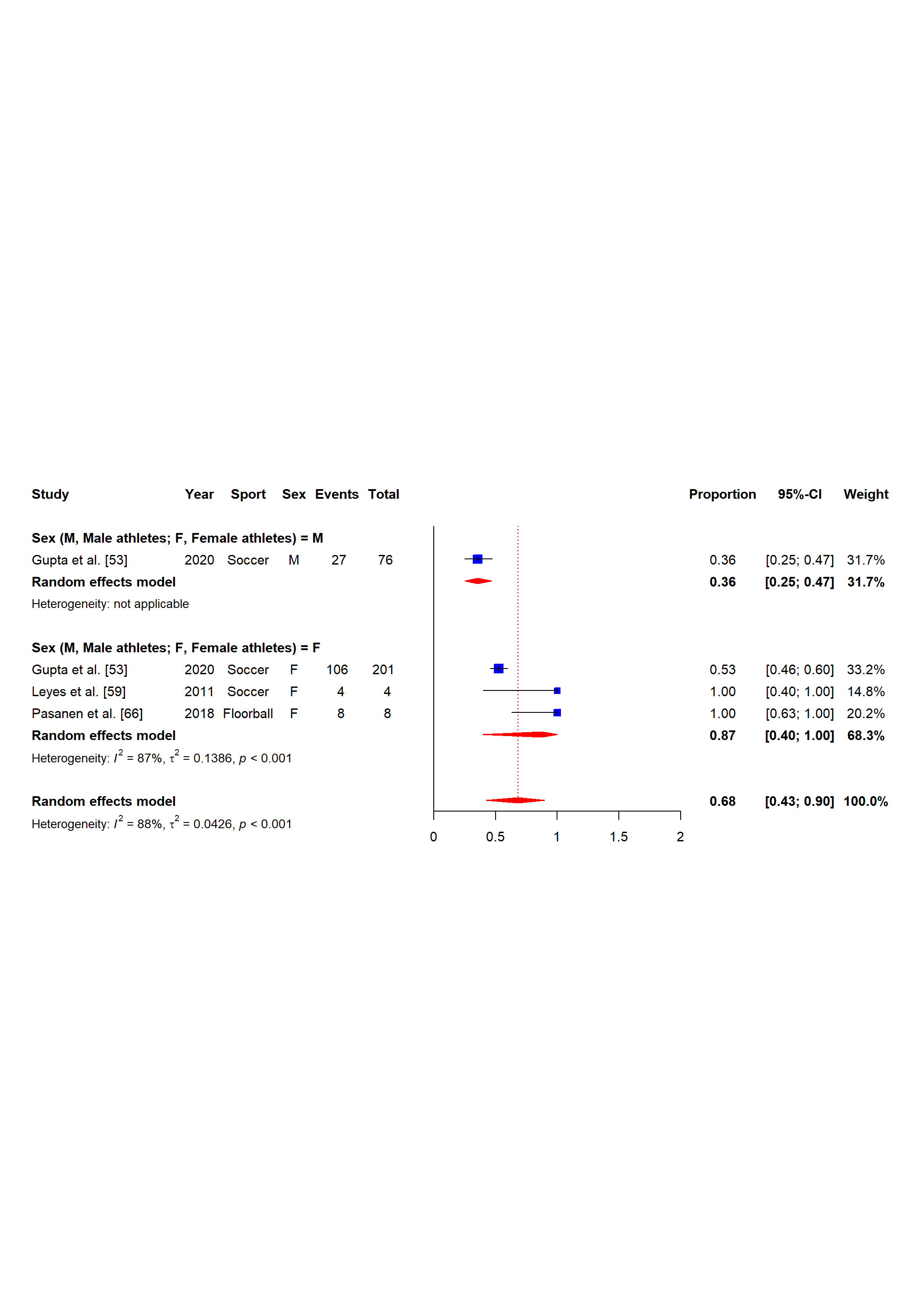


**A5-3** Forest plot of meta-analysis of proportion of non-contact ACL injuries to total ACL injuries in adolescents by sex

**A6 FOREST PLOTS OF META-ANALYSIS OF PROPORTION OF NON-CONTACT ACL INJURIES TO TOTAL ACL INJURIES BY PARTICIPATION LEVEL**


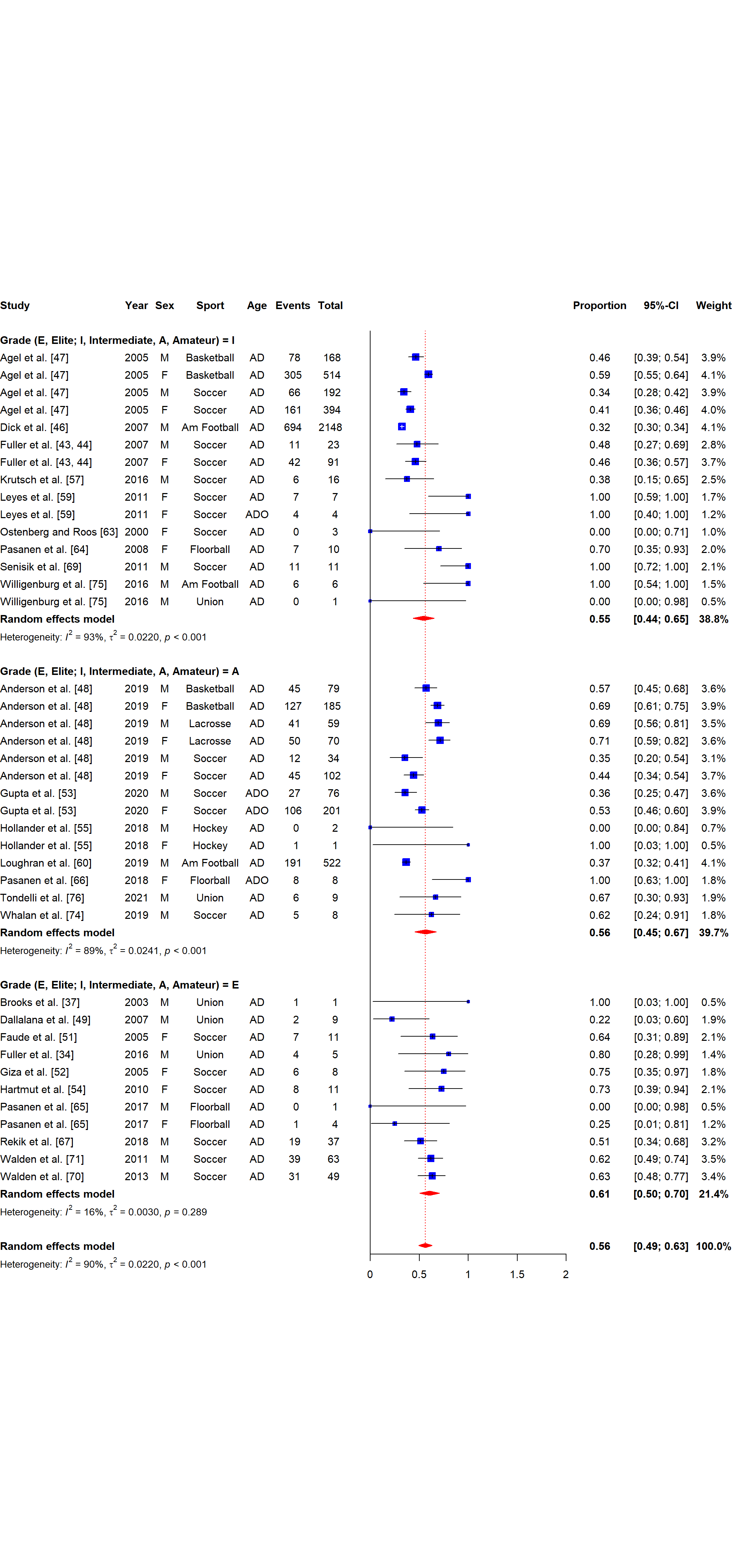


**Figure A6-1** Forest plot of meta-analysis of proportion of non-contact ACL injuries to total ACL injuries by participation level


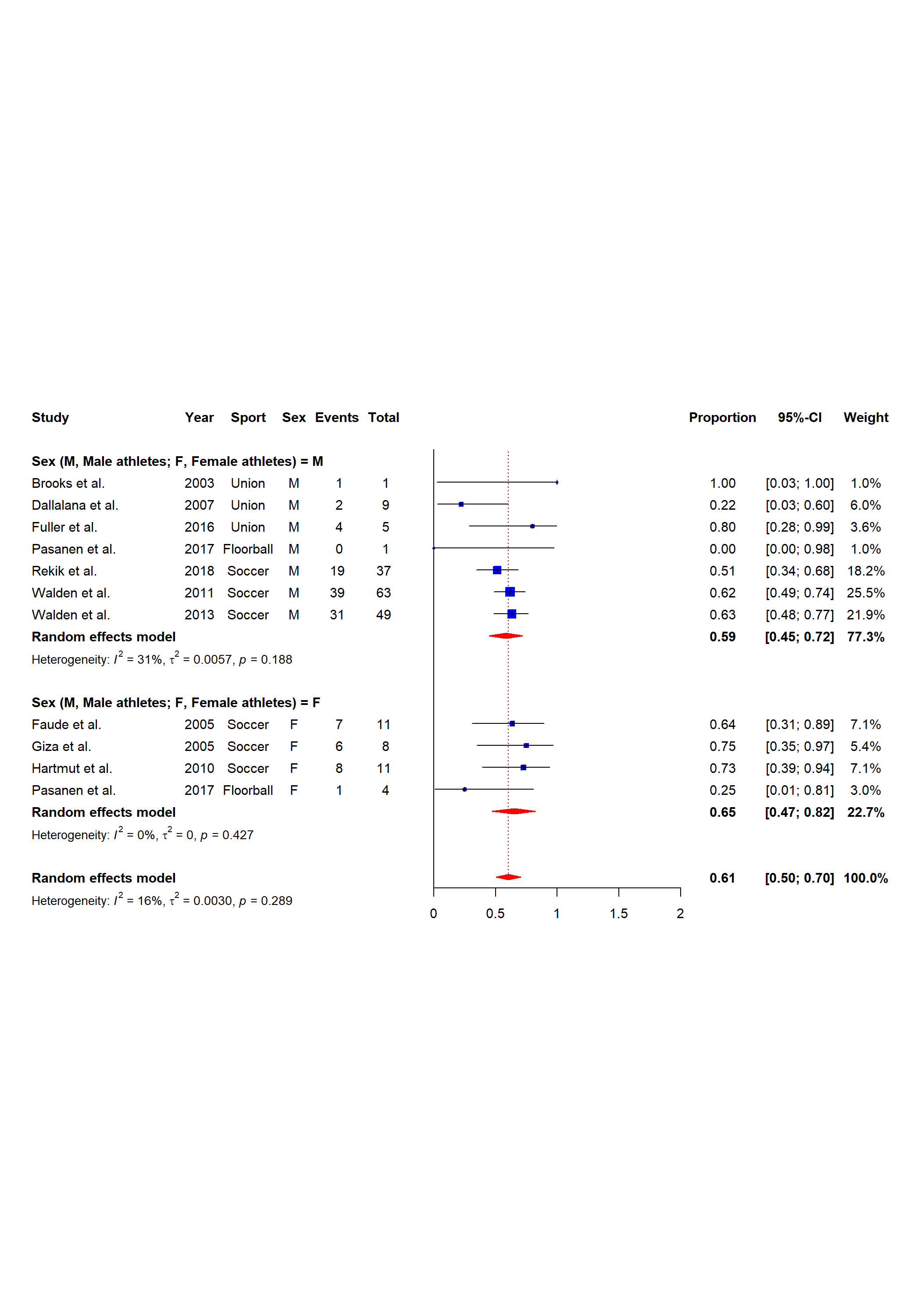


**Figure A6-2** Forest plot of meta-analysis of proportion of non-contact ACL injuries to total ACL injuries in elite-level athletes by sex

**Figure A6-3** Forest plot of meta-analysis of proportion of non-contact ACL injuries to total ACL injuries in males by participation level


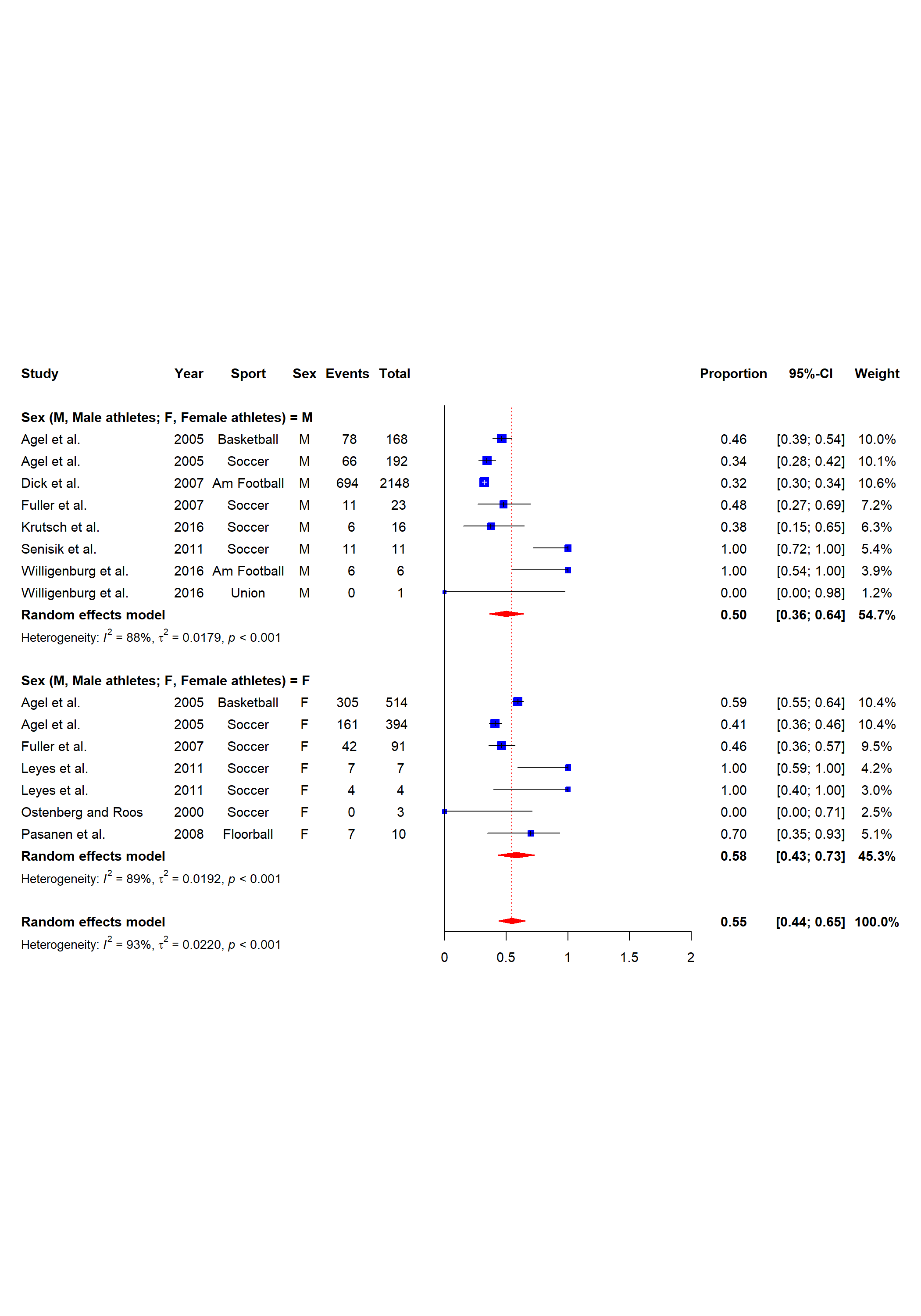


**Figure A6-3** Forest plot of meta-analysis of proportion of non-contact ACL injuries to total ACL injuries in intermediate-level athletes by sex


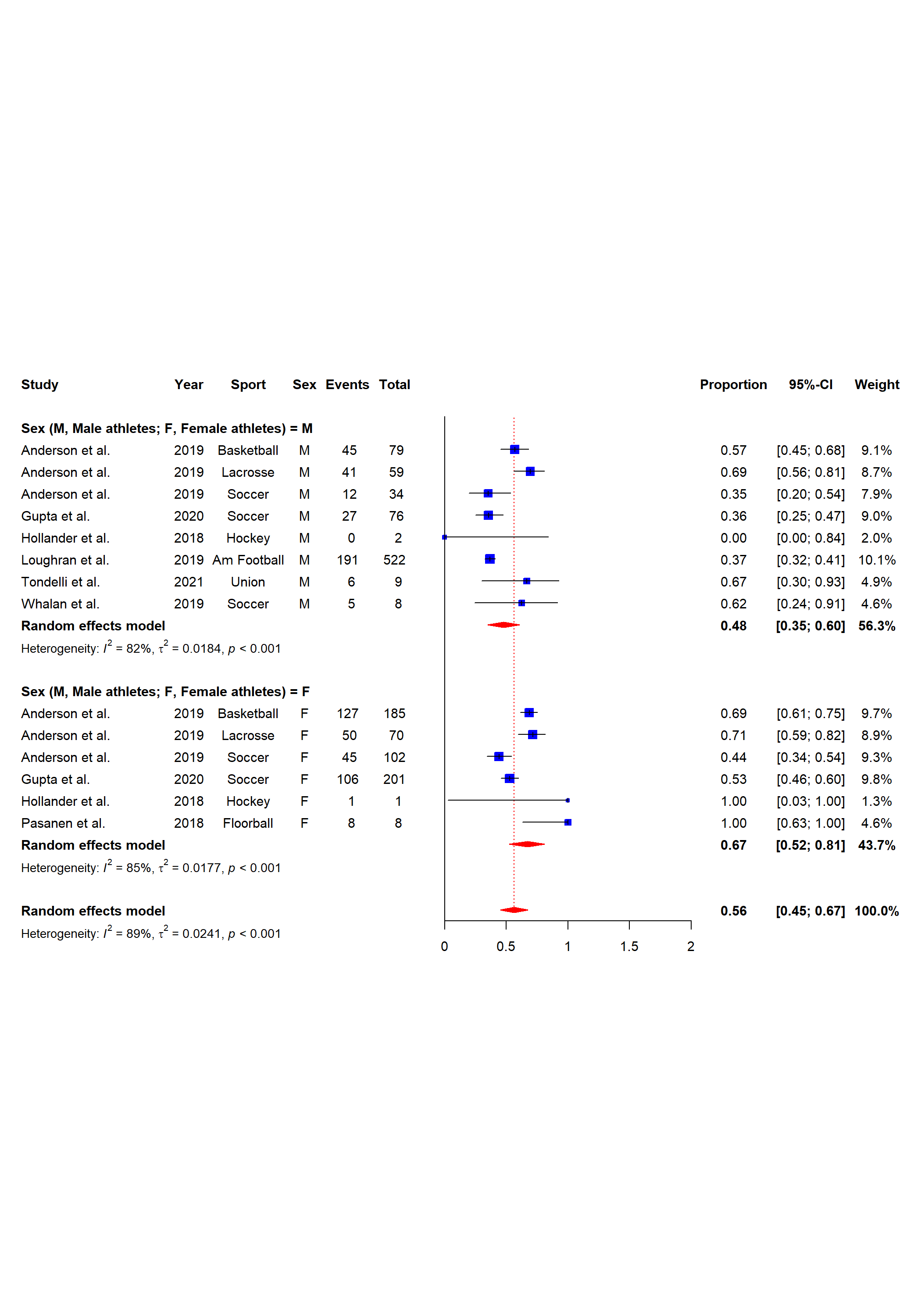


**Figure A6-4** Forest plot of meta-analysis of proportion of non-contact ACL injuries to total ACL injuries in amateur-level athletes by sex

**A7 FOREST PLOTS OF META-ANALYSIS OF PROPORTION OF NON-CONTACT ACL INJURIES TO TOTAL ACL INJURIES BY EXPOSURE TYPE**


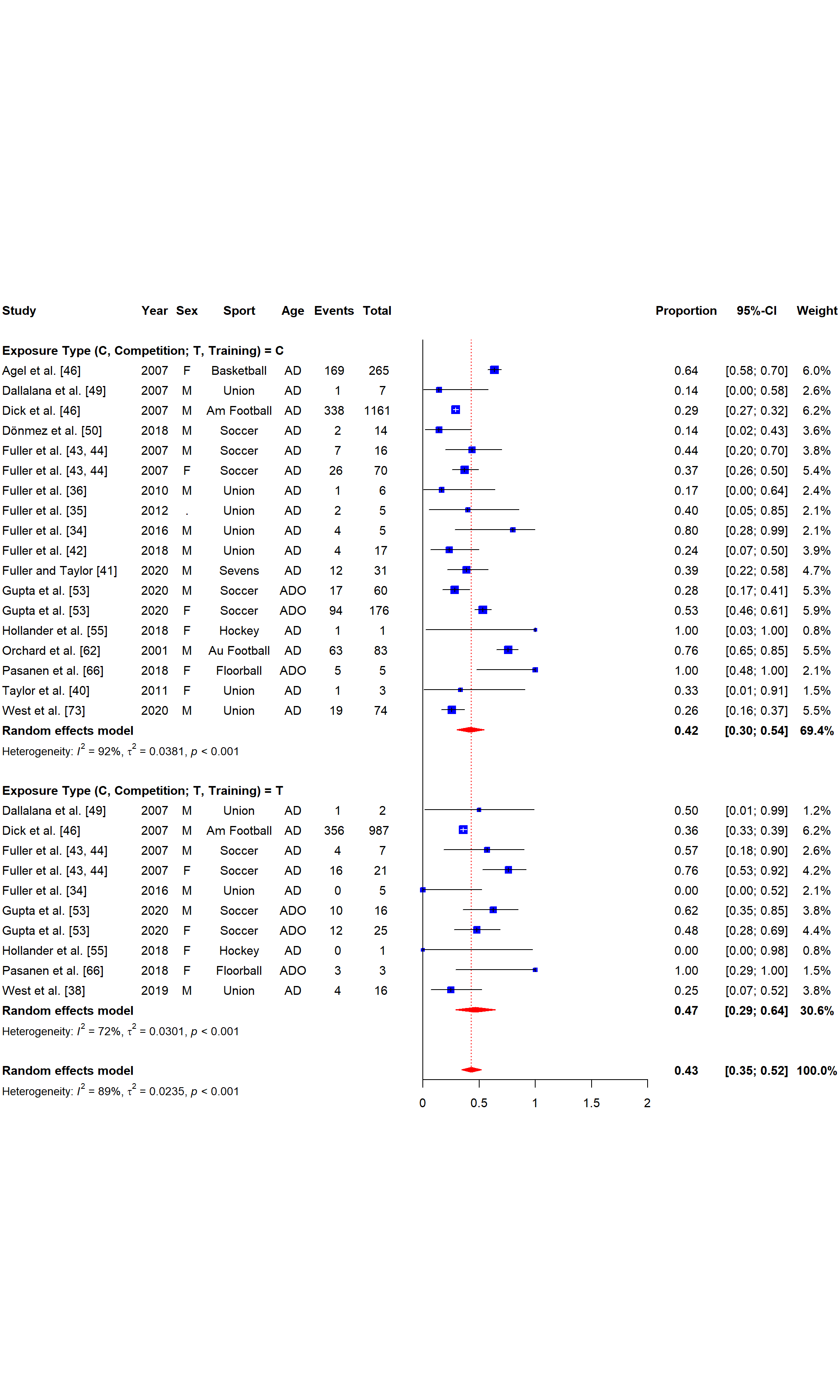


**Figure A7-1** Forest plot of meta-analysis of proportion of non-contact ACL injuries to total ACL injuries by exposure type


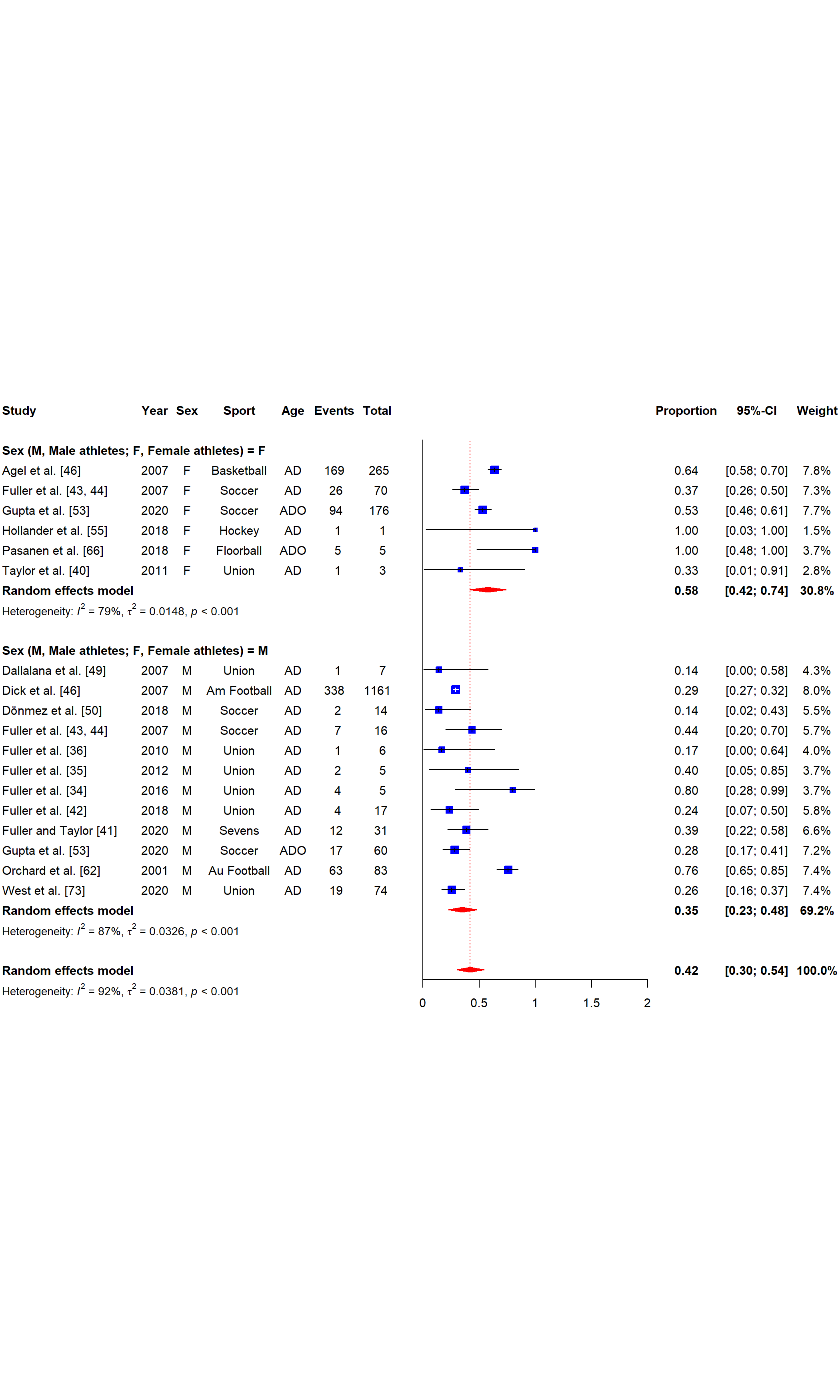


**Figure A7-2** Forest plot of meta-analysis of proportion of non-contact ACL injuries to total ACL injuries by exposure type in females


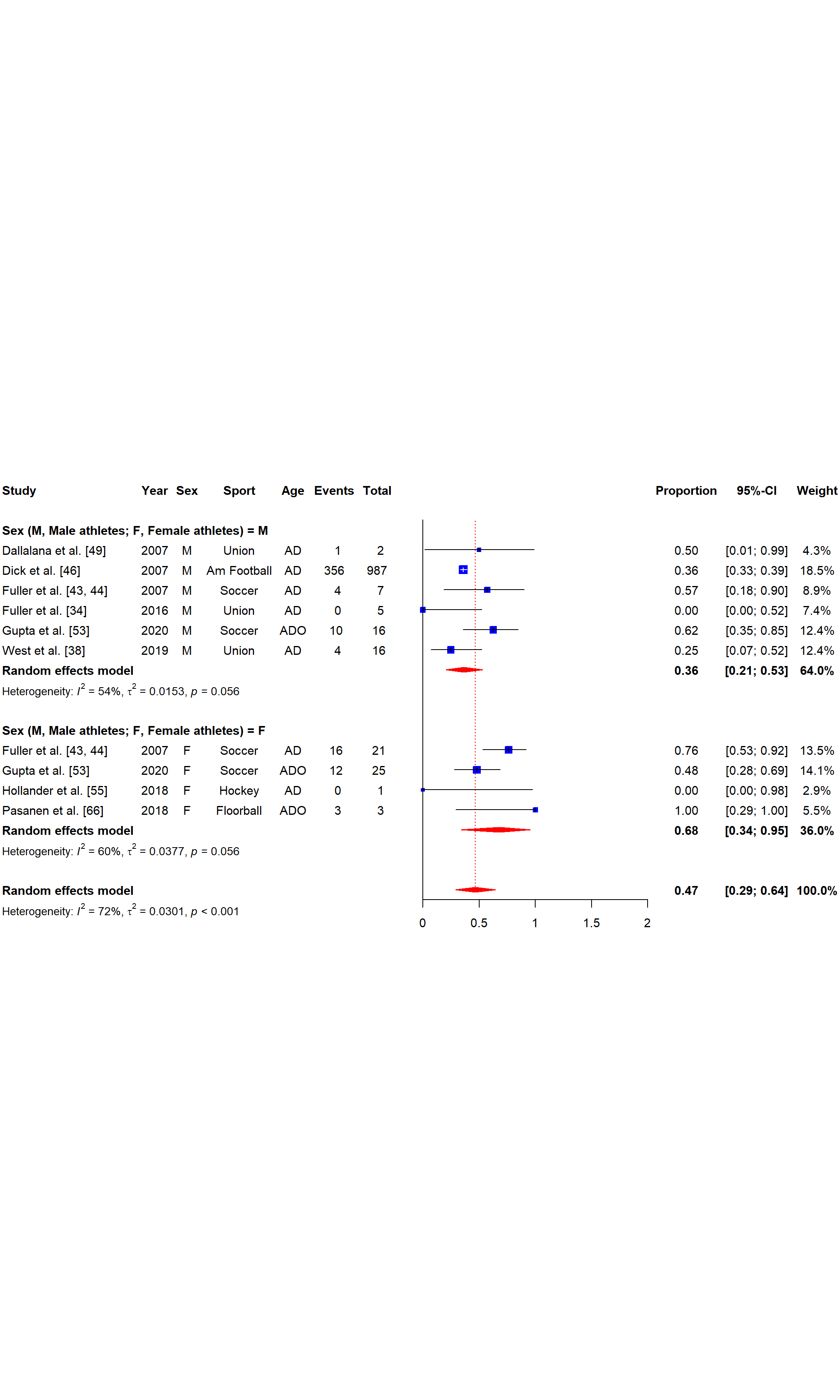


**Figure A7-3** Forest plot of meta-analysis of proportion of non-contact ACL injuries to total ACL injuries by exposure type in males

**A8 FOREST PLOTS OF META-ANALYSIS OF INCIDENCE OF NON-CONTACT ACL INJURIES BY SPORT**


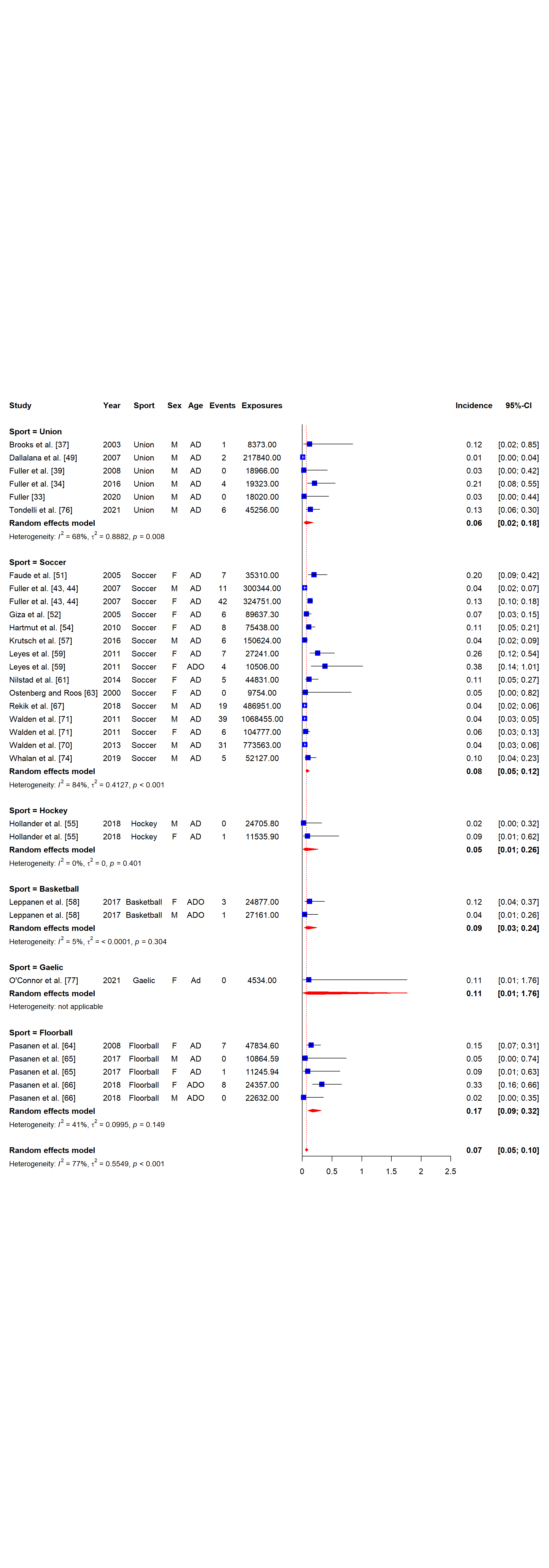


*Continued next page*

**Figure A8-1** Forest plot of meta-analysis of incidence of non-contact ACL injuries per 1000 player-hours by sport


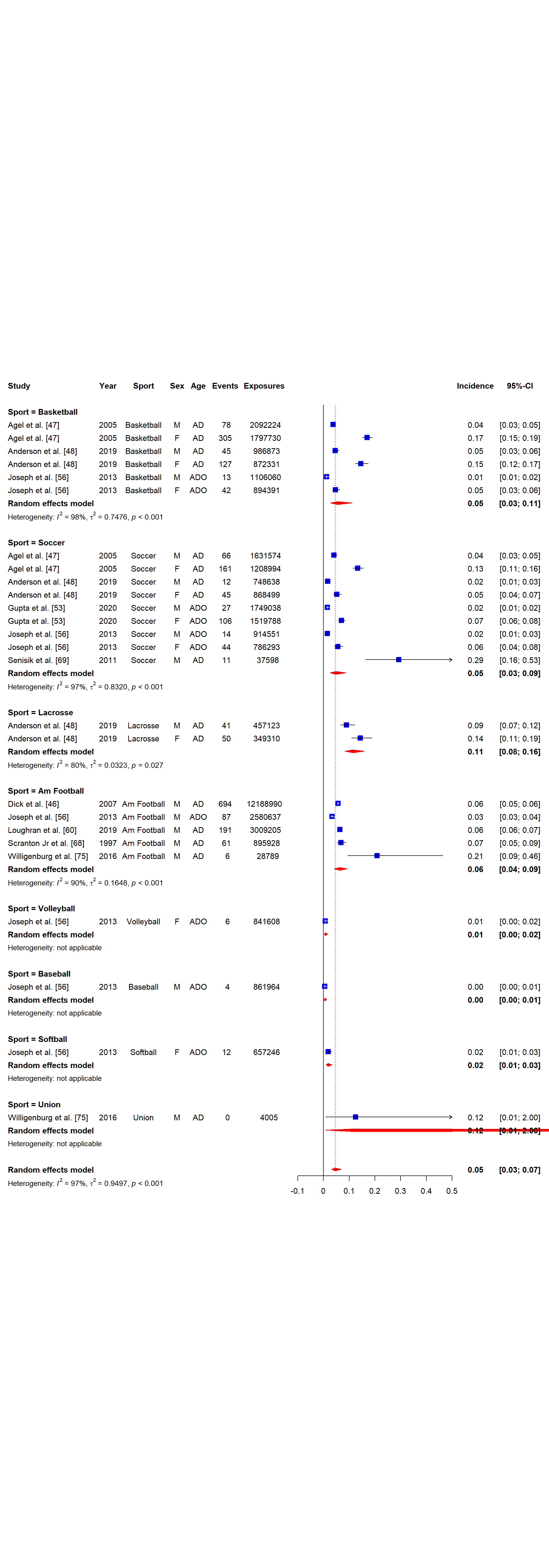


*Continued next page*


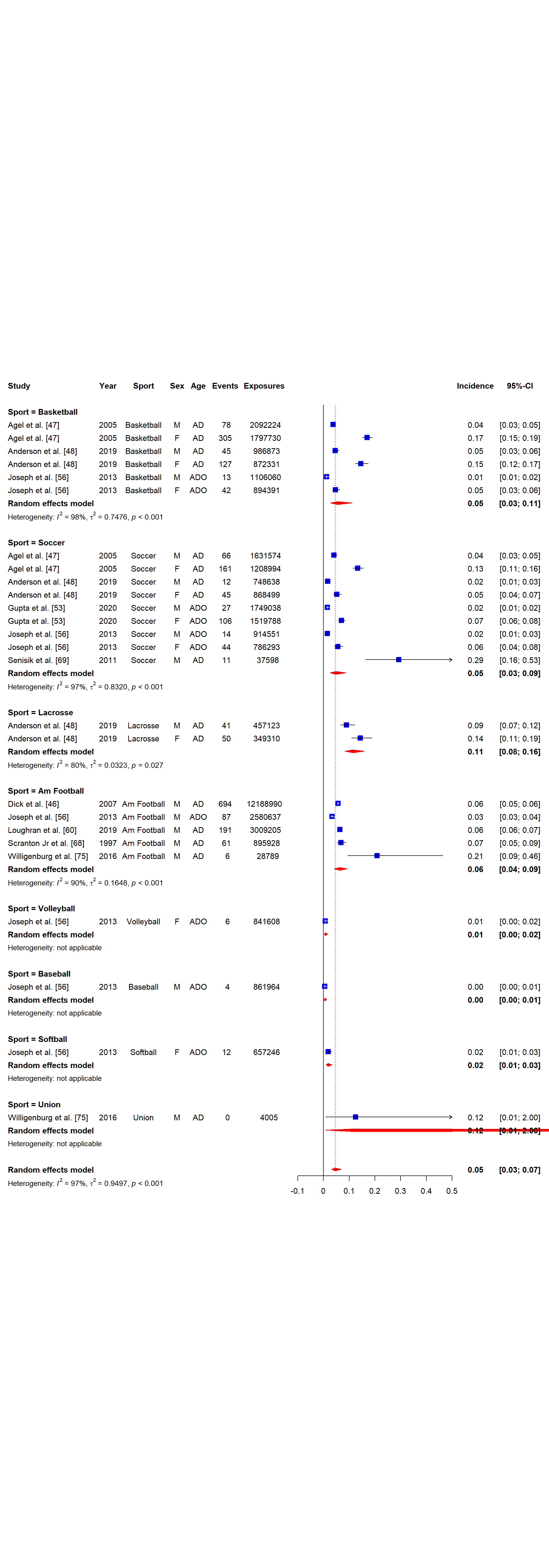


**Figure A8-2** Forest plot of meta-analysis of incidence of non-contact ACL injuries per 1000 player-exposures by sport


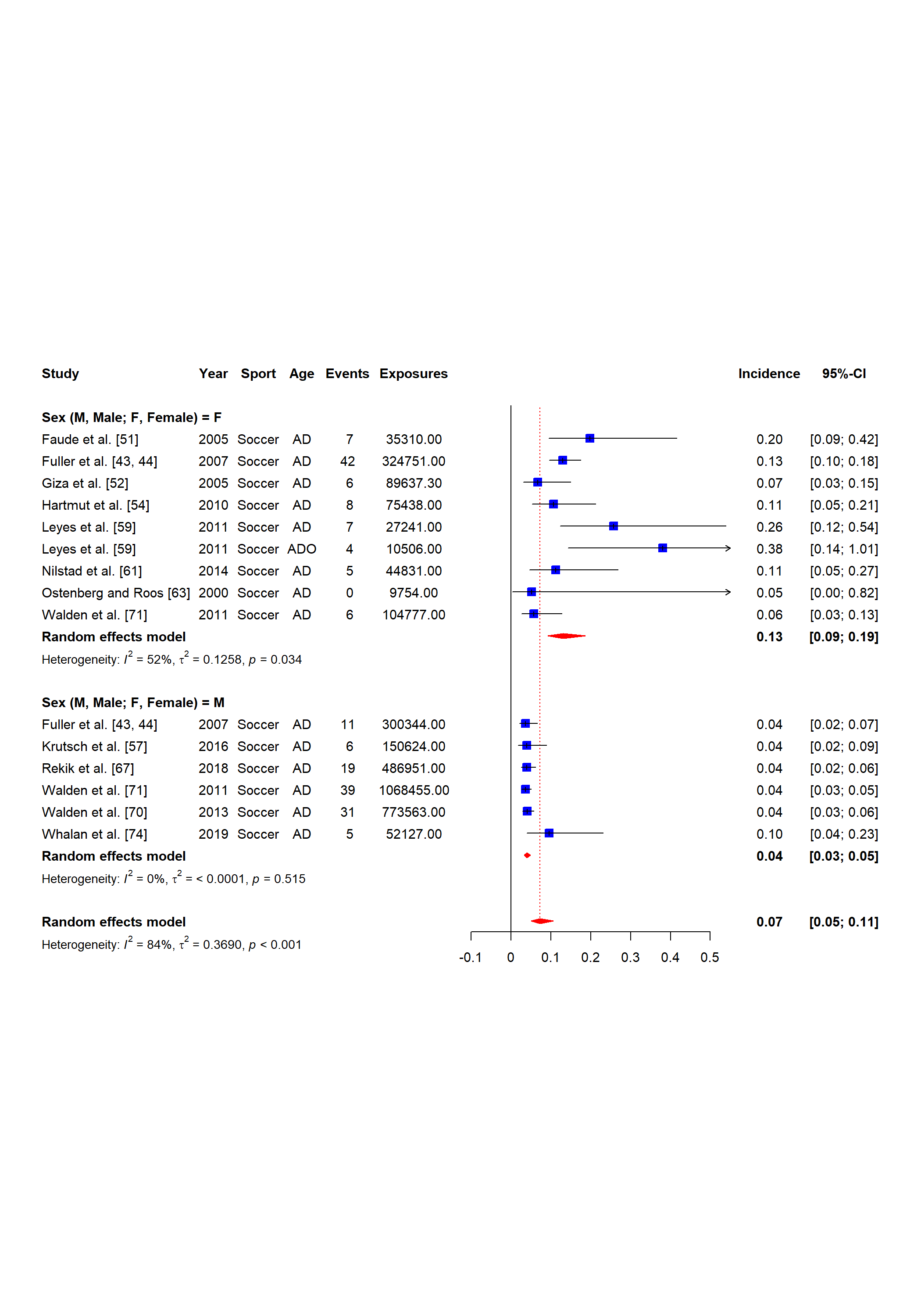


**Figure A8-3** Forest plot of meta-analysis of incidence of non-contact ACL injuries per 1000 player-hours in soccer by sex


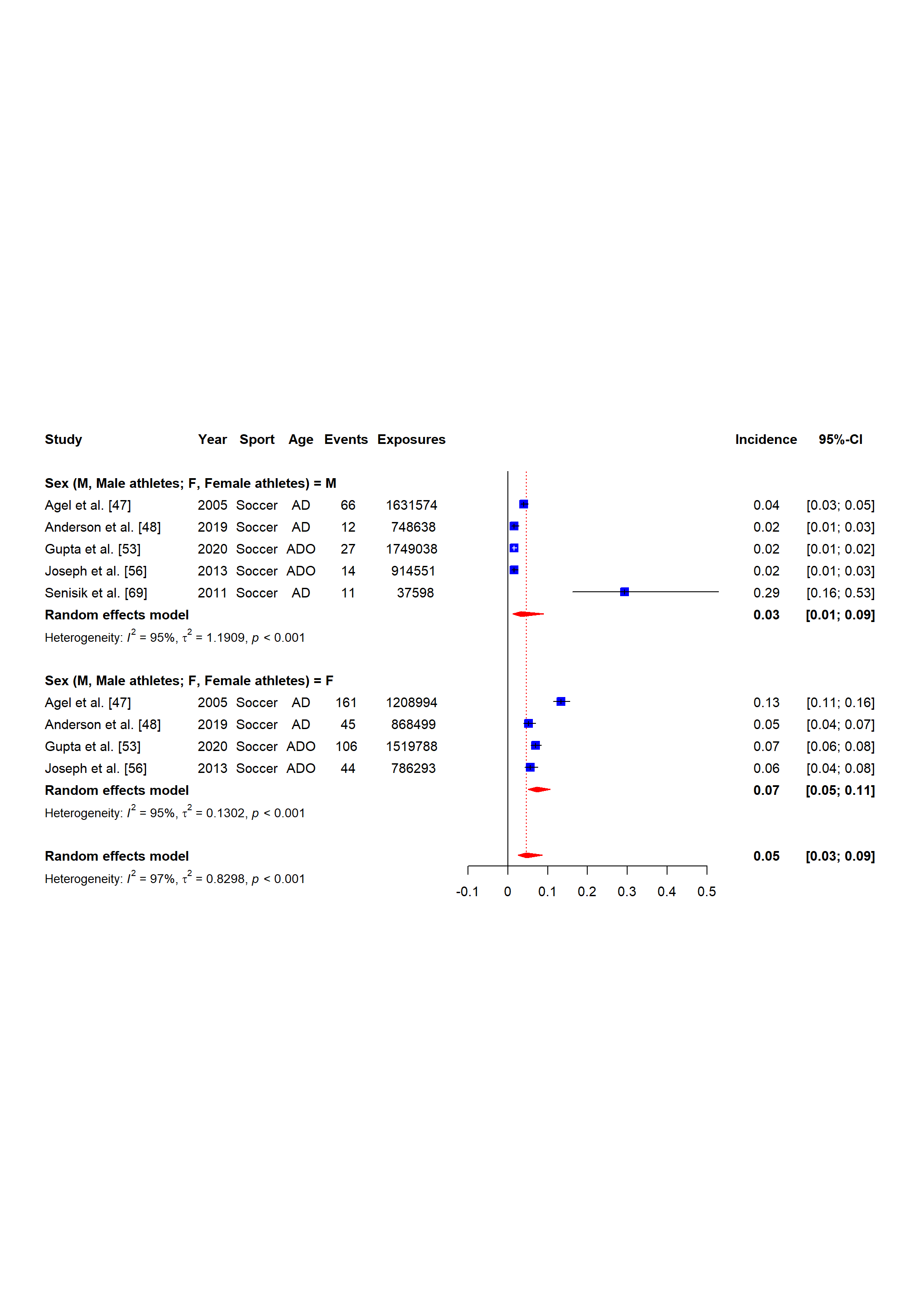


**Figure A8-4** Forest plot of meta-analysis of incidence of non-contact ACL injuries per 1000 player-exposures in soccer by sex


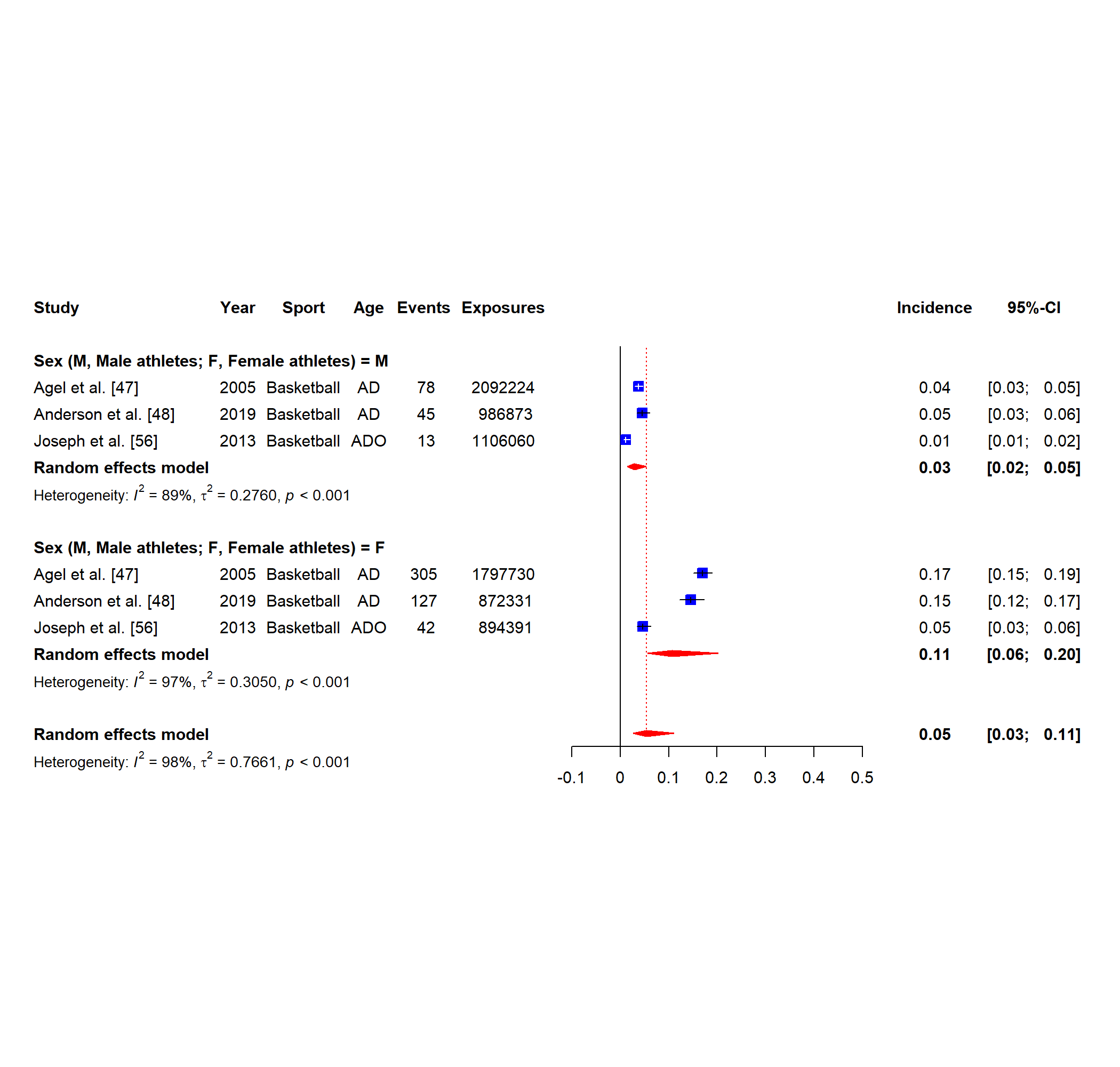


**Figure A8-5** Forest plot of meta-analysis of incidence of non-contact ACL injuries per 1000 player-exposures in basketball by sex

**A9 FOREST PLOT OF META-ANALYSIS OF INCIDENCE OF NON-CONTACT ACL INJURIES BY AGE GROUP**


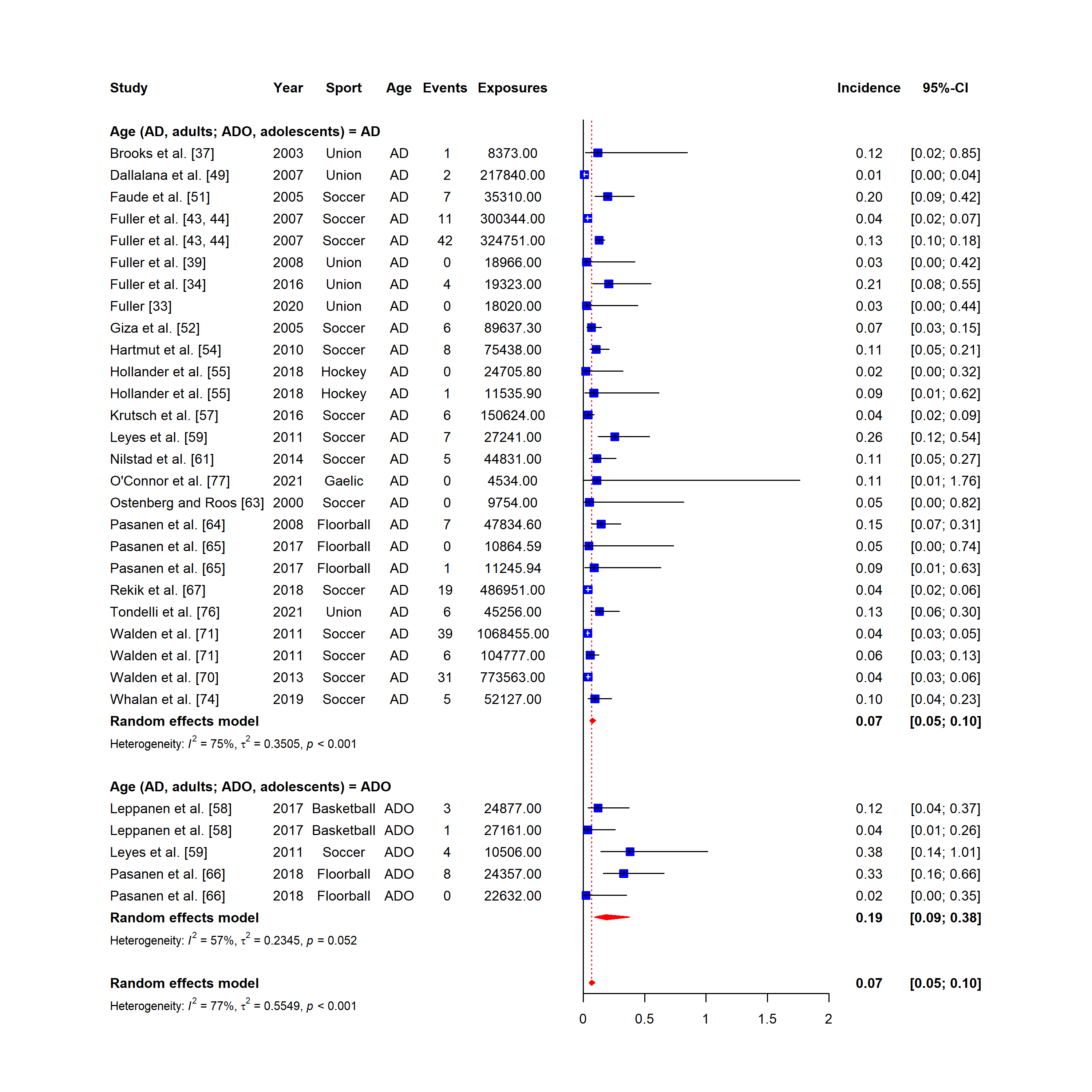


**Figure A9-1** Forest plot of meta-analysis of incidence of non-contact ACL injuries per 1000 player-hours by age group


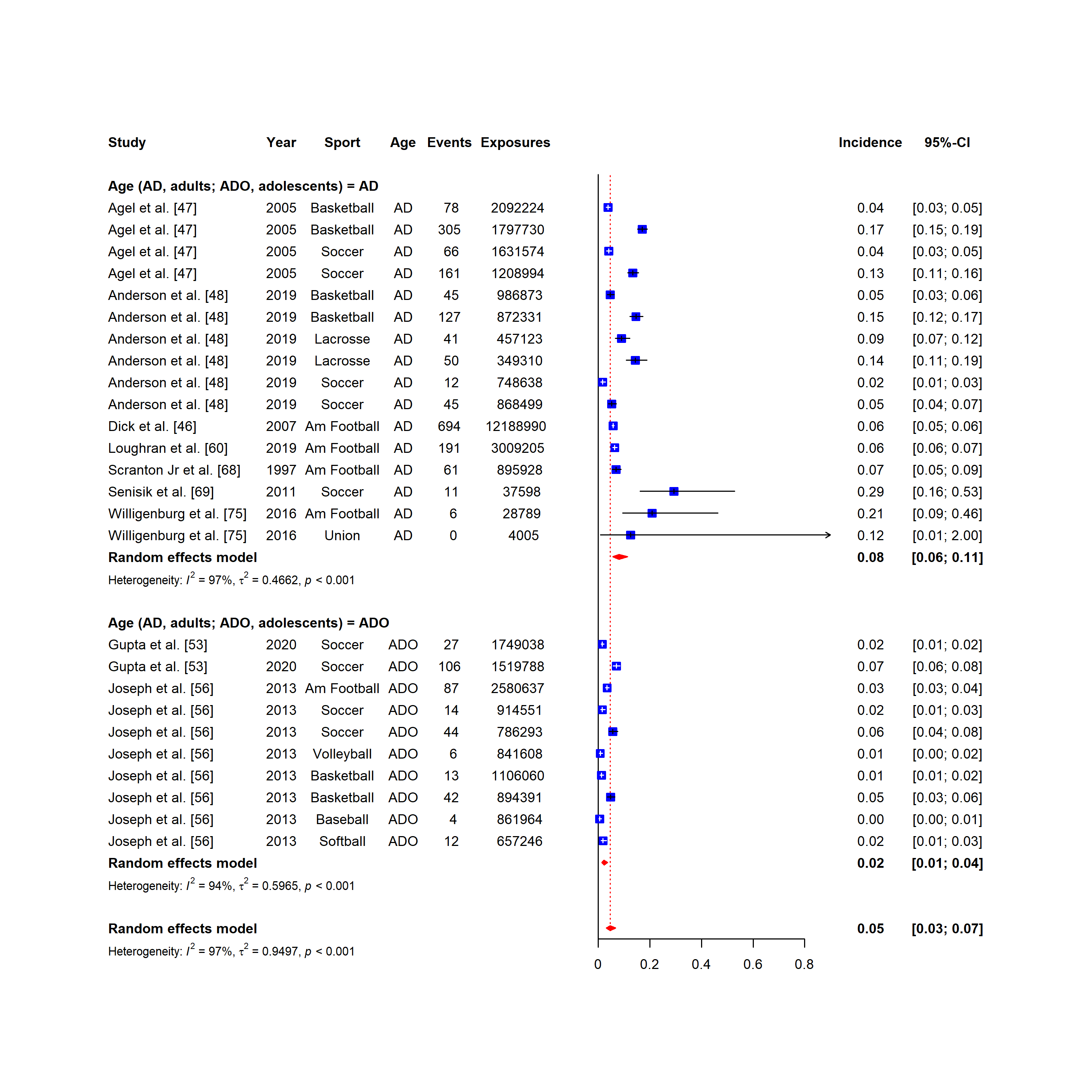


**Figure A9-2** Forest plot of meta-analysis of incidence of non-contact ACL injuries per 1000 player-exposures by age group


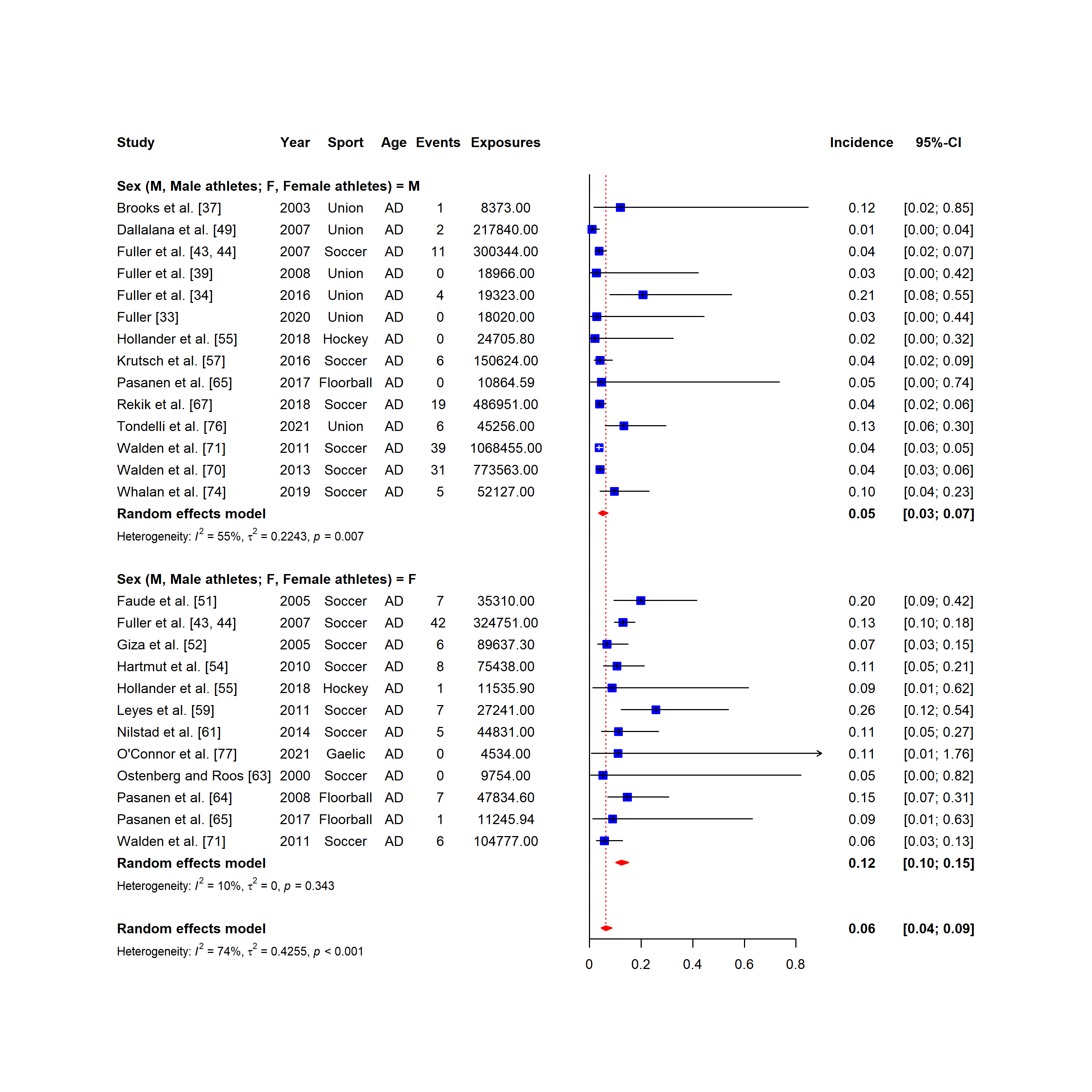


**Figure A9-3** Forest plot of meta-analysis of incidence of non-contact ACL injuries per 1000 player-hours in adults


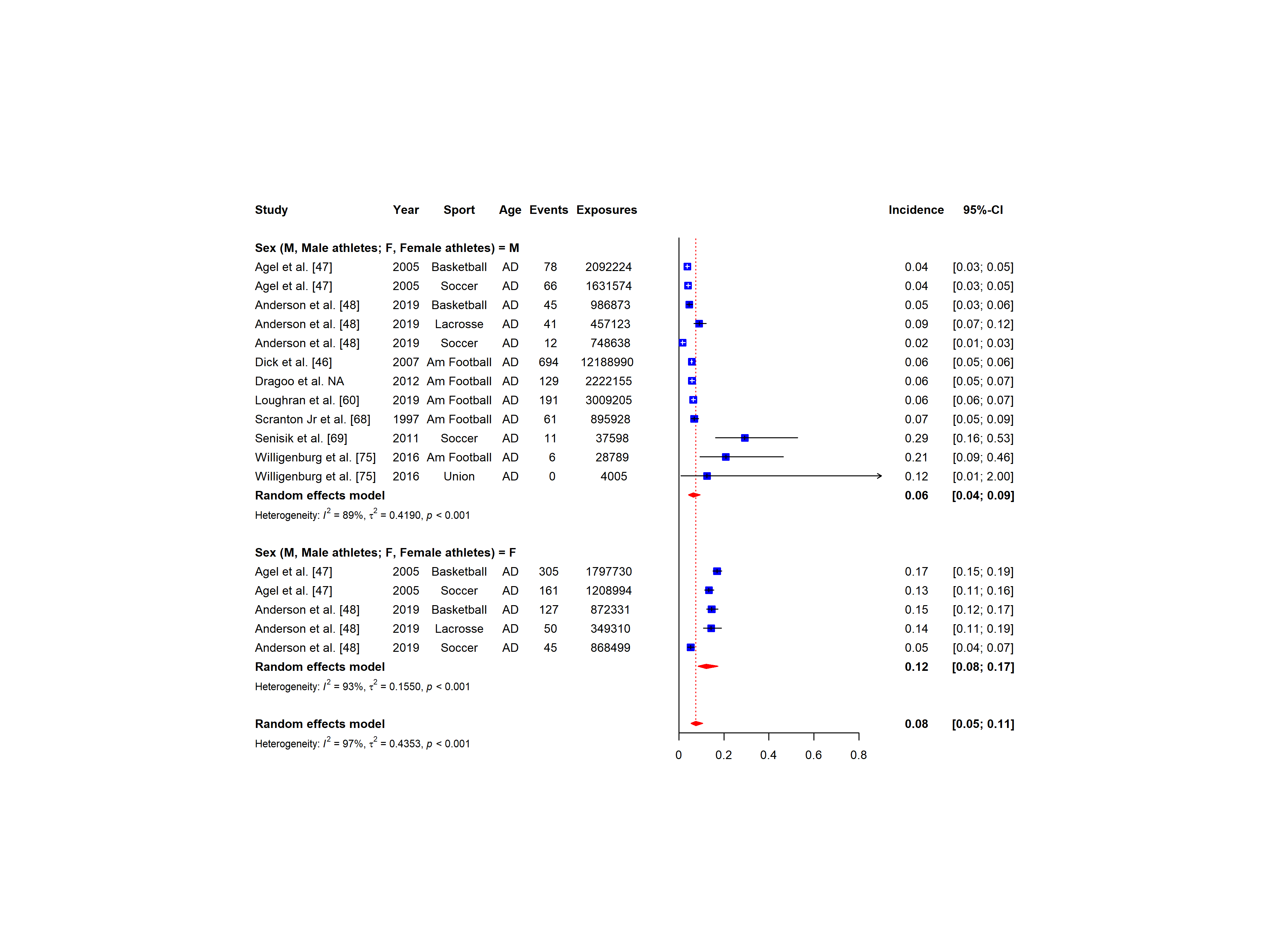


**Figure A9-4** Forest plot of meta-analysis of incidence of non-contact ACL injuries per 1000 player-exposures in adults


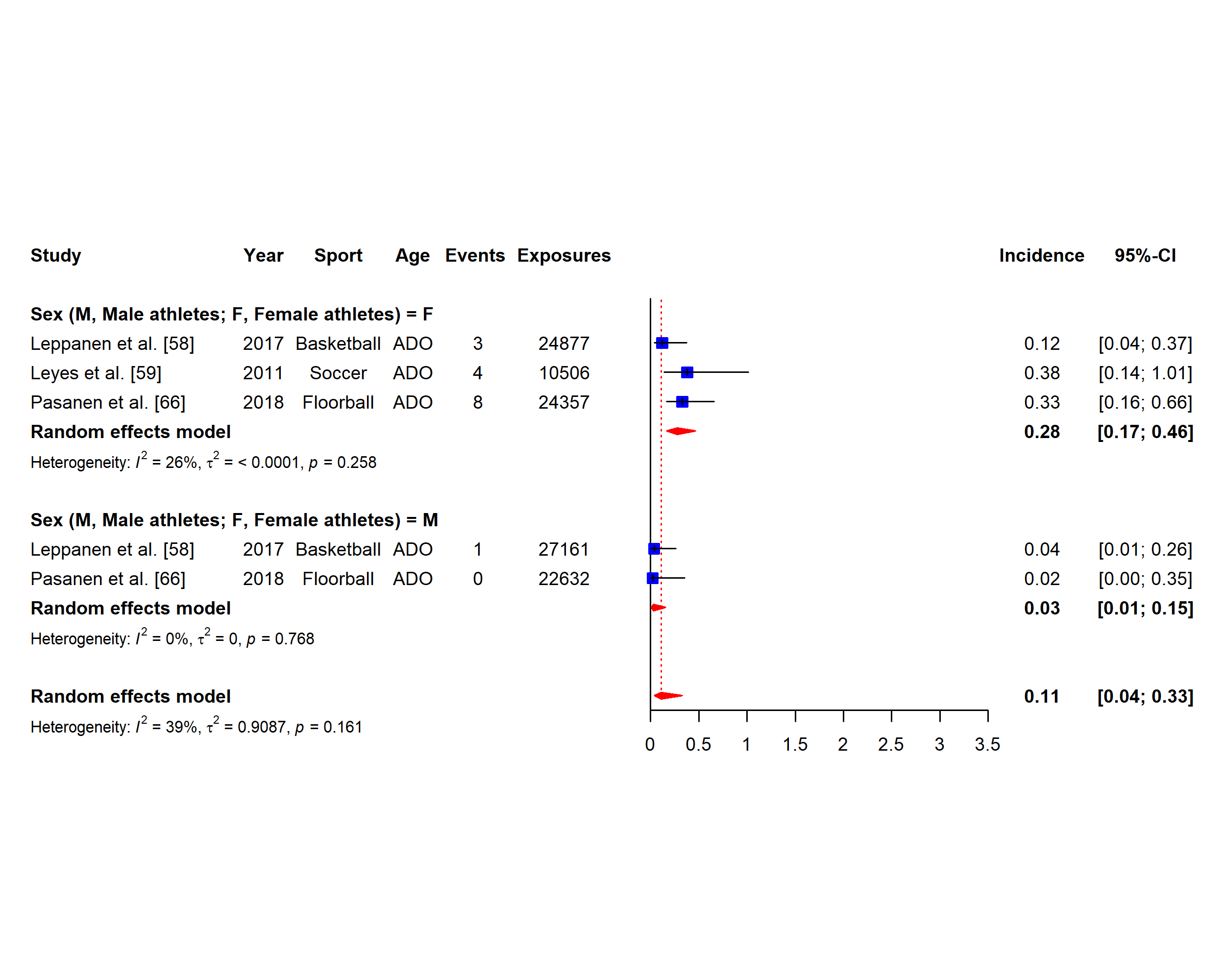


**Figure A9-5** Forest plot of meta-analysis of incidence of non-contact ACL injuries per 1000 player-hours in adolescents


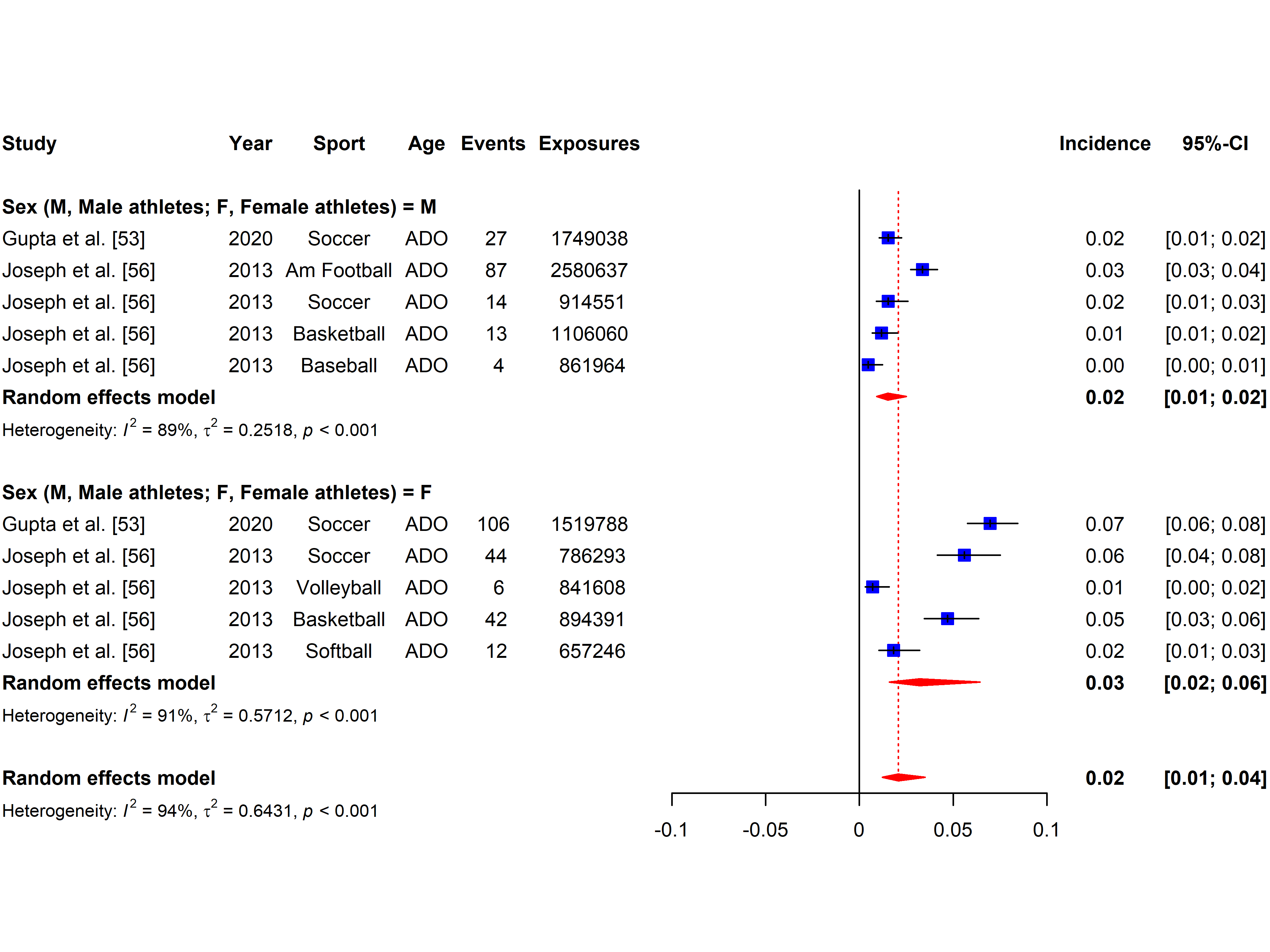


**Figure A9-6** Forest plot of meta-analysis of incidence of non-contact ACL injuries per 1000 player-exposures in adolescents

**A10 FOREST PLOT OF META-ANALYSIS OF INCIDENCE OF NON-CONTACT ACL INJURIES BY PARTICIPATION LEVEL**


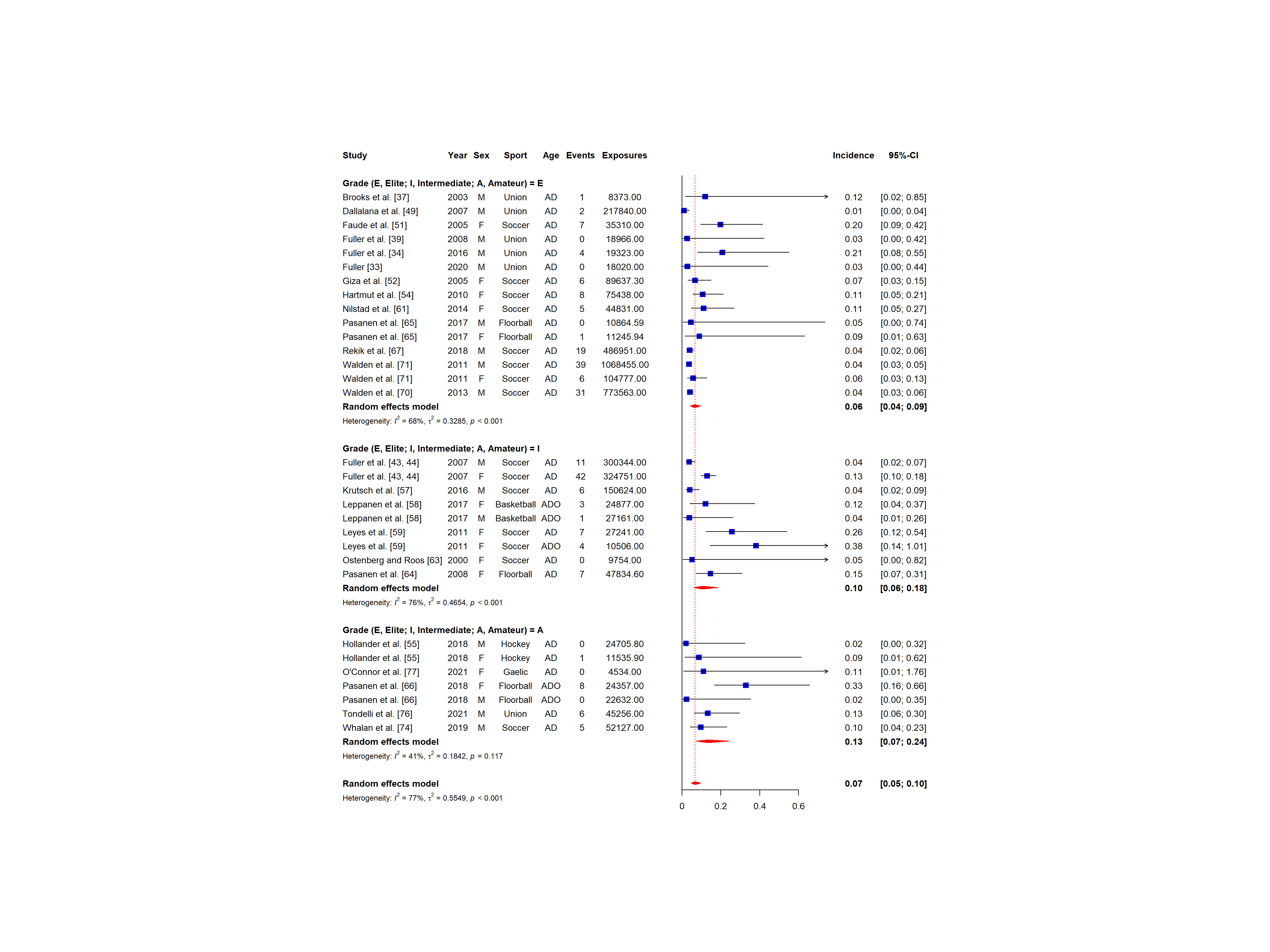


**Figure A10-1** Forest plot of meta-analysis of incidence of non-contact ACL injuries per 1000 player-hours by participation level


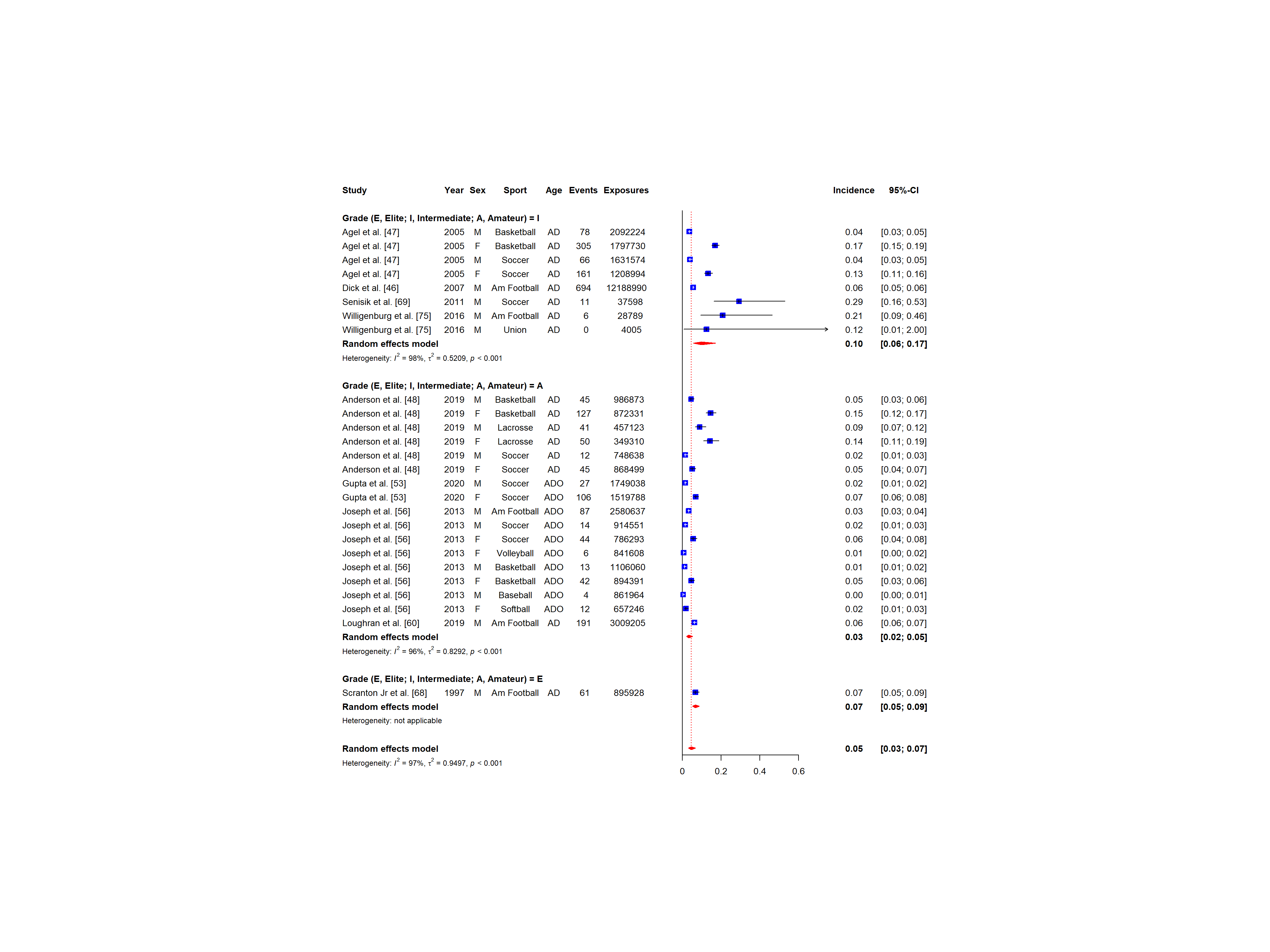


**Figure A10-2** Forest plot of meta-analysis of incidence of non-contact ACL injuries per 1000 player-exposures by participation level


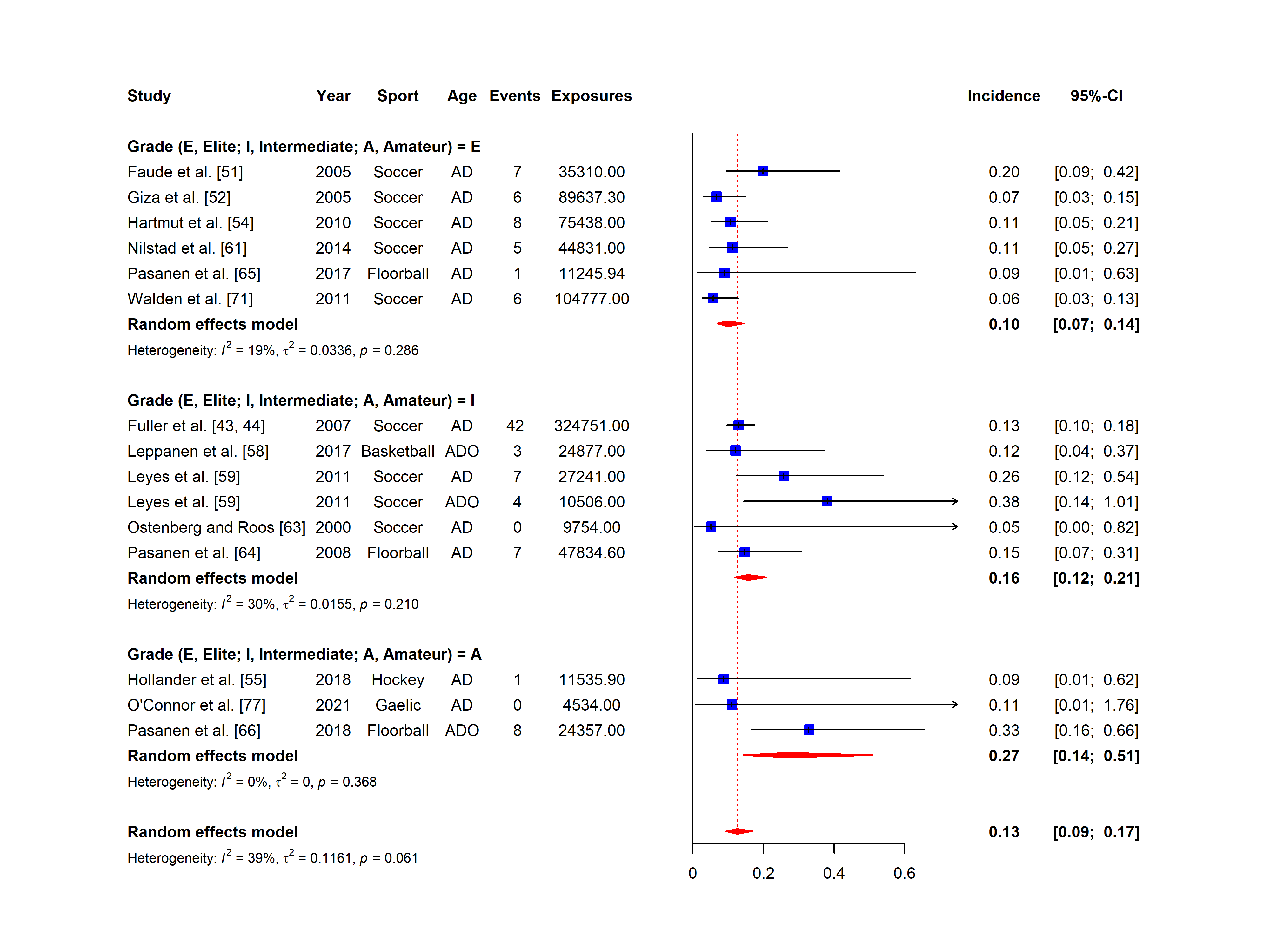


**Figure A10-3** Forest plot of meta-analysis of incidence of non-contact ACL injuries in elite-level athletes per 1000 player-hours by sex


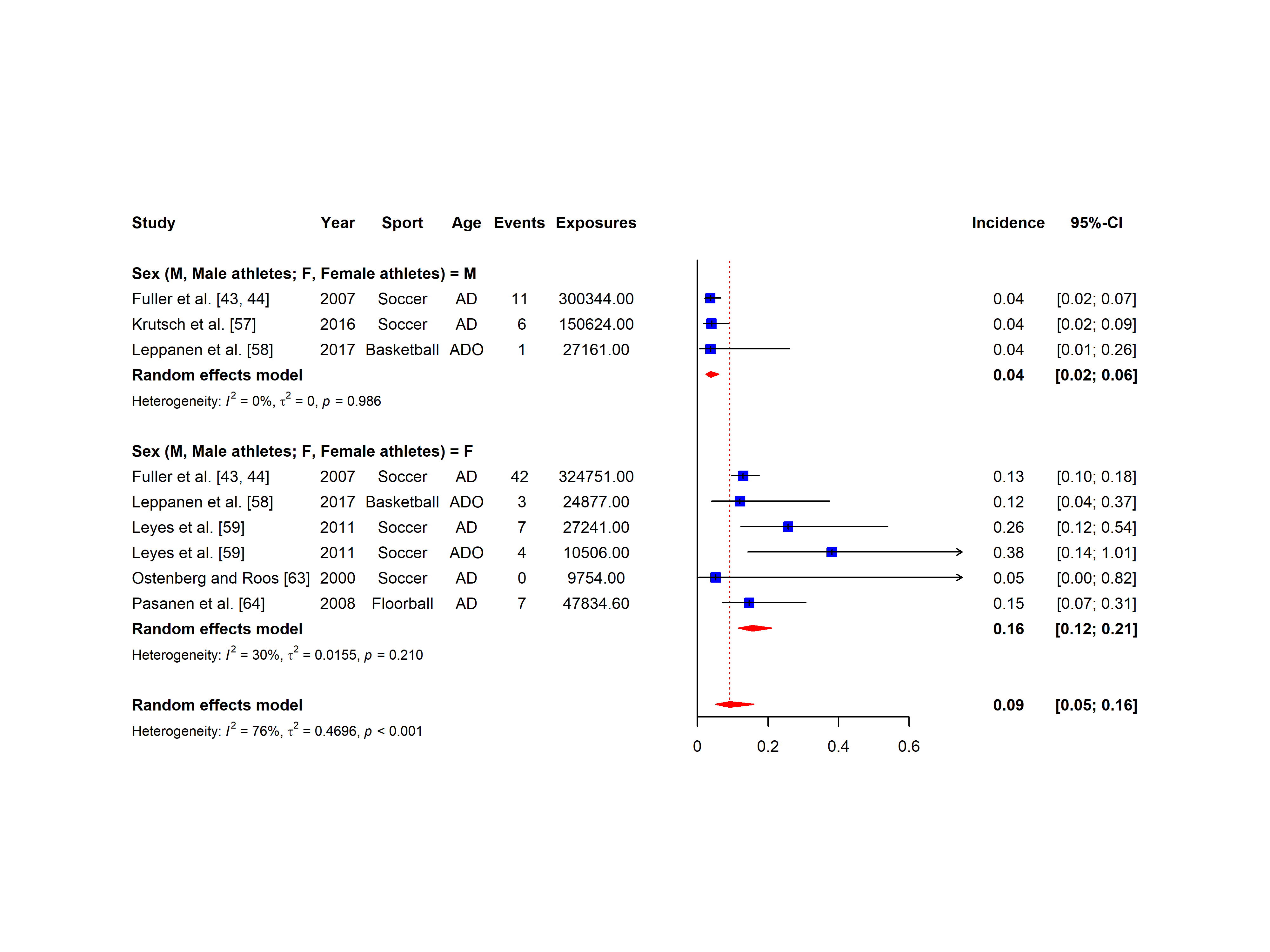


**Figure A10-4** Forest plot of meta-analysis of incidence of non-contact ACL injuries in intermediate-level athletes per 1000 player-hours by sex


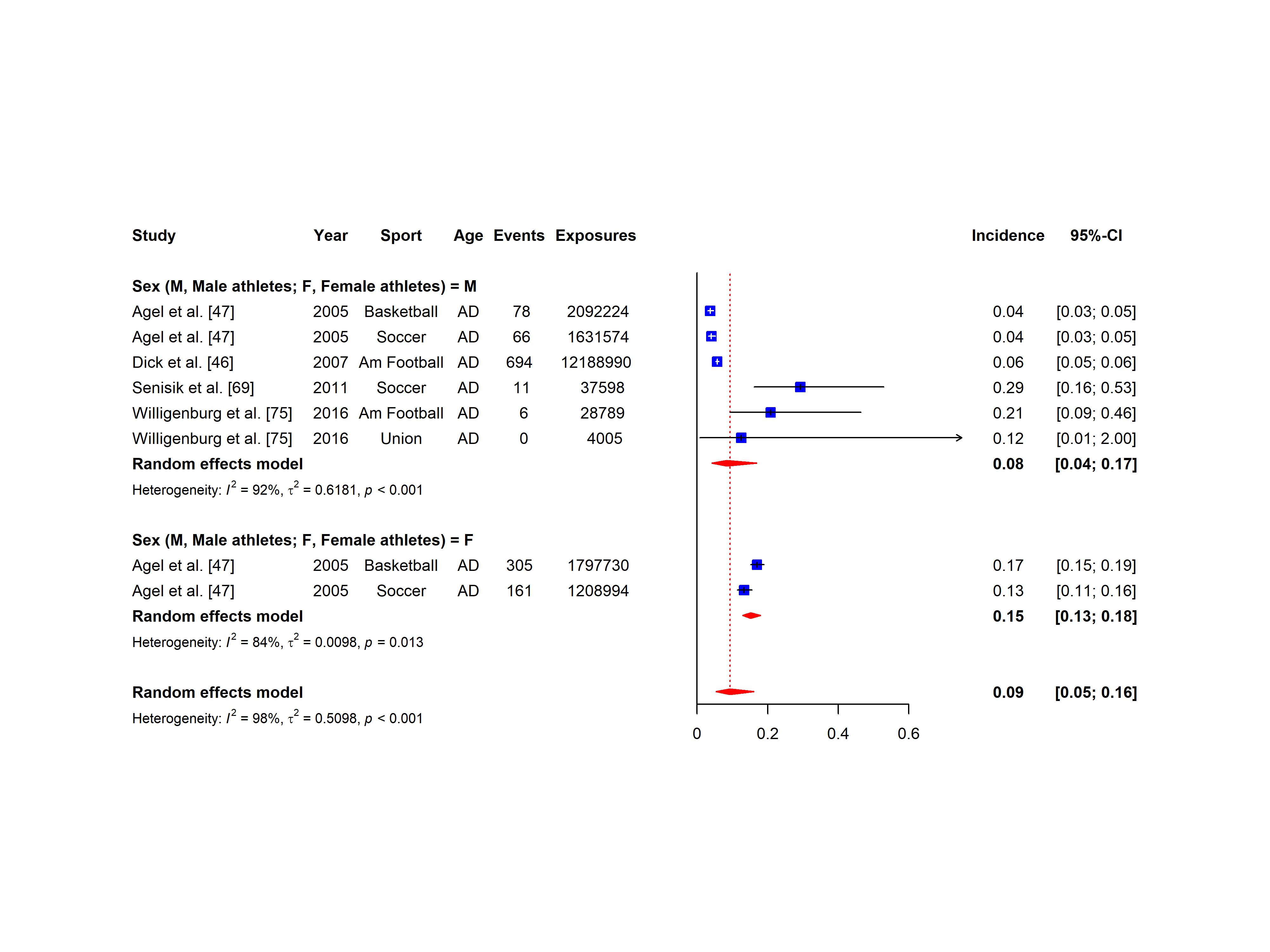


**Figure A10-5** Forest plot of meta-analysis of incidence of non-contact ACL injuries in intermediate-level athletes per 1000 player-exposures by sex


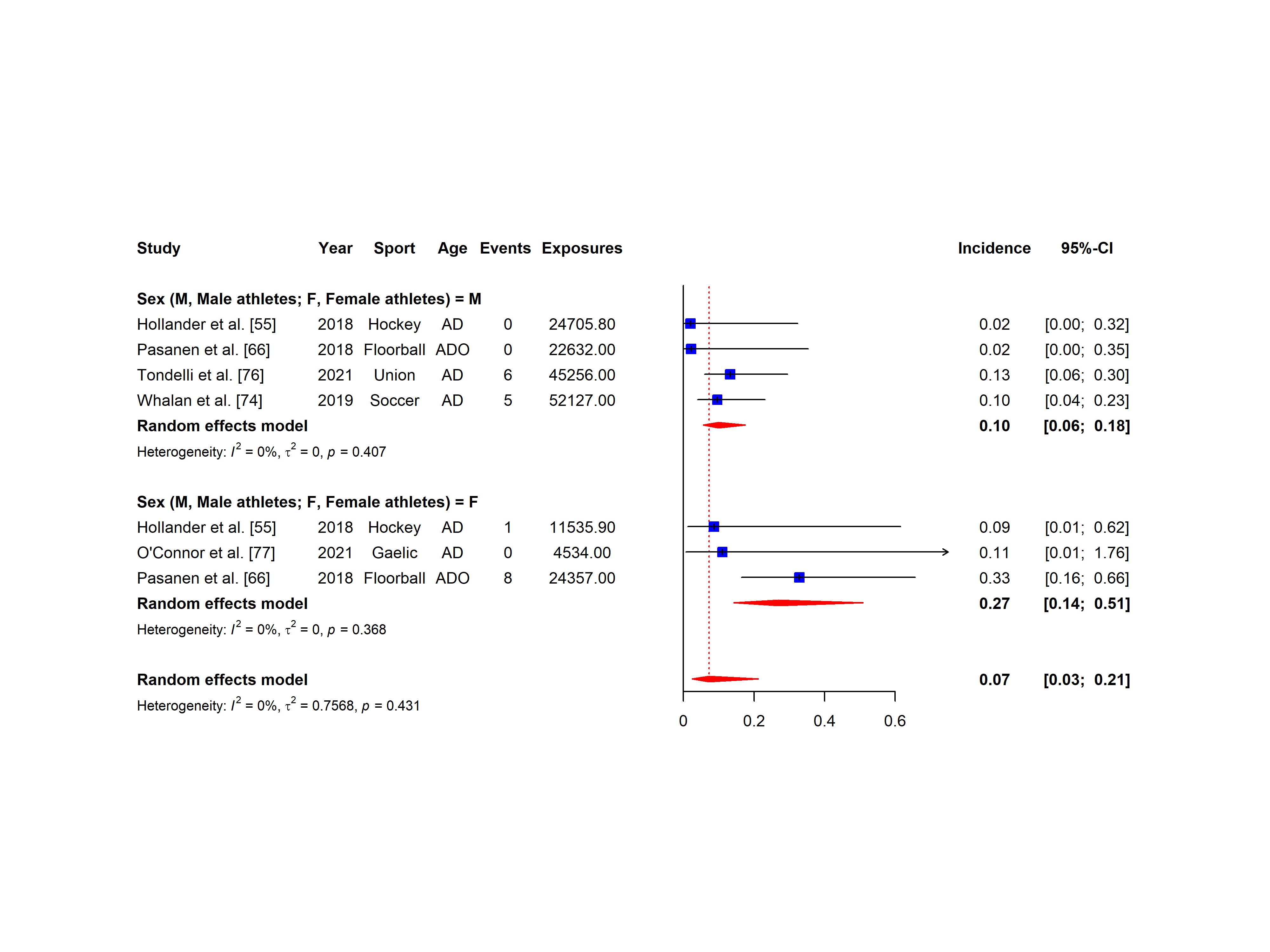


**Figure A10-6** Forest plot of meta-analysis of incidence of non-contact ACL injuries in amateur-level athletes per 1000 player-hours by sex

**A11 FOREST PLOT OF META-ANALYSIS OF INCIDENCE OF NON-CONTACT ACL INJURIES BY EXPOSURE TYPE**


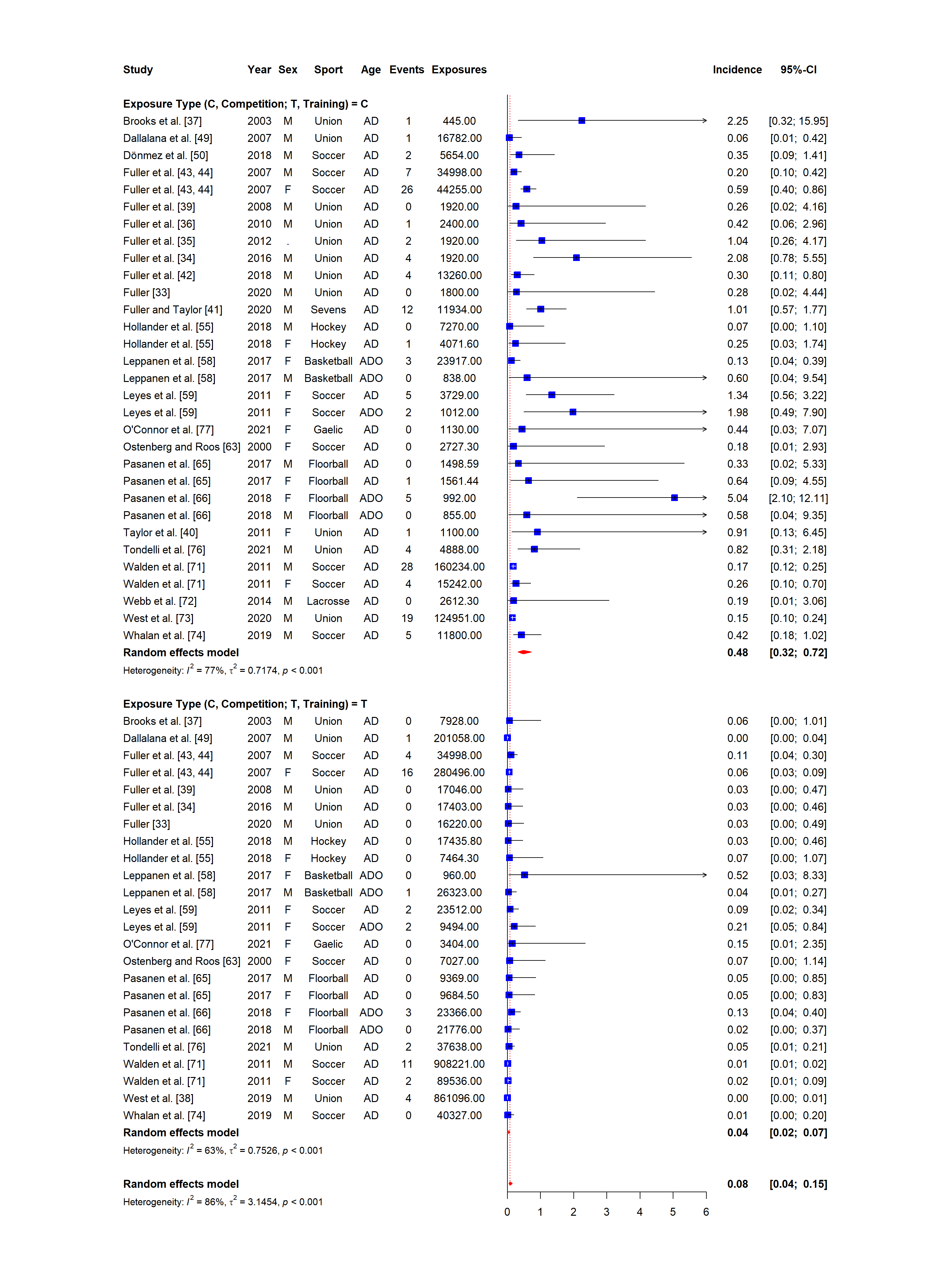


*Continued next page…*


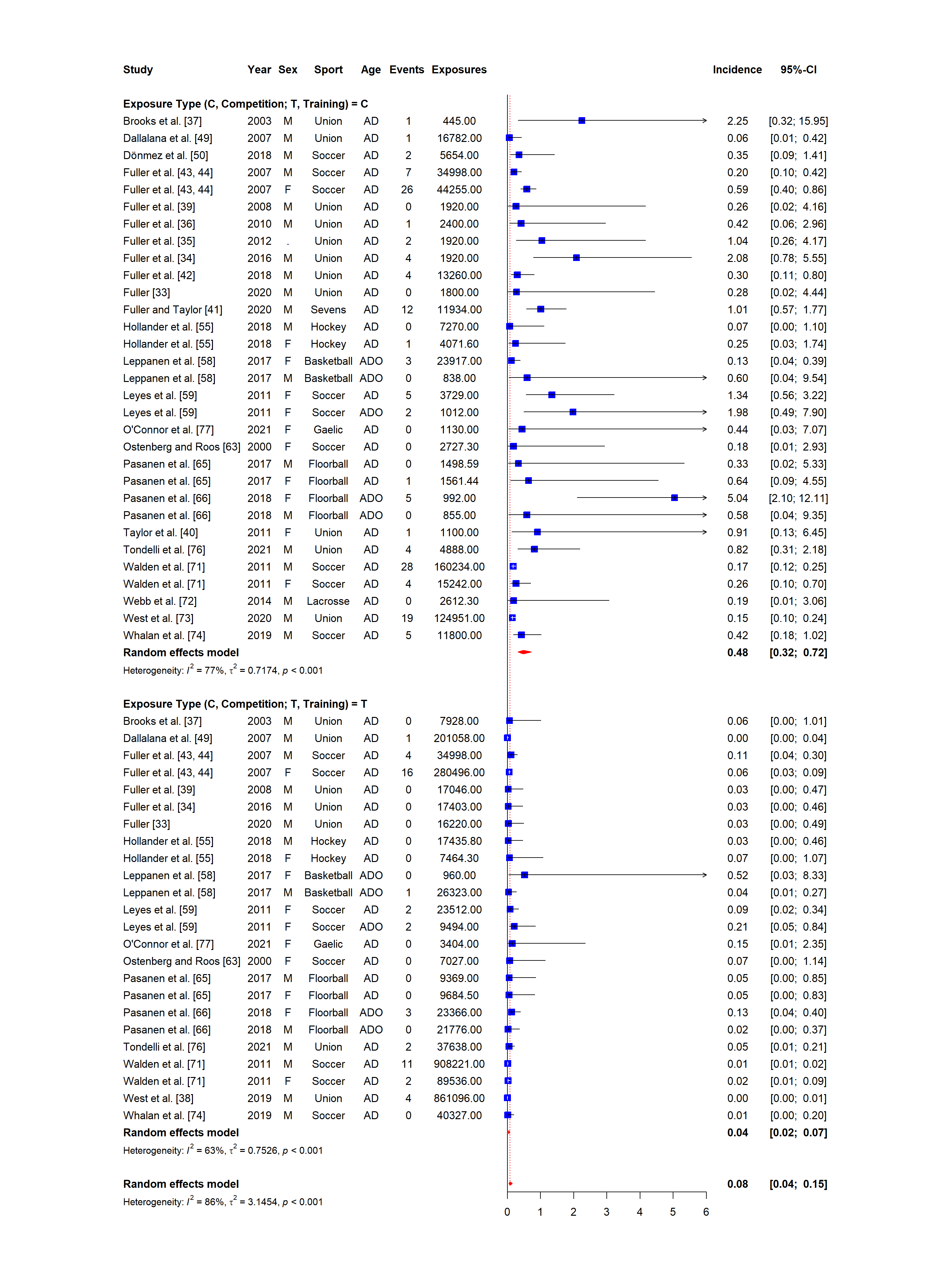


**Figure A11-1** Forest plot of meta-analysis of incidence of non-contact ACL injuries per 1000 player-hours by exposure type


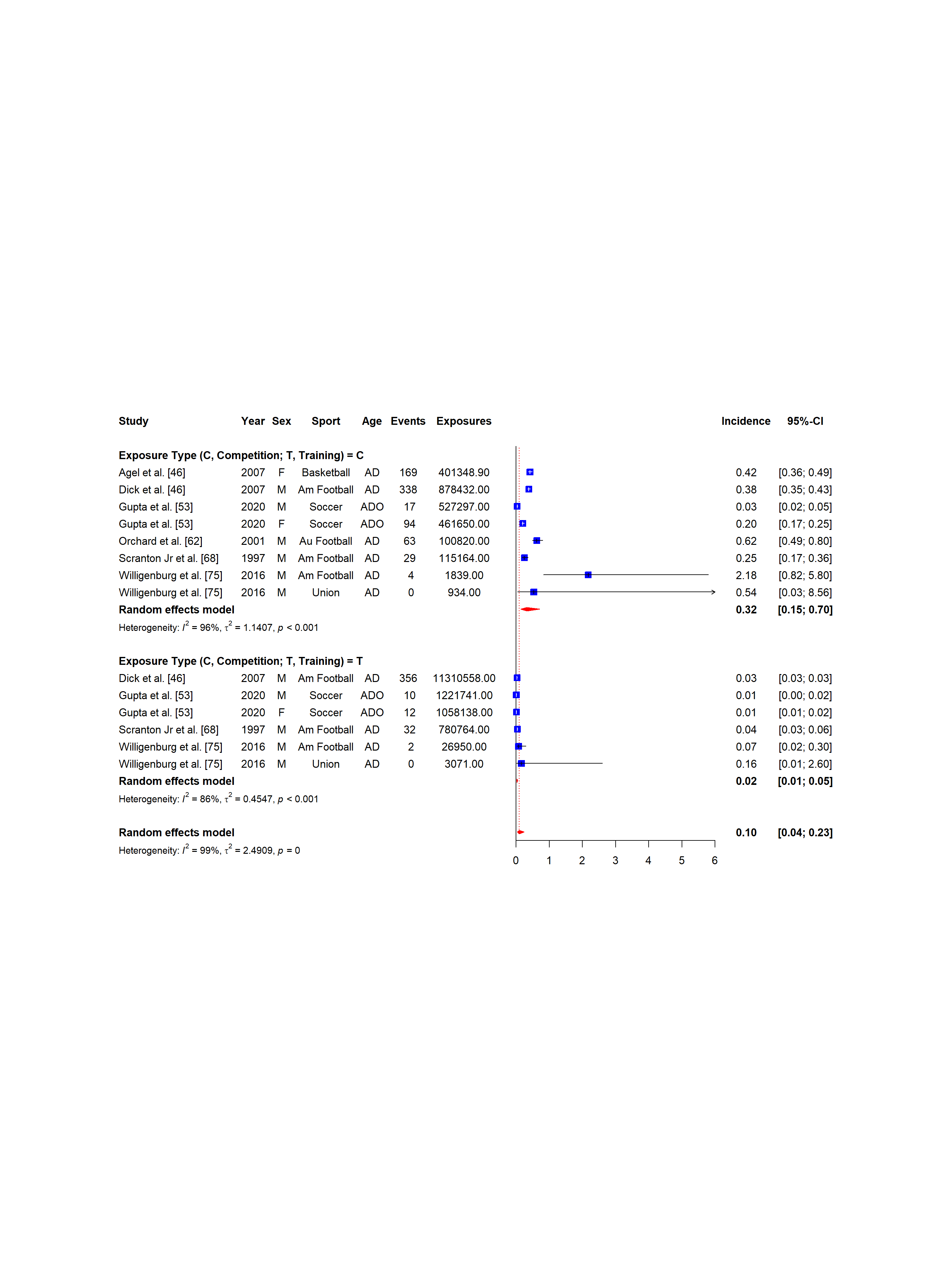


**Figure A11-2** Forest plot of meta-analysis of incidence of non-contact ACL injuries per 1000 player-exposures by exposure type


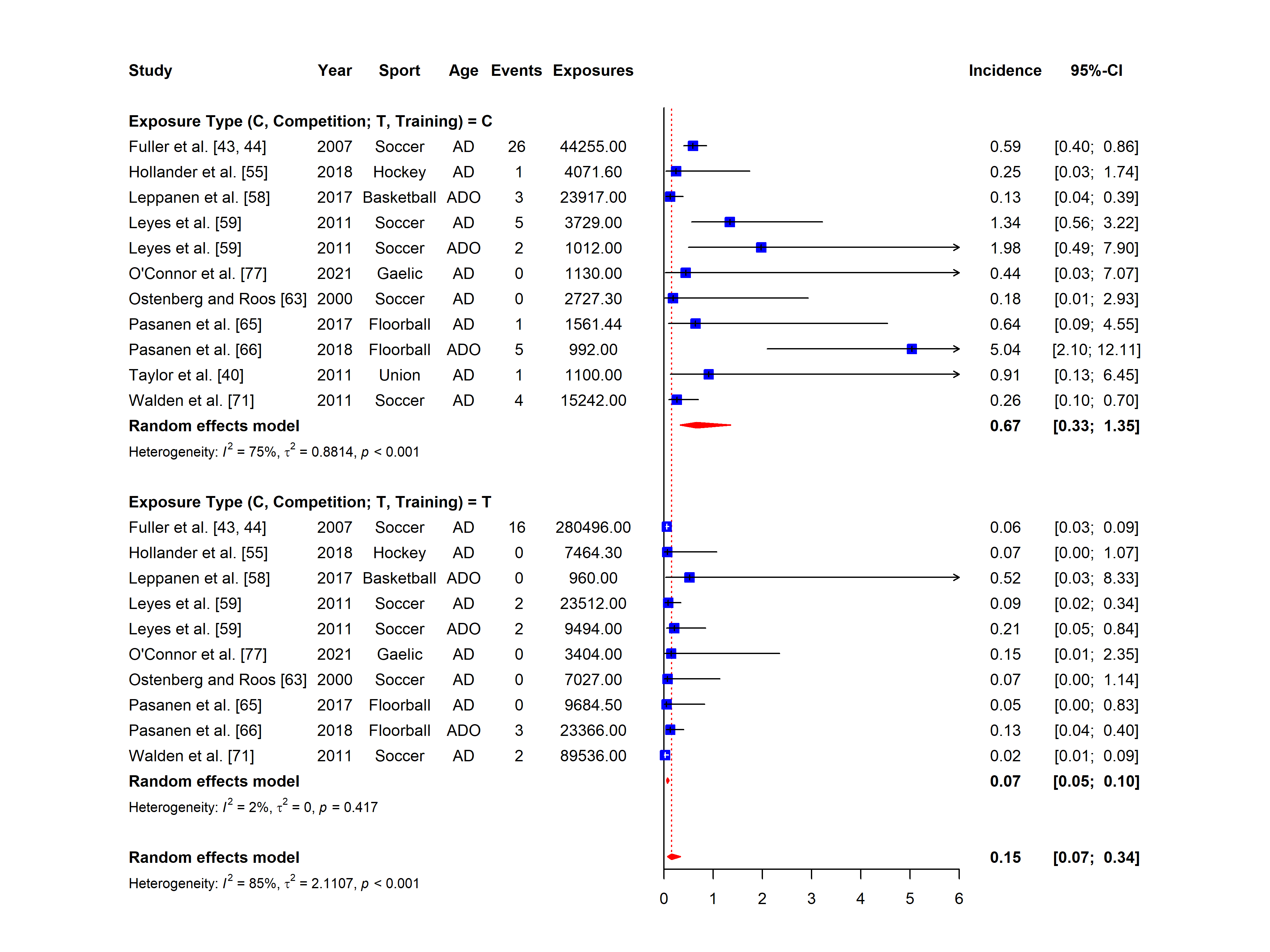


**Figure A11-3** Forest plot of meta-analysis of incidence of non-contact ACL injuries per 1000 player-hours in females by exposure type


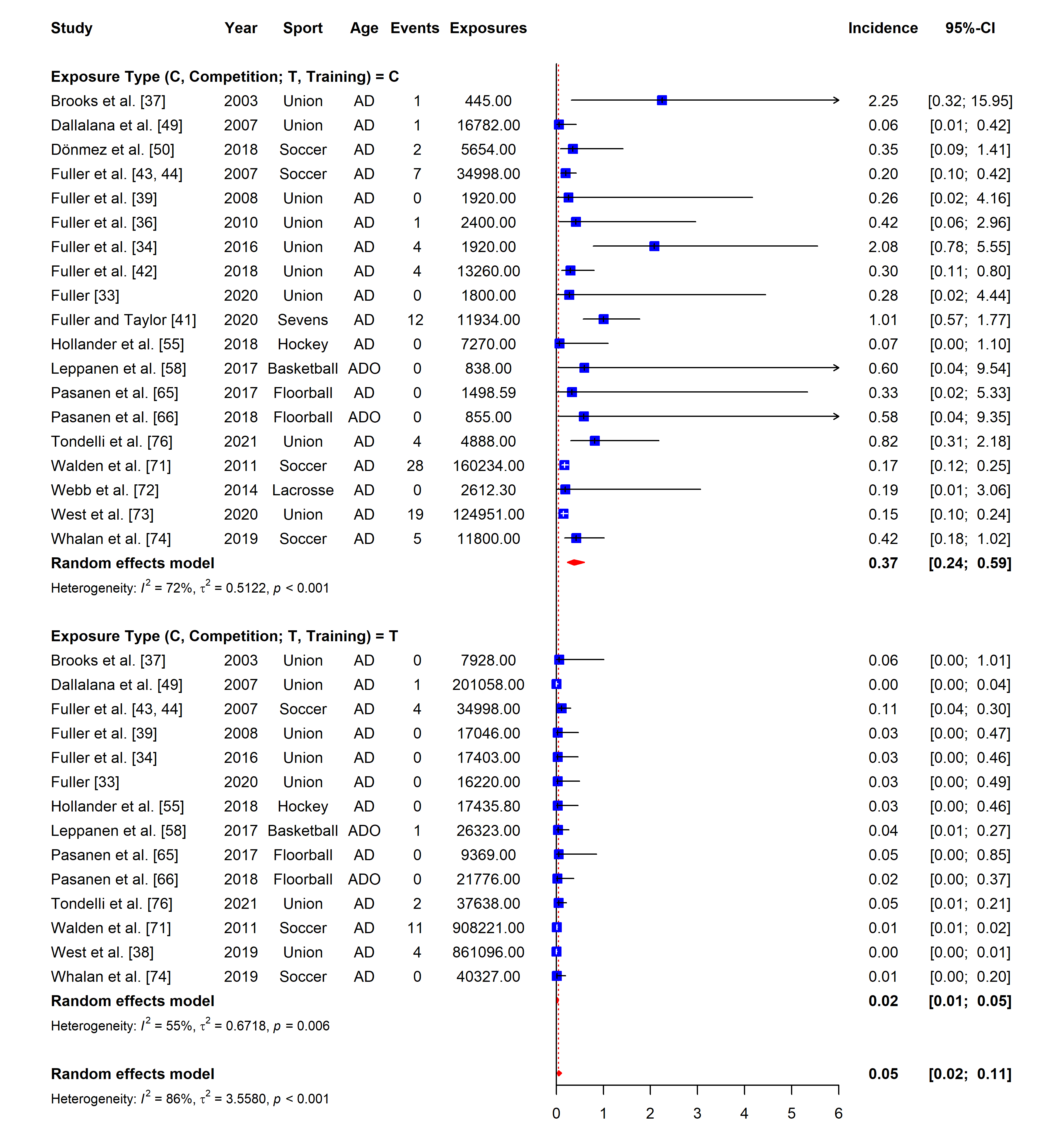


**Figure A11-4** Forest plot of meta-analysis of incidence of non-contact ACL injuries per 1000 player-hours in males by exposure type


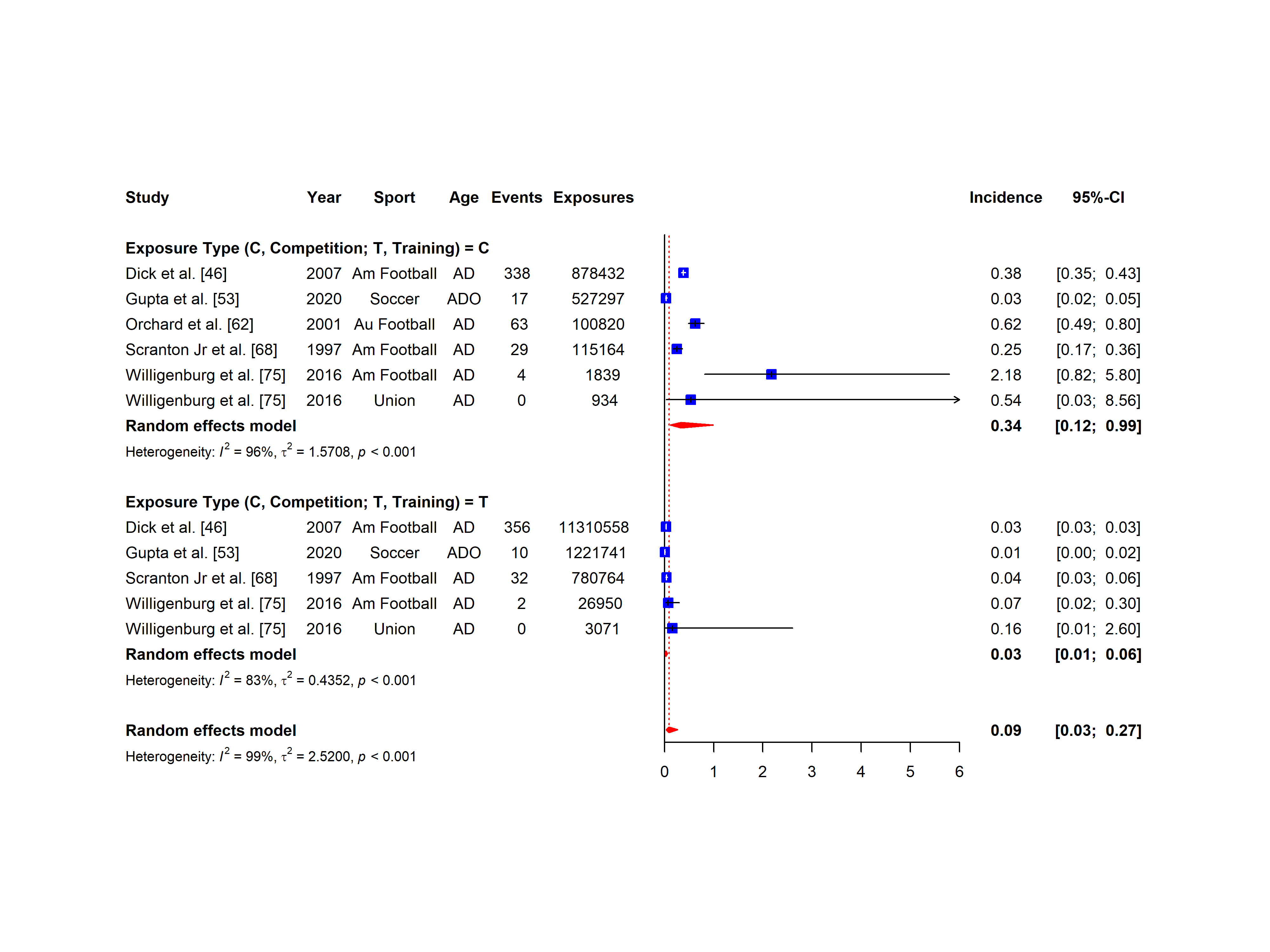


**Figure A11-5** Forest plot of meta-analysis of incidence of non-contact ACL injuries per 1000 player-exposures in males by exposure type

**A12 ADDITIONAL SUB-GROUP ANALYSES**


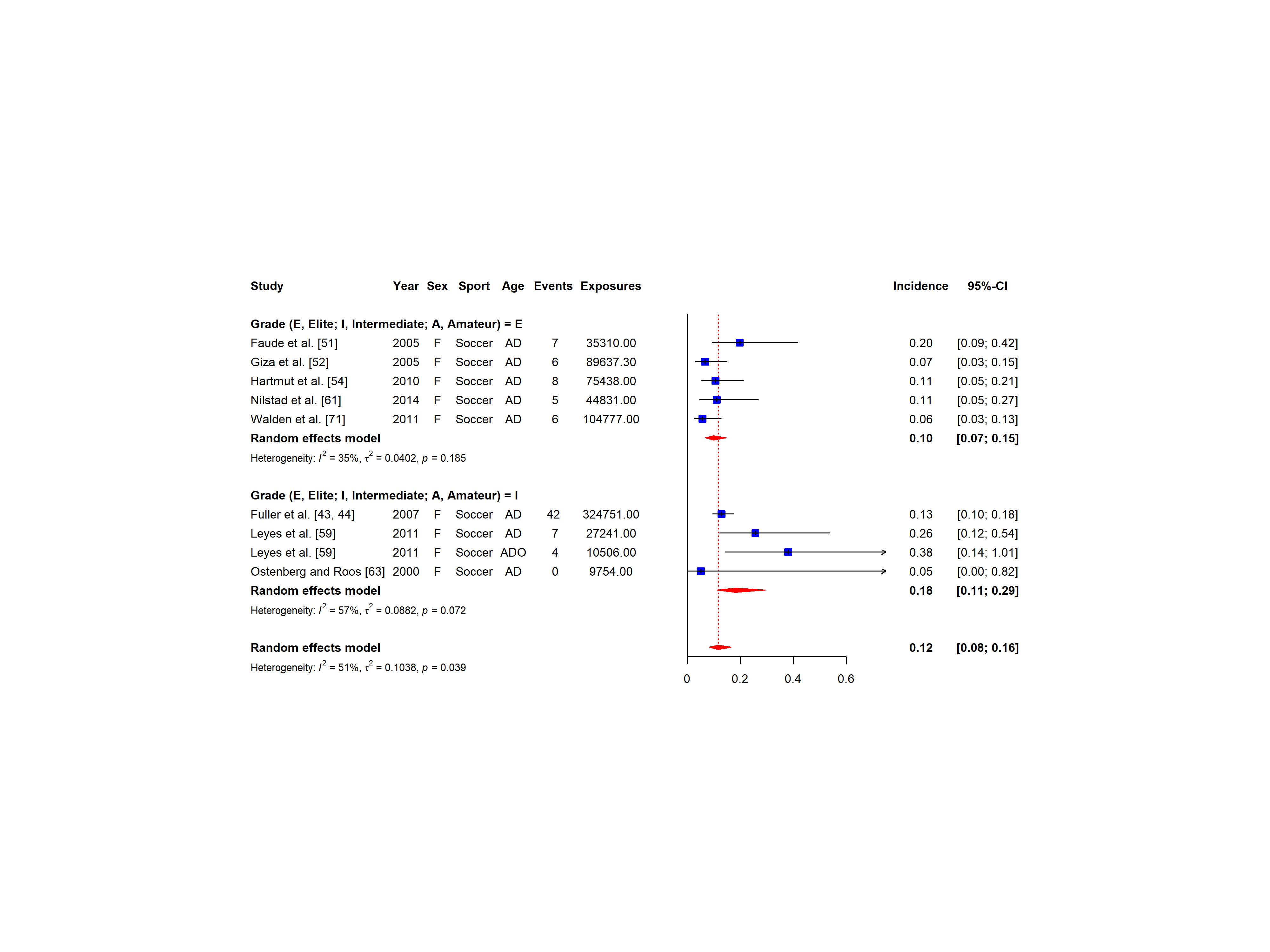


**A12** Forest plot of meta-analysis of incidence of non-contact ACL injuries per 1000 player-exposures in female soccer players by participation level
