# Supplementary material for: Non-contact Anterior Cruciate Ligament Injury Epidemiology in Team-Ball Sports: A Systematic Review with Meta-analysis by Sex, Age, Sport, Participation Level, and Exposure Type
Source: Sports Med. 2022 May 27;52(10):2447–67. doi: 10.1007/s40279-022-01697-w (PMC9136558; doi:10.1007/s40279-022-01697-w)
Supplement: Supplementary file 2 — Supplementary file2 (DOCX 45 KB) [file 40279_2022_1697_MOESM2_ESM.docx]

|  | **CONTENTS** |  |
| --- | --- | --- |
|  |  |  |
| B1 | Meta-Regression (Proportion) ...…………………………………….…………………………... | 2 |
| B2 | Meta-Regression (Incidence) …..……………………………………………………………...... | 3 |
|  |  |  |

**B1 META-REGRESSION (PROPORTION)**

| **VARIABLE** | **FOREST PLOT** | **β (95% CI)** | **SE** | **P-VALUE** |
| --- | --- | --- | --- | --- |
| **SEX** |  |  |  |  |
| Female | Fig. 2 | 1 | - | - |
| Male |  | NS | NS | NS |
| **SPORT** |  |  |  |  |
| American football | A4-1 | 1 | - | - |
| Basketball |  | NS | NS | NS |
| Hockey |  | *Insufficient studies* |  |  |
| Floorball |  | 0.29 (0.03, 0.55) | 0.13 | 0.03* |
| Soccer |  | NS | NS | NS |
| Rugby union |  | NS | NS | NS |
| Lacrosse |  | *Insufficient studies* |  |  |
| SPORT, SEX |  |  |  |  |
| Soccer |  |  |  |  |
| Female athletes | A4-2 | 1 | - | - |
| Male athletes |  | NS | NS | NS |
| **AGE GROUP** |  |  |  |  |
| Adult | A5-1 | 1 | - | - |
| Adolescent |  | NS | NS | NS |
| AGE GROUP, SEX |  |  |  |  |
| Female |  |  |  |  |
| Adult | A5-2 | 1 | - | - |
| Adolescent |  | NS | NS | NS |
| Male |  |  |  |  |
| Adult | A5-3 | 1 | - | - |
| Adolescent |  | NS | NS | NS |
| **PARTICIPATION LEVEL** |  |  |  |  |
| Amateur | A6-1 | 1 | - | - |
| Intermediate |  | NS | NS | NS |
| Elite |  | NS | NS | NS |
| PARTICIPATION LEVEL, SEX |  |  |  |  |
| Elite |  |  |  |  |
| Female athletes | A6-2 | 1 | - | - |
| Male athletes |  | NS | NS | NS |
| Intermediate |  |  |  |  |
| Female athletes | A6-3 | 1 | - | - |
| Male athletes |  | NS | NS | NS |
| Amateur |  |  |  |  |
| Female athletes | A6-4 | 1 | - | - |
| Male athletes |  | NS | NS | NS |
| **EXPOSURE TYPE** |  |  |  |  |
| Competition | A6-4 | 1 | - | - |
| Training |  | NS | NS | NS |
| EXPOSURE TYPE, SEX |  |  |  |  |
| Competition |  |  |  |  |
| Female athletes | A7-2 | 1 | - | - |
| Male athletes |  | -0.22 (-0.42, -0.02) | 0.10 | 0.03* |
| Training |  |  |  |  |
| Female athletes | A7-3 | 1 | - | - |
| Male athletes |  | NS | NS | NS |

CI, confidence interval; SE, standard error of β; * significant p-value ≤ 0.05

**B1** Meta-regression results for proportion of non-contact to total ACL injuries

**B2 META-REGRESSION (INCIDENCE)**

| **VARIABLE** | **FOREST PLOT** | **Β (95% CI)** | **SE** | **P-VALUE** |
| --- | --- | --- | --- | --- |
| **SEX, EXPOSURE UNIT** |  |  |  |  |
| Player-hours |  |  |  |  |
| Female | Fig. 3 | 1 | - | - |
| Male |  | -1.15 (-1.58, -0.73) | 0.22 | 0.03* |
| Player-exposures |  |  |  |  |
| Female | Fig. 4 | 1 | - | - |
| Male |  | NS | NS | NS |
| **SPORT, EXPOSURE UNIT** |  |  |  |  |
| Player-hours |  |  |  |  |
| Floorball | A15 | 1 | - | - |
| Soccer |  | NS | NS | NS |
| Union |  | NS | NS | NS |
| Player-exposures |  |  |  |  |
| Am Football | A16 | 1 | - | - |
| Soccer |  | NS | NS | NS |
| Union |  | NS | NS | NS |
| Basketball |  | NS | NS | NS |
| SPORT, SEX, EXPOSURE UNIT |  |  |  |  |
| Soccer |  |  |  |  |
| Player-hours |  |  |  |  |
| Female | A8-3 | 1 | - | - |
| Male |  | -1.09 (-1.38, -0.81) | 0.14 | < 0.01* |
| Player-exposures |  |  |  |  |
| Female | A8-4 | 1 | - | - |
| Male |  | NS | NS | NS |
| Basketball |  |  |  |  |
| Player-exposure |  |  |  |  |
| Female | A8-5 | 1 | - | - |
| Male |  | -1.34 (-2.25, -0.43) | 0.46 | <0.01* |
| **AGE GROUP, EXPOSURE UNIT** | |  |  |  |
| Player-hours |  |  |  |  |
| Adults | A9-1 | 1 | - | - |
| Adolescents |  | NS | NS | NS |
| Player-exposures |  |  |  |  |
| Adults | A9-2 | 1 | - | - |
| Adolescents |  | -1.28 (-1.87, -0.69) | 0.30 | <0.01* |
| SEX, AGE GROUP, EXPOSURE UNIT |  |  |  |  |
| Adult |  |  |  |  |
| Player-hours |  |  |  |  |
| Female | A9-3 | 1 | - | - |
| Male |  | -1.08 (-1.35, -0.81) | 0.14 | <0.01* |
| Player-exposures |  |  |  |  |
| Female | A9-4 | 1 | - | - |
| Male |  | -0.69 (-1.29, -0.10) | 0.30 | 0.02* |
| Adolescent |  |  |  |  |
| Player-hours |  |  |  |  |
|  | A10-1 | *Insufficient studies* |  |  |
| Player-exposures |  |  |  |  |
| Female | A10-2 | 1 | - | - |
| Male |  | 1.03 (0.26, 1.79) | 0.39 | <0.01* |
| **PARTICIPATION LEVEL, EXPOSURE UNIT** | |  |  |  |
| Player-hours |  |  |  |  |
| Amateur | A28 | 1 | - | - |
| Intermediate |  | NS | NS | NS |
| Elite |  | NS | NS | NS |
| Player-exposures |  |  |  |  |
| Amateur | A29 | 1 | - | - |
| Intermediate |  | 1.44 (0.75, 2.13) | 0.35 | <0.01* |
| Elite |  | *Insufficient studies* |  |  |
| PARTICIPATION LEVEL, SEX, EXPOSURE UNIT | |  |  |  |
| Elite |  |  |  |  |
| Player-hours |  |  |  |  |
| Female | A10-3 | 1 | - | - |
| Male |  | -0.91 (-1.31, -0.52) | 0.20 | <0.01* |
| Intermediate |  |  |  |  |
| Player-hours |  |  |  |  |
| Female | A10-4 | 1 | - | - |
| Male |  | -1.32 (-1.85, -0.80) | 0.27 | <0.01* |
| Player-exposures |  |  |  |  |
| Female | A10-5 | 1 | - | - |
| Male |  | NS | NS | NS |
| Amateur |  |  |  |  |
| Player-hours |  |  |  |  |
| Female | A10-6 | 1 | - | - |
| Male |  | NS | NS | NS |
| Player-exposures |  |  |  |  |
| Female | A10-7 | 1 | - | - |
| Male |  | NS | NS | NS |
| **EXPOSURE TYPE, EXPOSURE UNIT** | |  |  |  |
| Player-hours |  |  |  |  |
| Competition | A11-1 | 1 | - | - |
| Training |  | -2.83 (-3.59, -2.06) | 0.39 | <0.01* |
| Player-exposures |  |  |  |  |
| Competition | A11-2 | 1 | - | - |
| Training |  | -2.59 (-3.70, -1.49) | 0.56 | <0.01* |
| EXPOSURE TYPE, SEX, EXPOSURE UNIT | |  |  |  |
| Female |  |  |  |  |
| Player-hours |  |  |  |  |
| Competition | A11-3 | 1 | - | - |
| Training |  | -2.36 (-3.42, -1.30) | 0.54 | <0.01* |
| Male |  |  |  |  |
| Player-hours |  |  |  |  |
| Competition | A11-4 | 1 | - | - |
| Training |  | -3.12 (-4.07, -2.17) | 0.48 | <0.01* |
| Player-exposures |  |  |  |  |
| Competition | A11-5 | 1 | - | - |
| Training |  | -2.41 (-3.83, -0.99) | 0.72 | <0.01* |
| **OTHER SUB-GROUP ANALYSES** | |  |  |  |
| SPORT, SEX, PARTICIPATION LEVEL, EXPOSURE UNIT | | |  |  |
| Soccer |  |  |  |  |
| Females |  |  |  |  |
| Elite | A12-1 | 1 | - | - |
| Intermediate |  | 0.44 (<0.01, 0.88) | 0.22 | 0.05* |
| Amateur |  | *Insufficient studies* |  |  |

CI, confidence interval; SE, standard error of β; * significant p-value ≤ 0.05; NS, not significant according to p-value

**B2** Meta-regression results for the incidence of non-contact ACL injuries

Proportion
